# Potential interactions between direct oral anticoagulants and atorvastatin/simvastatin: a cohort and case-crossover study

**DOI:** 10.3399/BJGP.2024.0349

**Published:** 2025-05-20

**Authors:** Angel YS Wong, Charlotte Warren-Gash, Krishnan Bhaskaran, Clémence Leyrat, Amitava Banerjee, Liam Smeeth, Ian J Douglas

**Affiliations:** Faculty of Epidemiology and Population Health, London School of Hygiene and Tropical Medicine, London; Faculty of Epidemiology and Population Health, London School of Hygiene and Tropical Medicine, London; Faculty of Epidemiology and Population Health, London School of Hygiene and Tropical Medicine, London; Faculty of Epidemiology and Population Health, London School of Hygiene and Tropical Medicine, London; Institute of Health Informatics, Faculty of Population Health Sciences, University College London UCL, London; Faculty of Epidemiology and Population Health, London School of Hygiene and Tropical Medicine, London; Faculty of Epidemiology and Population Health, London School of Hygiene and Tropical Medicine, London

**Keywords:** hydroxymethylglutaryl-CoA reductase inhibitors, anticoagulants, atorvastatin, simvastatin, drug interactions, primary health care

## Abstract

**Background:**

Direct oral anticoagulants (DOACs) are commonly co-prescribed with statins. Although biologically plausible, whether there is a drug interaction between DOACs and atorvastatin/simvastatin is unclear.

**Aim:**

To investigate the association between co-prescribed DOACs and atorvastatin/simvastatin and bleeding, cardiovascular disease, and mortality.

**Design and setting:**

Cohort and case-crossover study using data from English general practices in the Clinical Practice Research Datalink Aurum from 1 January 2011 to 31 December 2019.

**Method:**

A cohort design was used to estimate hazard ratios for clinically relevant pharmacological interaction safety outcomes (intracranial bleeding, gastrointestinal bleeding, and other bleeding) comparing DOACs and atorvastatin/simvastatin with DOACs and other statins (fluvastatin, pravastatin, and rosuvastatin that are not anticipated to interact with DOACs). Effectiveness outcomes (ischaemic stroke, myocardial infarction, venous thromboembolism, cardiovascular mortality, and all-cause mortality) were also included. In addition, a case-crossover design was used to compare the odds of exposure to different drug initiation patterns in the hazard window versus the referent window within an individual.

**Results:**

Of 397 459 patients who were prescribed DOACs, 70 318 people co-prescribed atorvastatin and 38 724 co-prescribed simvastatin were selected. The cohort analysis showed no difference in risk of all outcomes comparing patients prescribed DOACs and atorvastatin/simvastatin versus those prescribed DOACs and other statins. In the case-crossover analysis, odds ratios (ORs) for other bleeding (OR 5.06, 99% confidence interval [CI] = 3.79 to 6.76) among those initiating DOACs while taking atorvastatin and the ORs for gastrointestinal bleeding (OR 6.05, 99% CI = 4.28 to 8.54) and other bleeding (OR 6.81, 99% CI = 4.74 to 9.78) among those initiating DOACs while taking simvastatin were greater than those initiating DOAC monotherapy. Similar patterns were also observed for cardiovascular mortality and all-cause mortality.

**Conclusion:**

This study shows no evidence of interaction between DOACs and atorvastatin/simvastatin. However, people starting a DOAC while taking atorvastatin/simvastatin were at high risk of bleeding and mortality, likely because of temporal clinical vulnerability.

## Introduction

Direct oral anticoagulants (DOACs) are commonly used for the prevention of arterial embolism among patients with atrial fibrillation and acute coronary syndromes, and the treatment and prevention of venous thromboembolism (VTE). Patients requiring anticoagulation are usually older and often have chronic diseases, including coronary artery disease, resulting in polypharmacy.^1^–^3^ Statins including atorvastatin and simvastatin are also commonly used as a primary and secondary prevention of cardiovascular disease.^4^,^5^

Notably, DOACs are substrates for the efflux transporter P-glycoprotein and are metabolised by the cytochrome P450 system (CYP3A4 enzymes).^6^ As atorvastatin and simvastatin are P-glycoprotein competitors and CYP3A4 inhibitors,^7^,^8^ their co-prescription with DOACs might lead to an increased risk of drug–drug interactions. Therefore, any clinically relevant interaction with atorvastatin/simvastatin would be expected to increase the risk of DOAC side effects, particularly bleeding. As the hypothesised mechanism of interaction would not reduce DOAC levels, the authors of the present study did not anticipate any major impact on DOAC effectiveness outcomes; however, whether these biologically plausible drug interactions ultimately lead to clinical effects is still unclear because of conflicting and limited clinical evidence.^9^–^12^ This study, using two study designs, therefore aimed to investigate the risk of serious clinical outcomes associated with the combined use of DOACs and atorvastatin/simvastatin utilising routine clinical data in England.

**How this fits in**

Direct oral anticoagulants (DOACs) are commonly co-prescribed with statins. Although biologically plausible, whether there is a drug interaction between DOACs and atorvastatin/simvastatin is unclear. This study analysed data from 70 318 patients who used DOACs and were co-prescribed atorvastatin and 38 724 patients who were co-prescribed simvastatin in England. The results suggest that the use of different statins is unlikely to influence the risk of bleeding, cardiovascular disease, and mortality in people using DOACs. However, healthcare providers and patients need to be alert to the elevated risks of bleeding and mortality that were only observed in the specific situation of starting a DOAC while taking a statin.

## Method

### Study design

Cohort and case-crossover studies (see Supplementary Figures S1 and S2) were undertaken. Supplementary Information S1 summarises that the reporting of the study was in accordance with REporting of studies Conducted using Observational Routinely-collected Data (RECORD) reporting guidelines.^13^

### Data source

Data from the Clinical Practice Research Datalink (CPRD) Aurum was used in the study. It contains primary care records of >13 million currently registered patients from 1491 general practices in the UK using EMIS software systems. It is broadly representative in terms of age and sex of the general population.^14^ Linked death data from the Office for National Statistics, hospital admissions data from Hospital Episode Statistics, and individual-level and practice-level deprivation data from the Index of Multiple Deprivation were also used in the study.

### Cohort study

#### Exposure

The study selected people aged ≥18 years receiving their first DOACs (dabigatran, rivaroxaban, apixaban, and edoxaban) with acceptable research quality records in CPRD Aurum from 1 January 2011 to 31 December 2019. To ensure reliable measures of drug use and baseline covariates, all participants had ≥1-year continuous registration before the first recorded DOAC prescription. Atorvastatin and simvastatin were defined as the precipitant drug that was hypothesised to alter the effects of DOACs as they are P-glycoprotein competitors and cytochrome P450 3A4 inhibitors.^7^,^8^

The exposure was defined as concurrent prescription of DOAC with atorvastatin or simvastatin (for example, DOAC and atorvastatin) and was compared with concurrent prescription of DOAC with other statins (fluvastatin, pravastatin, and rosuvastatin). Other statins was selected as the comparison group as they share similar indications with atorvastatin/simvastatin and are not anticipated to interact with DOACs.^7^,^8^ People with any warfarin prescription before cohort entry were excluded to remove a carryover effect of warfarin. The duration of drug prescriptions was calculated (see Supplementary Information S2) and used to determine the exposure groups (see Supplementary Figure S1).

#### Outcomes

Safety outcomes were intracranial bleeding, gastrointestinal bleeding, and other bleeding (any bleeding other than intracranial and gastrointestinal bleeding). Effectiveness outcomes included ischaemic stroke, myocardial infarction (MI), VTE, cardiovascular mortality, and all-cause mortality during the follow-up (details in Supplementary Information S2).

Both groups were followed until the earliest of discontinued treatment of either drug (DOAC/atorvastatin/simvastatin), drug switching to warfarin, switching between atorvastatin/simvastatin and active comparators, outcome occurrence, death, transfer out of the practice, last data-collection date for the practice, or end of the study (31 December 2019).

#### Covariates

Potential confounders and predictors of outcomes^15^ were selected as propensity score covariates using a directed acyclic graph (see Supplementary Figures S3–S5).

#### Statistical analyses

To reduce bias because of heterogeneity between exposure groups, propensity scores were used. Propensity scores were derived from logistic regression, to represent the probability of exposure given the covariates measured on the first day of follow-up. Weights were calculated as the inverse of the propensity score of the treatment received for estimating average treatment effects. Covariate balance was assessed after weighting using standardised differences for each covariate. Hazard ratios (HRs) were computed using inverse probability-of-treatment-weighted Cox regressions with robust standard errors and 99% confidence interval (CI) to handle multiple testing. Multiple imputation through chained equations was performed with 10 imputed datasets to address the data missingness. The treatment effect from each imputed dataset were estimated and these were combined using Rubin’s rules. The cohort was restricted to those individuals whose propensity scores were within the overlapping region of the distributions of the DOAC and precipitant drug group and the comparison group.^16^

#### Subgroup analyses

Analyses were stratified by age, sex, indications, level of DOAC dose (using strength as proxy) in people with atrial fibrillation, individual DOACs, degree of polypharmacy, bodyweight, drug initiation pattern, and kidney function.

#### Sensitivity analyses

A DOAC alone group was included as the comparison group, defined as person-time when a DOAC but not atorvastatin/simvastatin was prescribed.

### Modified case-crossover study

The case-crossover design eliminates time-invariant confounding as all comparisons are within the individual.^17^ It only includes individuals who experienced the outcome and compares each individual’s exposure in a period before the outcome (hazard window) to the exposure during an earlier control period (referent window).^18^

The authors selected people who experienced the specific outcome with acceptable research quality records and who were exposed to DOACs and/or atorvastatin/simvastatin before the outcome during a valid follow-up. People aged <18 years at start of follow-up were excluded. The follow-up started from the latest of study start date (1 January 2011); or at least 1-year continuous registration of GP practices until outcome occurrence, death, transfer out of the practice, last data collection date for the practice, or end of the study (31 December 2019) (see Supplementary Figure S2). Only the first event was included (see Supplementary Information S3).

The hazard window started from days 1 to 30 on/before the outcome occurrence, and the control window started from days 91 to 120 before the outcome occurrence. A 60-day washout period was included to avoid autocorrelation in exposure between periods and carryover effects.

Conditional logistic regression was used to estimate the odds ratios (ORs) for all outcomes associated with different drug initiation patterns using the six-parameter model, conditioned on individual with 99% CI to handle multiple testing. Supplementary Information S4 shows the considerations of interpretations.

#### Subgroup analyses

Different doses of DOAC and types of DOACs were investigated as subgroup analyses.

#### Sensitivity analyses

Seven-day and 90-day hazard and referent windows were used to investigate the sensitivity of results to risk-period length. A *post-hoc* sensitivity analysis was conducted by repeating the analyses for concomitant use of DOACs and other statins.

Stata/MP (versions 17 and 18) and RStudio (version 2021.09.0) was used for data processing and analyses.

## Results

In total, 397 459 people were prescribed a DOAC. Compared with those prescribed other statins (*n* = 8577), those in the DOAC and atorvastatin group (*n* = 70 318) were more likely to be younger, have higher levels of deprivation, higher level of alcohol consumption, but with similar proportions of comorbidities and medications used in the past 3 months (see Supplementary Figure S6 and Supplementary Table S1).

Compared with those prescribed other statins (*n* = 8922), the DOAC and simvastatin group (*n* = 38 724) were more likely to be older, have higher levels of deprivation, but with similar proportions of comorbidities and medications used in the past 3 months (see Supplementary Figure S6 and Supplementary Table S2).

Individuals who were taking DOAC and atorvastatin/simvastatin tended to have fewer GP active consultations in the past year than those in the DOAC and other statins group. Standardised differences for each outcome are shown in Supplementary Tables S3–S7.

In the case-crossover studies (see Supplementary Figure S7), 130 674 people who had an ischaemic stroke, 154 598 with MI, 135 808 with VTE, 44 124 with intracranial bleeding, 297 041 with gastrointestinal bleeding, 359 857 with other bleeding, 191 682 with cardiovascular death, and 832 373 people who died were selected.

### Atorvastatin

**Figure 1:**
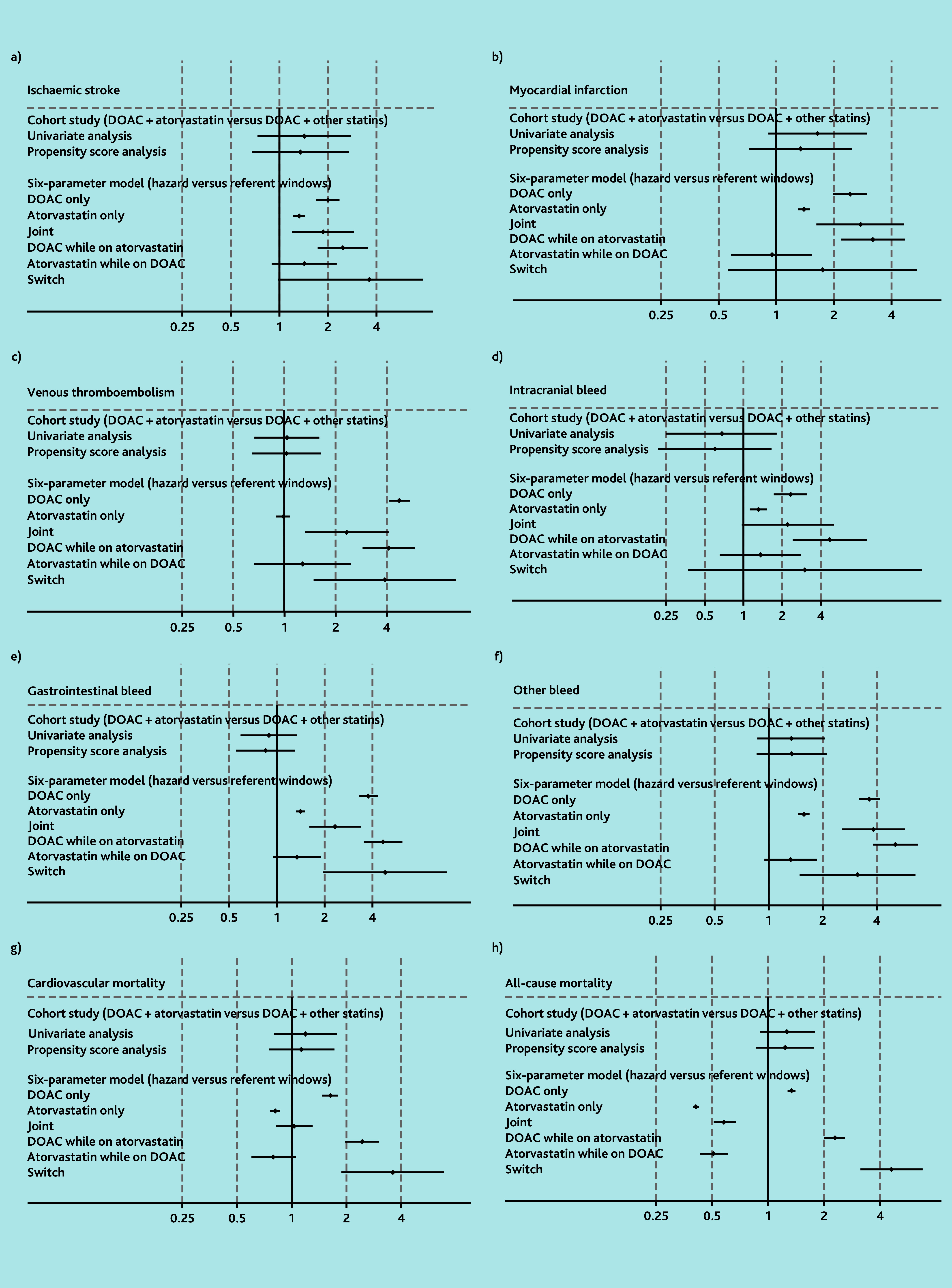
Results for DOACs and atorvastatin using cohort study design and six-parameter case-crossover study design (see Supplementary Figure S8 for a more detailed version of this figure that includes the details of the point estimates). a) Ischaemic stroke; b) myocardial infarction; c) venous thromboembolism; d) intracranial bleed; e) gastrointestinal bleed; f) other bleed; g) cardiovascular mortality; and h) all-cause mortality. In the six-parameter model joint indicates initiation of both drugs simultaneously in hazard or control window. DOAC while prescribed atorvastatin indicates initiation of DOACs in the presence of atorvastatin. Atorvastatin while prescribed DOAC indicates initiation of atorvastatin in the presence of DOACs. Switch indicates use of one drug in the hazard window and the other drug in the control window. DOAC = direct oral anticoagulants.

In the cohort analysis, no evidence of an increased risk of either safety or effectiveness outcomes were observed. HRs ranged from 0.60 (99% CI = 0.22 to 1.65) for intracranial bleeding to 1.35 for ischaemic stroke (99% CI = 0.67 to 2.71) or other bleeding (99% CI = 0.86 to 2.11), with CIs all including 1 (Figure 1 [see Supplementary Figure S8 for a more detailed version of Figure 1], Supplementary Figure S9, and Supplementary Table S8).

In the case-crossover analysis, the ORs for other bleeding and mortality outcomes among those initiating a DOAC while taking atorvastatin (ORs ranging from 2.28 [99% CI = 2.00 to 2.58] for all-cause mortality to 5.06 [99% CI = 3.79 to 6.76] for other bleeding) were greater than those observed with DOAC monotherapy. However, no increased odds of these outcomes were observed for people initiating atorvastatin while taking a DOAC (Figure 1 and Supplementary Figure S8).

### Simvastatin

**Figure 2:**
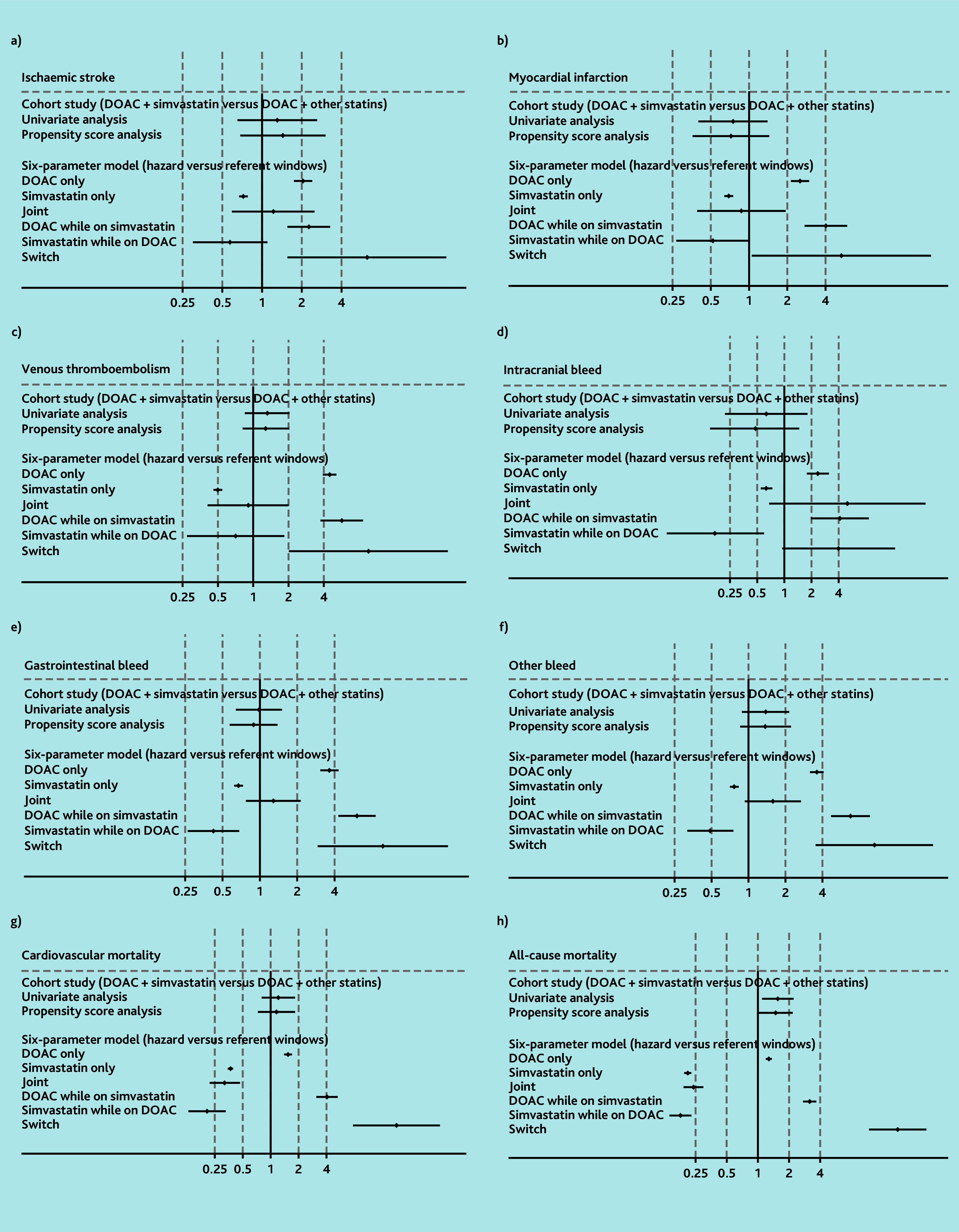
Results for DOACs and simvastatin using cohort study design and six-parameter case-crossover study design (see Supplementary Figure S10 for a more detailed version of this figure that includes the details of the point estimates). a) Ischaemic stroke; b) myocardial infarction; c) venous thromboembolism; d) intracranial bleed; e) gastrointestinal bleed; f) other bleed; g) cardiovascular mortality; and h) all-cause mortality. In the six-parameter model joint indicates initiation of both drugs simultaneously in hazard or control window. DOAC while prescribed simvastatin indicates initiation of DOACs in the presence of simvastatin. Simvastatin while prescribed DOAC indicates initiation of simvastatin in the presence of DOACs. Switch indicates use of one drug in the hazard window and the other drug in the control window. DOAC = direct oral anticoagulants.

In the cohort analysis, there was no evidence of increased risk of all outcomes except all-cause mortality, comparing DOAC and simvastatin versus DOAC and other statins. HRs ranged from 0.48 (99% CI = 0.15 to 1.47) for intracranial bleeding to 1.44 (99% CI = 0.68 to 3.04) for ischaemic stroke, with CIs crossing 1 (Figure 2 [see Supplementary Figure S10 for a more detailed version], Supplementary Figure S11, and Supplementary Table S9). Although an increased risk of all-cause mortality associated with DOAC and simvastatin was observed, compared with DOAC and other statins (HR 1.49, 99% CI = 1.02 to 2.18), finer adjustment for age (categorised as 18–39, 40–49, 50–59, 60–69, 70–79, and ≥80 years) weakened this association (HR 1.44, 99% CI = 0.98 to 2.10) (data not shown).

In the case-crossover analysis, it was observed that the odds of bleeding and mortality outcomes (except intracranial bleeding) among those initiating a DOAC while taking simvastatin (ORs ranging from 3.18 [99% CI = 2.75 to 3.68] for all-cause mortality, 6.05 [99% CI = 4.28 to 8.54] for gastrointestinal bleeding, to 6.81 [99% CI = 4.74 to 9.78] for other bleeding) were greater than those for DOAC monotherapy. However, no similar pattern was seen among people initiating simvastatin while receiving a DOAC (Figure 2 and Supplementary Figure S10).

### Subgroup analyses

Results in the cohort analysis were similar to the main analysis (see Supplementary Tables S10–S16).

In case-crossover analysis, the odds for all outcomes did not increase with higher DOAC dose in people with atrial fibrillation (see Supplementary Figures S12 and S13). It was observed that an increased odds of mortality associated with an initiation of DOACs while taking atorvastatin, greater than those for DOAC monotherapy, differed by type of DOAC, including cardiovascular mortality (apixaban = 2.94) and all-cause mortality (rivaroxaban = 1.57 and apixaban = 2.90) (see Supplementary Figure S14). Similarly, the odds of some outcomes associated with initiating DOACs while taking simvastatin were greater than the odds for DOAC monotherapy; that also differed by types of DOACs including gastrointestinal bleeding (apixaban = 6.86), other bleeding (rivaroxaban = 5.86), cardiovascular mortality (rivaroxaban = 3.03 and apixaban = 5.41), and all-cause mortality (dabigatran = 1.58, rivaroxaban = 2.50, apixaban = 3.87, and edoxaban = 6.10) (see Supplementary Figure S15).

### Sensitivity analyses

In the cohort analysis, using the DOAC alone group as the comparison group produced similar results to the main analysis using an active comparator as the comparison group (see Supplementary Tables S8 and S9). Notably, no increased risk of all-cause mortality was observed in DOAC and simvastatin versus DOAC alone.

In the case-crossover analysis, some evidence was observed of greater ORs for other bleeding (OR 7.91, 99% CI = 3.47 to 18.03), cardiovascular mortality (OR 2.83, 99% CI = 1.54 to 5.22), and strong evidence of greater odds of all-cause mortality (OR 2.29, 99% CI = 1.61 to 3.25) among those initiating a DOAC while taking statins other than atorvastatin or simvastatin compared with the ORs observed with DOAC monotherapy (see Supplementary Figure S16). For duration of effect (see Supplementary Figure S17), the association with initiating DOACs while taking atorvastatin and synergistic risks were no longer seen for other bleeding in other risk windows. For a 7-day and 90-day risk window, the association between initiating DOACs while taking simvastatin and synergistic risk of myocardial infarction was observed (see Supplementary Figure S18). New associations seen were a synergistic increased risk of ischaemic stroke using a 90-day risk window and increased risk of myocardial infarction using a 7-day risk window when initiating DOAC while taking atorvastatin (see Supplementary Figure S17). An increased risk of intracranial bleeding using a 7-day risk window when initiating DOAC while taking simvastatin was observed (see Supplementary Figure S18).

All other sensitivity analyses showed similar results to the main analyses.

## Discussion

### Summary

Using two complementary study designs, no evidence was observed of a clinically relevant pharmacological interaction between DOACs and statins. Although atorvastatin and simvastatin are P-glycoprotein competitors and moderate cytochrome P450 3A4 inhibitors and could theoretically increase the risk of bleeding, this cohort analysis found no evidence of a higher risk of bleeding associated with co-prescribed DOAC with these statins.

In the case-crossover design only, for atorvastatin and simvastatin an increased risk of bleeding and mortality was observed for people initiating a DOAC while taking statins. No increased risk of these outcomes was seen in people initiating either statin while receiving a DOAC. Although the case-crossover design is an effective design for dealing with between-person differences, it remains vulnerable to confounding by time-varying characteristics. This suggests these adverse outcomes are unlikely to be the result of a pharmacological interaction but are a marker of poor health around the time of DOAC initiation among individuals prescribed statins. It was anticipated that risks of effectiveness outcomes would not be increased through the mechanism owing to drug–drug interaction. The present study showed no difference in risk of effectiveness outcomes except all-cause mortality with DOAC and simvastatin versus DOAC and other statins in cohort analysis. Given that patients who were in the DOAC and simvastatin group were older than patients who were prescribed DOAC and other statins, it might explain the spurious harmful effect of DOAC and simvastatin initially observed in relation to all-cause mortality. After finer age adjustment in addition to using the propensity scores, it was noted that the estimate shifted towards null, supporting that age could not be fully accounted for using the propensity score alone. Moreover, there was no evidence of an elevated risk of all-cause mortality when comparing those who were taking DOAC and simvastatin versus DOAC alone in the sensitivity analysis. However, a similar pattern was observed for other bleeding, cardiovascular mortality, and all-cause mortality; the increased odds when initiating DOACs while taking other statins was greater than the increased odds associated with initiating DOAC monotherapy. It supports the interpretation that the increased odds of other bleeding, cardiovascular mortality, and all-cause mortality were not specific to atorvastatin/simvastatin, and therefore unlikely because of a drug–drug interaction, but possibly time-varying confounding.

### Strengths and limitations

To date, to the best of the authors’ knowledge, this is the first population-based study investigating drug interactions between DOACs and atorvastatin/simvastatin using two study designs in England. With active comparator, propensity scores, and self-controlled designs, it was possible to robustly evaluate the relative risk of clinical outcomes, estimate the absolute risk for public health implications, and reduce confounding.

This study has some limitations. First, drug adherence and persistence were unknown, leading to potential misclassification bias of exposure. Assuming a non-differential misclassification of exposure, estimates would be biased towards null. Second, the study did not have large cohorts for some drug-outcome pairs, specifically for intracranial bleeding. Several subgroup analyses were conducted, but they may be prone to type I error that requires cautious interpretation. Further, the study population was predominantly White so results may not be generalisable to other ethnic groups. There were also missing data, but a multiple imputation approach to handle missing covariate data in propensity scores was used. The data on discontinuing DOAC treatment owing to bleeding was not available to investigate if the risk of thromboembolic events elevated after bleeding. However, only approximately 10% of those who had a first ischaemic stroke had their first ischaemic stroke after bleeding during follow-up. Finally, it was not possible to eliminate residual confounding but the authors attempted to minimise confounding by using a propensity score method and self-controlled design.

### Comparison with existing literature

A case-control study showed higher risk of major bleeding associated with simvastatin or lovastatin compared with other statins in patients who were prescribed dabigatran.^10^ However, they similarly showed no difference in risk of stroke. Notably, atorvastatin was included in the comparison group, which is a CYP3A4 inhibitor, leading to difficulty in result interpretation. Aligning to the findings in the current study, a cohort study reported no evidence of an increased risk of VTE and major bleeding comparing any statins with non-use in patients who were prescribed rivaroxaban, but they did not specifically investigate different types of statins.^11^ Two cohort studies showed a lower risk of major bleeding associated with DOAC and atorvastatin or any statins compared with DOAC alone.^9^,^12^ In contrast, the current study showed no difference in risk of bleeding in the cohort analysis with and without active comparators. To the authors’ knowledge, no previous studies have evaluated the impact of drug initiation patterns and mortality outcomes for the concomitant use of DOACs and statins.

### Implications for research and practice

Atorvastatin and simvastatin are the most widely prescribed statins accounting for 65 million and 14 million prescriptions, respectively, across all GP practices in England in 2023.^19^ The safety of prescribing these statins in people who are prescribed DOACs is important information that reassures prescribers and patients. The current study directly compared atorvastatin and simvastatin with other statins and found no difference in risk for all important clinical outcomes. This suggests that the use of different statins is unlikely to influence the risk of bleeding, cardiovascular disease, and mortality in people who are prescribed DOACs. However, healthcare providers and patients need to be alert when people prescribed atorvastatin/simvastatin initiate a DOAC as these patients were at high risk of developing bleeding and high risk of mortality.

In conclusion, this study found no evidence of a clinically relevant pharmacological interaction between atorvastatin/simvastatin and DOACs. However, people starting a DOAC while taking atorvastatin/simvastatin were at high risk of developing bleeding and mortality, likely because of temporal clinical vulnerability.

## Supplementary Information



## Data Availability

Computing code and study protocol are available from the corresponding author on request for the purposes of reproducing the results. The study data cannot be made available to other researchers because of the terms specified in Data Use Agreements. The codelists identifying the outcomes and covariates are available in LSHTM Data Compass: https://doi.org/10.17037/DATA.00004214.
