# Supplementary material for: Potential interactions between direct oral anticoagulants and atorvastatin/simvastatin: a cohort and case-crossover study
Source: Br J Gen Pract. 2025 May 20;75(756):e466–73. doi: 10.3399/BJGP.2024.0349 (PMC12117505; doi:10.3399/BJGP.2024.0349)
Supplement: Supplementary file 1 [file BJGP.2024.0349_suppl.pdf]

Supplementary material: Potential interactions between direct oral anticoagulants and atorvastatin/simvastatin: cohort and case-crossover study

Contents

Figure S1. Illustration of the cohort design. 4

Figure S2. Illustration of the modified case-crossover design. 6

Supplementary Information S1. RECORD statement 7

Supplementary Information S2. Details of the implementation of cohort study design..14

Figure S3. Directed acyclic diagram to select propensity score covariates for bleeding outcomes. 17

Figure S4. Directed acyclic diagram to select propensity score covariates for cardiovascular outcomes. 18

Figure S5. Directed acyclic diagram to select propensity score covariates for all-cause mortality. 19

Supplementary Information S3. Illustration of drug initiation patterns in case-crossover study design using 6-parameter model 20

Supplementary Information S4. Description of interpretations for case-crossover design 21

Figure S6. Flow diagram for inclusion in cohort study design 22

Table S1. Baseline characteristics of the DOAC + atorvastatin group and DOAC + other statins group before restricting people with overlapping propensity score distributions between groups 23

Table S2. Baseline characteristics of the DOAC + simvastatin group and DOAC + other statins group before restricting people with overlapping propensity score distributions between groups 27

Table S3. Standardised differences for the associations between concomitant use of DOACs and atorvastatin and bleeding outcomes using active comparator in cohort study 31

Table S4. Standardised differences for the associations between concomitant use of DOACs and atorvastatin and cardiovascular outcomes using active comparator in cohort study 34

Table S5. Standardised differences for the associations between concomitant use of DOACs and atorvastatin/simvastatin and all-cause mortality using active comparator in cohort study 37

Table S6. Standardised differences for the associations between concomitant use of DOACs and simvastatin and bleeding outcomes using active comparator in cohort study 40

Table S7. Standardised differences for the associations between concomitant use of DOACs and simvastatin and cardiovascular outcomes using active comparator in cohort study 43

Figure S7. Flow diagram for inclusion in case-crossover study design 46

Figure S8. More detailed version of Figure 1 from the main text 47
Figure S9 Kaplan-Meier curves for the association between DOAC + atorvastatin and different outcomes, versus DOAC + other statins 48
Ischaemic stroke..48
Myocardial infarction..48
Venous thromboembolism 48
Intracranial bleeding 48
Gastrointestinal bleeding 49
Other bleeding...49
Cardiovascular mortality...49
All-cause mortality..50
Table S8. Details of results from univariable and propensity score models for the association between concomitant use of DOACs and atorvastatin and outcomes using active comparator and DOAC alone groups as comparison groups respectively in cohort study 51
Figure S10. More detailed version of Figure 2 from the main text 52
Figure S11. Kaplan-Meier curves for the association between DOAC + simvastatin and different outcomes, versus DOAC + other statins 53
Ischaemic stroke..53
Myocardial infarction..53
Venous thromboembolism 53
Intracranial bleeding 53
Gastrointestinal bleeding 54
Other bleeding...54
Cardiovascular mortality...54
All-cause mortality..55
Table S9. Details of results from univariable and propensity score models for the association between concomitant use of DOACs and simvastatin and outcomes using active comparator and DOAC alone groups as comparison groups respectively in cohort study 56
Table S10. Subgroup analysis for the association between concomitant use of DOAC and atorvastatin/simvastatin and all-cause mortality, compared with DOAC and other statins in cohort study 58
Table S11. Subgroup analysis for the association between concomitant use of DOAC and atorvastatin/simvastatin and cardiovascular mortality, compared with DOAC and other statins in cohort study 59
Table S12. Subgroup analysis for the association between concomitant use of DOAC and atorvastatin/simvastatin and ischaemic stroke, compared with DOAC and other statins in cohort study 60

Table S13. Subgroup analysis for the association between concomitant use of DOAC and atorvastatin/simvastatin and myocardial infarction, compared with DOAC and other statins in cohort study 61
Table S14. Subgroup analysis for the association between concomitant use of DOAC and atorvastatin/simvastatin and venous thromboembolism, compared with DOAC and other statins in cohort study 62
Table S15. Subgroup analysis for the association between concomitant use of DOAC and atorvastatin/simvastatin and gastrointestinal bleeding, compared with DOAC and other statins in cohort study 63
Table S16. Subgroup analysis for the association between concomitant use of DOAC and atorvastatin/simvastatin and other bleeding, compared with DOAC and other statins in cohort study......64
Figure S12. Subgroup analysis by level of dose of DOAC for the concomitant use of DOAC and atorvastatin among people with atrial fibrillation in case-crossover study ..65
Figure S13. Subgroup analysis by level of dose of DOAC for the concomitant use of DOAC and simvastatin among people with atrial fibrillation in case-crossover study...69
Figure S14. Subgroup analysis by different types of DOAC for the concomitant use of DOAC and atorvastatin among people with atrial fibrillation in case-crossover study ..73
Figure S15. Subgroup analysis by different types of DOAC for the concomitant use of DOAC and simvastatin among people with atrial fibrillation in case-crossover study...78
Figure S16. Results of case-crossover study investigating the concomitant use of DOACs and other statins 82
Figure S17. Duration of effects for concomitant use of DOAC and atorvastatin in case-crossover study......86
Figure S18. Duration of effects for concomitant use of DOAC and simvastatin in case-crossover study......90

Figure S1. Illustration of the cohort design.

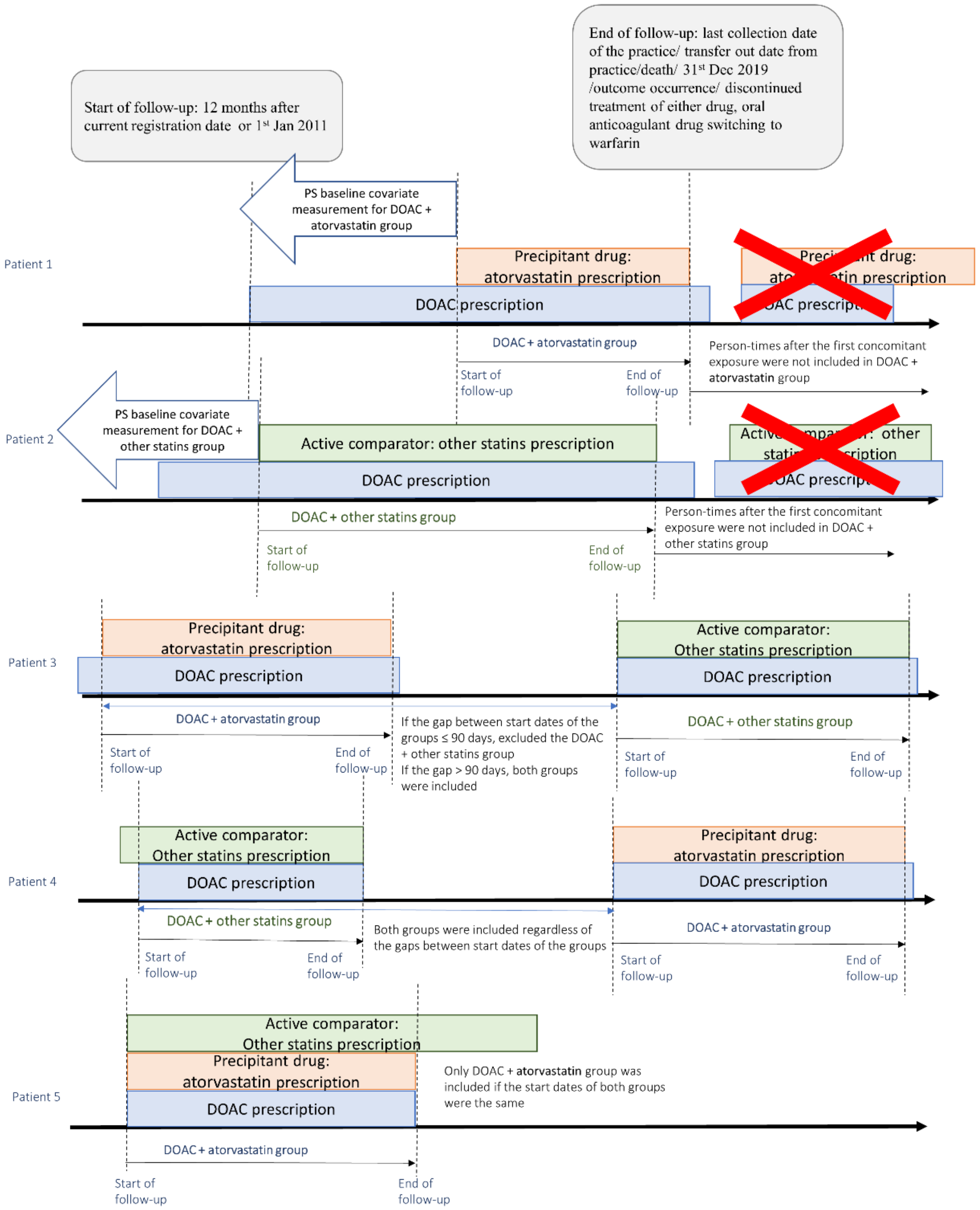

Figure 1a. Examples of inclusion of study cohorts in cohort study design (main analysis)

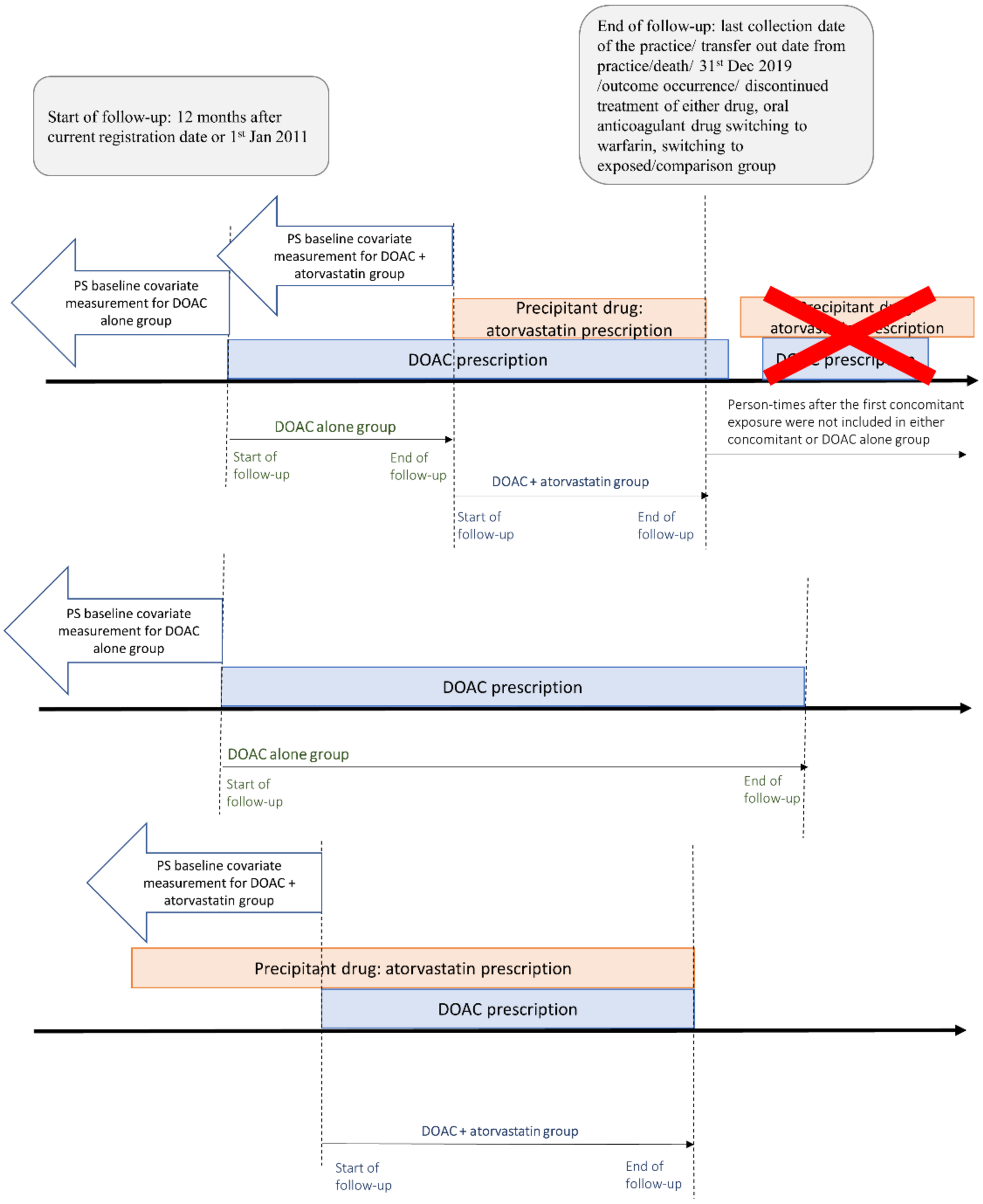

Figure 1b. Examples of inclusion of study cohorts in cohort study design (sensitivity analysis)

Abbreviation: PS, propensity score; CPRD, Clinical Practice Research Datalink; DOAC, Direct oral anticoagulant

Figure S2. Illustration of the modified case-crossover design.

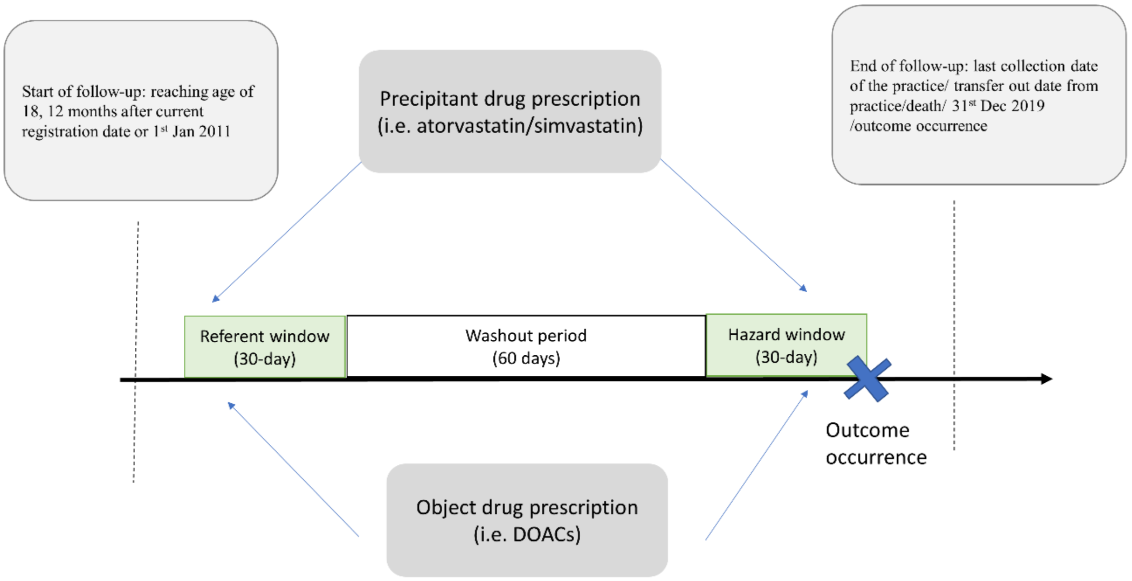

Figure 2. Illustration of modified case-crossover study design in the main analysis

Abbreviation: DOAC, Direct oral anticoagulant

Supplementary Information S1. RECORD statement

	Item No.	STROBE items	Location in manuscript where items are reported	RECORD items	Location in manuscript where items are reported
**Title and abstract**					
	1	(a) Indicate the study's design with a commonly used term in the title or the abstract (b) Provide in the abstract an informative and balanced summary of what was done and what was found		RECORD 1.1: The type of data used should be specified in the title or abstract. When possible, the name of the databases used should be included.<br><br>RECORD 1.2: If applicable, the geographic region and timeframe within which the study took place should be reported in the title or abstract.<br><br>RECORD 1.3: If linkage between databases was conducted for the study, this should be clearly stated in the title or abstract.	1,2
**Introduction**					
Background rationale	2	Explain the scientific background and rationale for the investigation being reported			4
Objectives	3	State specific objectives, including any prespecified hypotheses			4
**Methods**					
Study Design	4	Present key elements of study design early in the paper			4

Setting	5	Describe the setting, locations, and relevant dates, including periods of recruitment, exposure, follow-up, and data collection			4
Participants	6	*(a) Cohort study* - Give the eligibility criteria, and the sources and methods of selection of participants. Describe methods of follow-up<br>*Case-control study* - Give the eligibility criteria, and the sources and methods of case ascertainment and control selection. Give the rationale for the choice of cases and controls<br>*Cross-sectional study* - Give the eligibility criteria, and the sources and methods of selection of participants<br><br>*(b) Cohort study* - For matched studies, give matching criteria and number of exposed and unexposed<br>*Case-control study* - For matched studies, give matching criteria and the number of controls per case		RECORD 6.1: The methods of study population selection (such as codes or algorithms used to identify subjects) should be listed in detail. If this is not possible, an explanation should be provided.<br><br>RECORD 6.2: Any validation studies of the codes or algorithms used to select the population should be referenced. If validation was conducted for this study and not published elsewhere, detailed methods and results should be provided.<br><br>RECORD 6.3: If the study involved linkage of databases, consider use of a flow diagram or other graphical display to demonstrate the data linkage process, including the number of individuals with linked data at each stage.	4-6
Variables	7	Clearly define all outcomes, exposures, predictors, potential confounders, and effect		RECORD 7.1: A complete list of codes and algorithms used to classify exposures, outcomes, confounders,	5

		modifiers. Give diagnostic criteria, if applicable.		and effect modifiers should be provided. If these cannot be reported, an explanation should be provided.	
Data sources/ measurement	8	For each variable of interest, give sources of data and details of methods of assessment (measurement).<br>Describe comparability of assessment methods if there is more than one group			4
Bias	9	Describe any efforts to address potential sources of bias			5-6
Study size	10	Explain how the study size was arrived at			4,6
Quantitative variables	11	Explain how quantitative variables were handled in the analyses. If applicable, describe which groupings were chosen, and why			5-6
Statistical methods	12	(a) Describe all statistical methods, including those used to control for confounding<br>(b) Describe any methods used to examine subgroups and interactions<br>(c) Explain how missing data were addressed<br>(d) *Cohort study* - If applicable, explain how loss to follow-up was addressed<br>*Case-control study* - If applicable, explain how			5-6

		matching of cases and controls was addressed<br>*Cross-sectional study* - If applicable, describe analytical methods taking account of sampling strategy<br>(e) Describe any sensitivity analyses			
Data access and cleaning methods		..		RECORD 12.1: Authors should describe the extent to which the investigators had access to the database population used to create the study population.<br><br>RECORD 12.2: Authors should provide information on the data cleaning methods used in the study.	Material S1
Linkage		..		RECORD 12.3: State whether the study included person-level, institutional-level, or other data linkage across two or more databases. The methods of linkage and methods of linkage quality evaluation should be provided.	4
**Results**					
Participants	13	(a) Report the numbers of individuals at each stage of the study (*e.g.*, numbers potentially eligible, examined for eligibility, confirmed eligible, included in the study,		RECORD 13.1: Describe in detail the selection of the persons included in the study (*i.e.,* study population selection) including filtering based on data quality, data availability and linkage. The selection of included persons can be described in the text	Figure S6 and S7

		completing follow-up, and analysed)<br>(b) Give reasons for non-participation at each stage.<br>(c) Consider use of a flow diagram		and/or by means of the study flow diagram.	
Descriptive data	14	(a) Give characteristics of study participants (*e.g.*, demographic, clinical, social) and information on exposures and potential confounders<br>(b) Indicate the number of participants with missing data for each variable of interest<br>(c) *Cohort study* - summarise follow-up time (*e.g.*, average and total amount)			6, Tables S1, S2
Outcome data	15	*Cohort study* - Report numbers of outcome events or summary measures over time<br>*Case-control study* - Report numbers in each exposure category, or summary measures of exposure<br>*Cross-sectional study* - Report numbers of outcome events or summary measures			6-7
Main results	16	(a) Give unadjusted estimates and, if applicable, confounder-adjusted estimates and their precision (e.g., 95% confidence interval). Make clear which			6-7, Figures 1 and 2

		confounders were adjusted for and why they were included<br>(b) Report category boundaries when continuous variables were categorized<br>(c) If relevant, consider translating estimates of relative risk into absolute risk for a meaningful time period			
Other analyses	17	Report other analyses done—e.g., analyses of subgroups and interactions, and sensitivity analyses			7,8
**Discussion**					
Key results	18	Summarise key results with reference to study objectives			8
Limitations	19	Discuss limitations of the study, taking into account sources of potential bias or imprecision. Discuss both direction and magnitude of any potential bias		RECORD 19.1: Discuss the implications of using data that were not created or collected to answer the specific research question(s). Include discussion of misclassification bias, unmeasured confounding, missing data, and changing eligibility over time, as they pertain to the study being reported.	8-9
Interpretation	20	Give a cautious overall interpretation of results considering objectives, limitations, multiplicity of analyses, results from similar studies, and other relevant evidence			8

Generalisability	21	Discuss the generalisability (external validity) of the study results			9
**Other Information**					
Funding	22	Give the source of funding and the role of the funders for the present study and, if applicable, for the original study on which the present article is based			10
Accessibility of protocol, raw data, and programming code		..		RECORD 22.1: Authors should provide information on how to access any supplemental information such as the study protocol, raw data, or programming code.	11

Supplementary Information S2. Details of the implementation of cohort study design

Identification of the exposure groups

In the main analysis, the exposure was defined as receipt of a DOAC (defined as the object drug) with a precipitant drug (DOAC + atorvastatin/simvastatin) and was compared with receipt of a DOAC and an active comparator (DOAC + other statins). For each drug (DOAC/precipitant of interest), the exposure start dates were identified using prescription start date from CPRD Aurum. We then estimated their prescription duration according to the recorded duration, or by calculation using quantity, dosage, and frequency. If there was any missingness or extreme values (based on the recommended treatment duration, or a maximum of 3 months for a prescription refill for ongoing therapies), we imputed the duration with the study population median for that drug. We assumed the treatment discontinued when there was a treatment gap of >1 day between each prescription. The exposure groups were then defined as overlapped person-time when a DOAC and statins were prescribed concurrently. The subsequent person-times of concomitant exposure to DOAC and statin were not included.

For people who were eligible to be both exposed and comparison group, whether they were classified as exposed or comparison group depending on the gaps between start dates of the concomitant exposure to atorvastatin/simvastatin and DOAC and that to DOAC + other statins (Figure S1). If a person was eligible to be included in the exposed group first (Patient 3 in Figure S1), and the treatment gap between exposure groups was more than 90 days, the person-times for exposed and comparison groups were included respectively. However, if the treatment gap was fewer or equal to 90 days, only the person-times for exposed group were included. As there was no drug interaction between DOAC and other statins, if a person was eligible to be included in the comparison group first, the person-times for exposed and comparison groups were included respectively regardless of the length of treatment gaps (Patient 4 in Figure S1). If a person started the DOAC, atorvastatin/simvastatin, and other statins on the same day, this person was eligible to be included in the DOAC+ atorvastatin/simvastatin group only (Patient 5 in Figure S1).

In the sensitivity analysis, the exposure was defined as receipt of a DOAC (defined as the object drug) with atorvastatin/simvastatin (concomitant group) and was compared with receipt of DOAC alone. The person-times of concomitant exposure to DOAC and atorvastatin/simvastatin after stopping the concomitant treatment were not included. People who ever had a warfarin prescription before their cohort entry were excluded to remove a carry-over effect of warfarin.

Outcomes

Effectiveness outcomes included ischaemic stroke, myocardial infarction, venous thromboembolism, cardiovascular mortality and all-cause mortality during the follow-up. Safety outcomes were intracranial bleeding, gastrointestinal bleeding and other bleeding.
We identified the first recorded event using International Classification of Diseases, Tenth Revision (ICD-10) codes in Hospital Episode Statistics (HES) and/or Office for National Statistics (ONS) data only to capture incident events in a cohort of people with a possible history of the outcome. The codelists to identify outcomes can be found in LSHTM Data Compass: https://doi.org/10.17037/DATA.00004214

Accuracy of diagnostic records of hospital episode data in Great Britain was high (positive predictive value: 80%).[1] Death registration data from ONS was based on the death certificate for the population of England and Wales and the accuracy of coding is regularly assessed.[2]

Covariates

The following covariates that are potential confounders or predictors of outcomes[3] were selected for different outcomes identified by directed acyclic diagrams from Supplementary Figures S3-5: lifestyle factors (smoking status and alcohol consumption), body mass index, deprivation status, region, age (categorised as 18-65, 65-75, 75+, same as the age component in CHA_2DS_2-VASc Score), sex, co-morbidities (chronic obstructive pulmonary disease, chronic renal disease, heart failure, ischaemic heart disease, ischaemic stroke/transient ischaemic attack, severity of diabetes mellitus (using use of insulin to derive disease severity), peripheral arterial disease, venous thromboembolism, bleeding, peptic ulcer), measurements of systolic and diastolic blood pressure in the past year, prescriptions used in the past 3 months (proton pump inhibitors, aspirin, antiplatelets, antidepressants, anticonvulsants, angiotensin-converting enzyme inhibitors, angiotensin receptor blockers, amiodarone, calcium channel blockers, beta-blockers, non-steroidal anti-inflammatory drugs, oral corticosteroids, macrolides, oestrogen/oestrogen like drugs), number of GP active consultation in the past year. Smoking status, body mass index and alcohol consumption were pragmatically based on status recorded closest to the first day of follow-up. Records within -1 year to +1 month from the first day of follow-up were regarded as the best, +1 months to +1 years from the first day of follow-up being second best, the nearest before -1 year from the first day of follow-up as the third best, and within +1 year from the first day of follow-up being least best. Severity of diabetes were defined as 3-level including no diabetes (i.e. without diabetes diagnosis nor insulin prescription); having a diabetes diagnosis only; diabetes diagnosis with an insulin prescription in the past 3 months/only receiving insulin prescription in the past 3 months but without a diabetes diagnosis. We used SNOMED CT (UK edition), Read Version and local EMIS Web® codes in CPRD Aurum, ICD-10 codes in HES to identify covariates as we wanted to capture complete record of prevalent disease. The drug prescriptions was coded using the Dictionary of Medicines and Devices (dm+d), which exists within the SNOMED CT terminological structure. We therefore used dm+d to identify drug prescription in the CPRD Aurum. The codelists to identify co-morbidities covariates can be found in LSHTM Data Compass: https://doi.org/10.17037/DATA.00004214

Statistical analyses

To reduce bias due to heterogeneity between the exposed and comparison group, propensity scores (PS) were used to re-weight the sample and achieve balance between groups on observed covariates. The PS is the probability of a patient receiving a certain treatment, based on the distribution of confounders among patients.[4] We derived PS from logistic regression, to represent the probability of exposure given the covariates measured on the first day of follow-up in each group. Weights are calculated as the inverse of PS for the exposed group and the inverse of (1-PS) for the comparison group and the balance of covariate distribution was assessed after weighting by calculating the standardised difference for each covariate. We performed multiple imputation through chained equations with 10 imputed datasets to address the missingness in blood pressure measurements, body mass index, smoking status, alcohol consumption and region. We estimated the treatment effect from each imputed dataset and combined them using Rubin's rules. As we used multiple imputation to address missing data in generating PS, we presented the maximum absolute values of standardised difference among imputed datasets. We restricted the cohort to those individuals whose PS were within the overlapping region of the distributions of the DOAC + atorvastatin/simvastatin group and the comparison group. Taking all-cause mortality as an example, at maximum only 70 people (0.09%) were excluded due to their PS were not within the overlapping region of the DOAC + other statins group and DOAC + atorvastatin group. At maximum 8 people (0.02%) were excluded due to their PS were not within the overlapping region of DOAC + other statins group and DOAC + simvastatin group. We computed the hazard ratios of the association using inverse probability of treatment-weighted Cox regressions. We computed the hazard ratios of the association using inverse probability of treatment-weighted Cox regressions.

Reference

1. Burns EM, Rigby E, Mamidanna R, et al. Systematic review of discharge coding accuracy. *J Public Health (Oxf)* 2012;34(1):138-48.
2. Medicines & Healthcare products Regulatory Agency. ONS death registration data and CPRD primary care data Documentation 2021 [Available from: https://cprd.com/sites/default/files/2022-02/Documentation_Death_set22_v2.6.pdf accessed 1 September 2023.
3. Brookhart MA, Schneeweiss S, Rothman KJ, Glynn RJ, Avorn J, Stürmer T. Variable selection for propensity score models. Am J Epidemiol. 2006 Jun 15;163(12):1149-56.
4. Sturmer T, Wyss R, Glynn RJ, Brookhart MA. Propensity scores for confounder adjustment when assessing the effects of medical interventions using nonexperimental study designs. J Intern Med 2014;275(6):570-80.

Figure S3. Directed acyclic diagram to select propensity score covariates for bleeding outcomes.

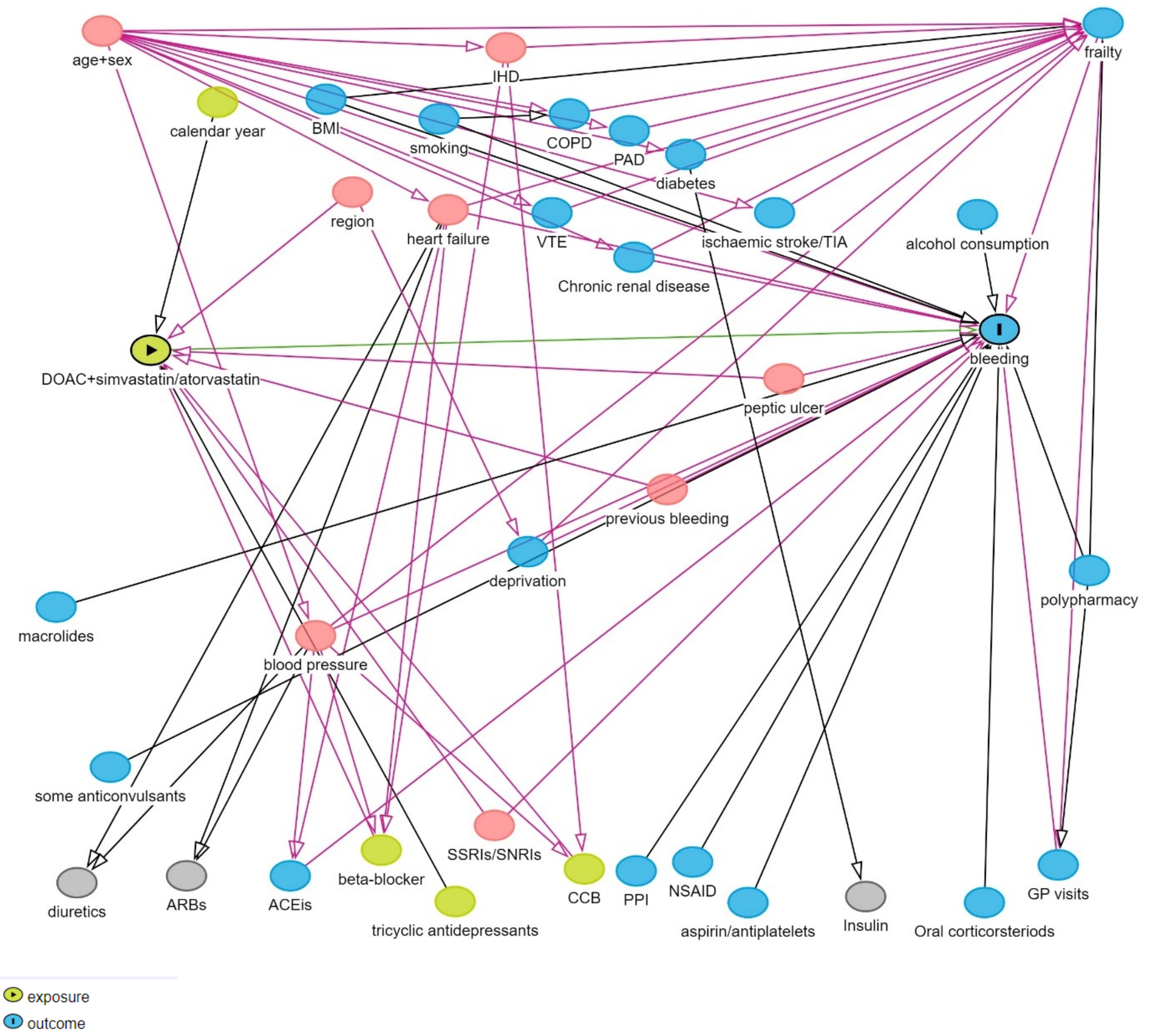

Colour indication: Green: predictor of exposure; blue: predictor of outcome; red: predictor of exposure and outcome (confounders); grey: other variables

Abbreviation: BMI, body mass index; IHD, ischaemic heart disease; COPD, chronic obstructive pulmonary disease; PAD, peripheral arterial disease; TIA, transient ischaemic attack; VTE, venous thromboembolism; ARB, angiotensin receptor blockers; ACEis, angiotensin-converting enzyme inhibitors; SSRI, selective serotonin reuptake inhibitors; SNRI, serotonin and norepinephrine reuptake inhibitors; CCB, calcium channel blockers; PPI, proton pump inhibitors; NSAID, non-steroid anti-inflammatory drugs; GP, general practitioners.

*Body mass index and ischaemic heart disease are associated with getting the exposure as they are indications of statins in sensitivity analysis (comparing DOAC+atorvastatin/simvastatin vs DOAC alone) and become a confounder, but as predictor of outcome were included as propensity score covariates so the selection of covariates would be the same as main and sensitivity analyses

Figure S4. Directed acyclic diagram to select propensity score covariates for cardiovascular outcomes.

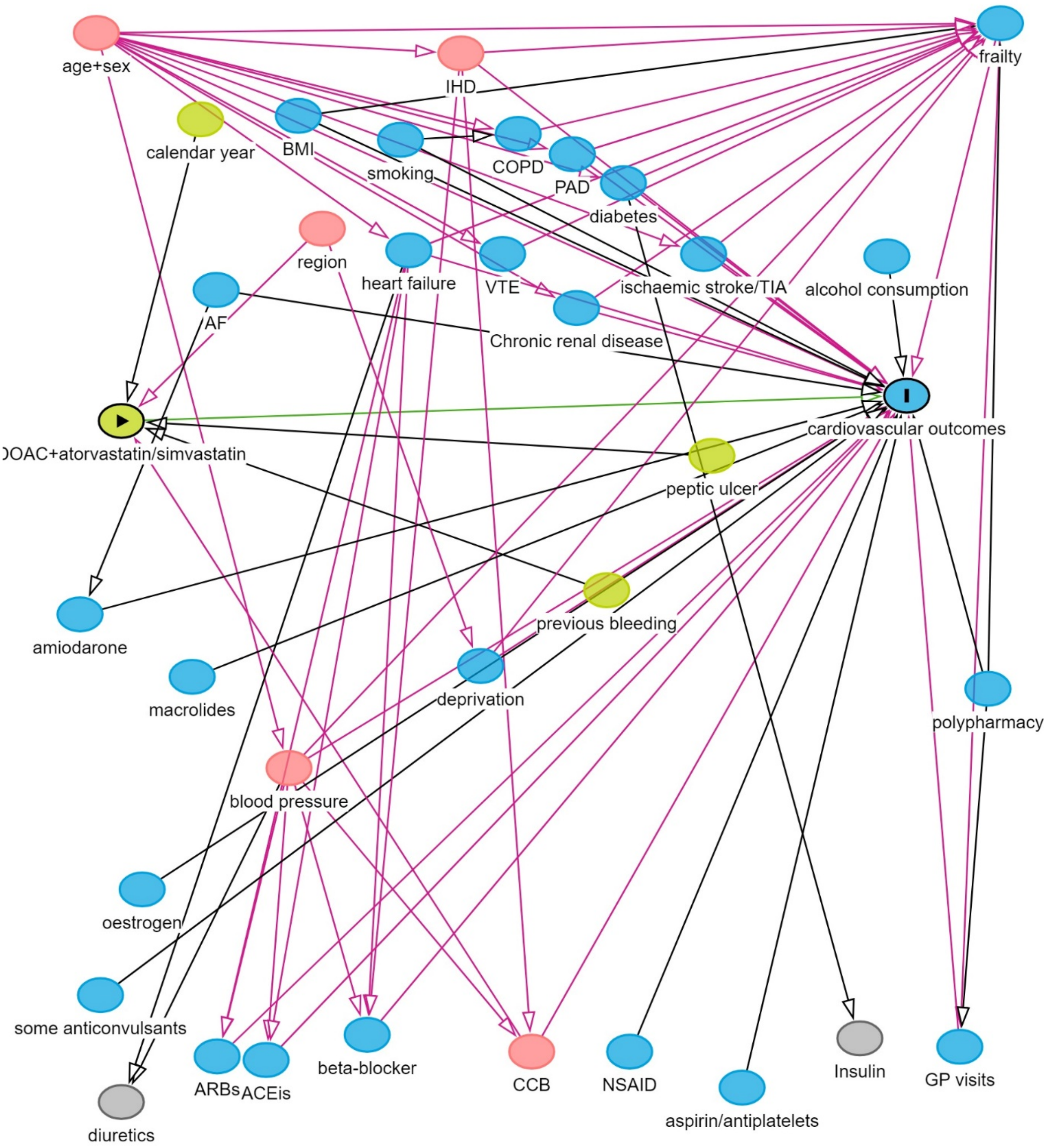

Colour indication: Green: predictor of exposure; blue: predictor of outcome; red: predictor of exposure and outcome (confounders); grey: other variables

Abbreviation: BMI, body mass index; IHD, ischaemic heart disease; COPD, chronic obstructive pulmonary disease; PAD, peripheral arterial disease; TIA, transient ischaemic attack; VTE, venous thromboembolism; ARB, angiotensin receptor blockers; ACEis, angiotensin-converting enzyme inhibitors; SSRI, selective serotonin reuptake inhibitors; SNRI, serotonin and norepinephrine reuptake inhibitors; CCB, calcium channel blockers; PPI, proton pump inhibitors; NSAID, non-steroid anti-inflammatory drugs; GP, general practitioners.

*Body mass index and ischaemic heart disease are associated with getting the exposure as they are indications of statins in sensitivity analysis (comparing DOAC+atorvastatin/simvastatin vs DOAC alone) and become a confounder, but as predictor of outcome were included as propensity score covariates so the selection of covariates would be the same as main and sensitivity analyses

Figure S5. Directed acyclic diagram to select propensity score covariates for all-cause mortality.

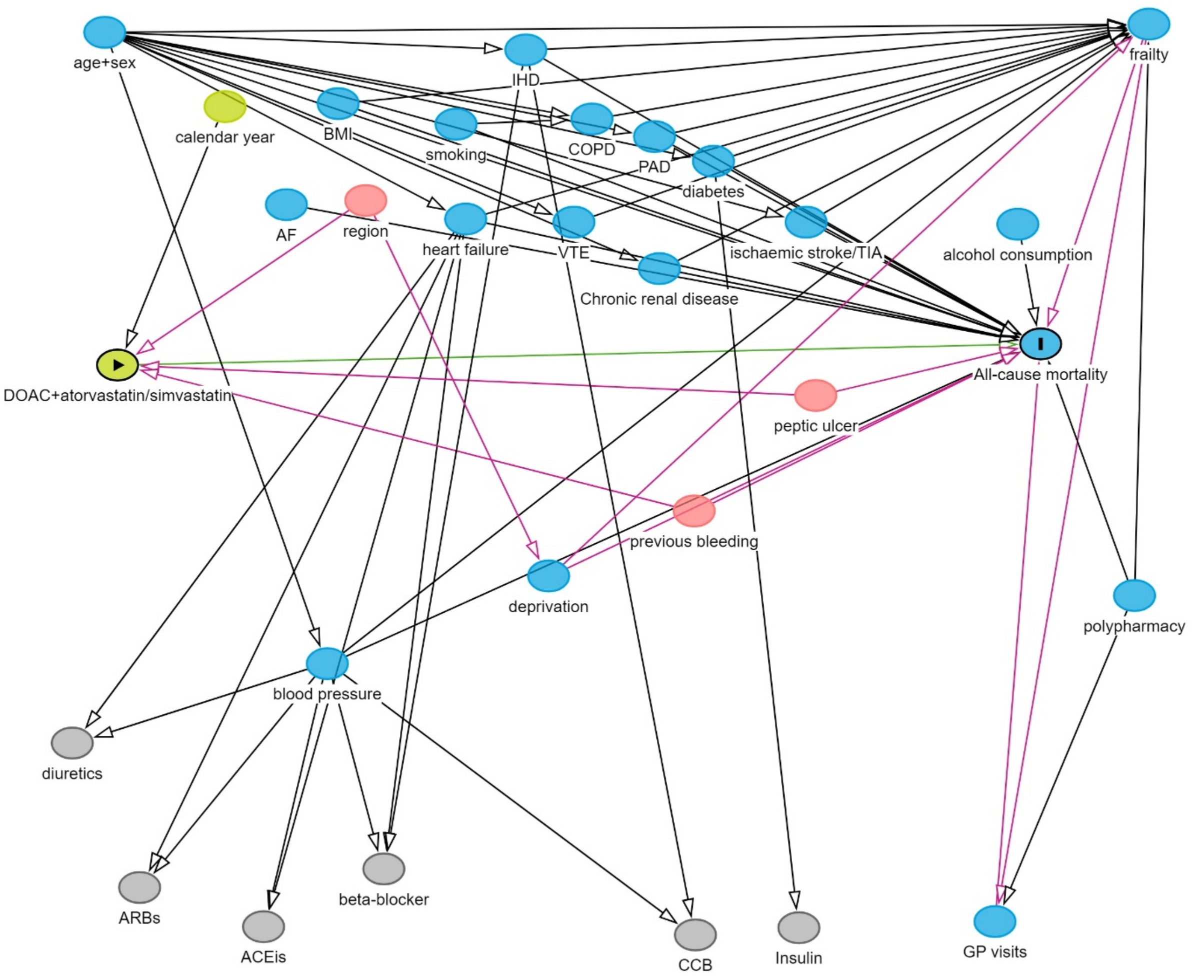

Colour indication: Green: predictor of exposure; blue: predictor of outcome; red: predictor of exposure and outcome (confounders); grey: other variables

Abbreviation: BMI, body mass index; IHD, ischaemic heart disease; COPD, chronic obstructive pulmonary disease; PAD, peripheral arterial disease; TIA, transient ischaemic attack; VTE, venous thromboembolism; ARB, angiotensin receptor blockers; ACEis, angiotensin-converting enzyme inhibitors; SSRI, selective serotonin reuptake inhibitors; SNRI, serotonin and norepinephrine reuptake inhibitors; CCB, calcium channel blockers; PPI, proton pump inhibitors; NSAID, non-steroid anti-inflammatory drugs; GP, general practitioners.

*Body mass index and ischaemic heart disease are associated with getting the exposure as they are indications of statins in sensitivity analysis (comparing DOAC+atorvastatin/simvastatin vs DOAC alone) and become a confounder, but as predictor of outcome were included as propensity score covariates so the selection of covariates would be the same as main and sensitivity analyses.

Supplementary Information S3. Illustration of drug initiation patterns in case-crossover study design using 6-parameter model

Strata	Strata description	Hazard window		Referent window		Odds ratio (99% CI)
		DOAC	statins	DOAC	statins	
1	Object drug (i.e. DOAC) only	1	0	0	0	
		0	0	1	0	Reference
2	Precipitant drug (i.e. statins) only	0	1	0	0	
		0	0	0	1	Reference
3	Joint exposure	1	1	0	0	
		0	0	1	1	Reference
4	Object drug while on precipitant drug	1	1	0	1	
		0	1	1	1	Reference
5	Precipitant drug while on object drug	1	1	1	0	
		1	0	1	1	Reference
6	Switch	1	0	0	1	
		0	1	1	0	Reference

Abbreviation: DOAC, direct oral anticoagulant

Outcomes

Effectiveness outcomes included ischaemic stroke, myocardial infarction (MI), venous thromboembolism (VTE), cardiovascular mortality and all-cause mortality during the follow-up. Safety outcomes were intracranial bleeding, gastrointestinal bleeding and other bleeding. We defined outcome as first hospital and/or death record for ischaemic stroke, MI, VTE, intracranial bleeding, gastrointestinal bleeding, other bleeding and cardiovascular mortality. All-cause mortality was identified using CPRD primary care records only during the study period.

Supplementary Information S4. Description of interpretations for case-crossover design

In the 6-parameter model, the first 3 parameters address situations where a drug interaction could not have occurred, namely 1) use of one drug in the hazard window and the other drug in the control window, 2) initiation of DOAC monotherapy, 3) initiation of statins monotherapy; the remaining 3 parameters address situations related to potential drug-interaction: 4) joint initiation, 5) initiation of DOAC while taking statins, 6) initiation of statins while taking DOAC.

The following combinations of parameters should be considered:

Combination	Parameter 1	Parameter 2
A	Initiation of precipitant drug in the presence of DOAC	Initiation of precipitant drug monotherapy
B	Joint initiation of precipitant drug and DOAC	Initiation of precipitant drug monotherapy<br>OR<br>Initiation of DOAC monotherapy
C	Initiation of DOAC in the presence of precipitant drug	Initiation of DOAC monotherapy

*DOAC, direct oral anticoagulant

To identify potential increased risk of an outcome due to drug-drug interaction:

In each of these combinations, if the odds ratio (OR) derived from Parameter 1 is greater than that obtained from Parameter 2 and if a Wald test suggests evidence that the OR for parameter 1 was greater than the OR for parameter 2, this was considered to signify a potential drug-drug interaction.

Of note, if this pattern of results is only observed for Combination A, the drug-drug interaction could be due to poorly dose-titrated DOAC, e.g. if a prescriber was unaware to reduce the dose of the DOAC when initiating the precipitant drug to avoid a drug-drug interaction. Similarly, if this pattern of results is only observed for Combination C, the drug-drug interaction could reflect the dose of the precipitant drug not being adjusted when initiating DOAC to avoid a drug-drug interaction. If the pattern of results is observed for at least 2 of the combinations, this would suggest a drug-drug interaction.

To identify potential confounding:

If combination B shows an increased OR for initiating both drugs together, compared with initiation of either DOAC monotherapy/precipitant drug monotherapy (tested using a Wald test) AND, this is not observed in Combinations A and C, this could suggest confounding by indication, as the joint initiation of the two therapies may imply that multiple medical conditions requiring separate treatments were present at this point in time, and this multimorbidity rather than the drugs themselves could have driven poorer outcomes.

Figure S6. Flow diagram for inclusion in cohort study design

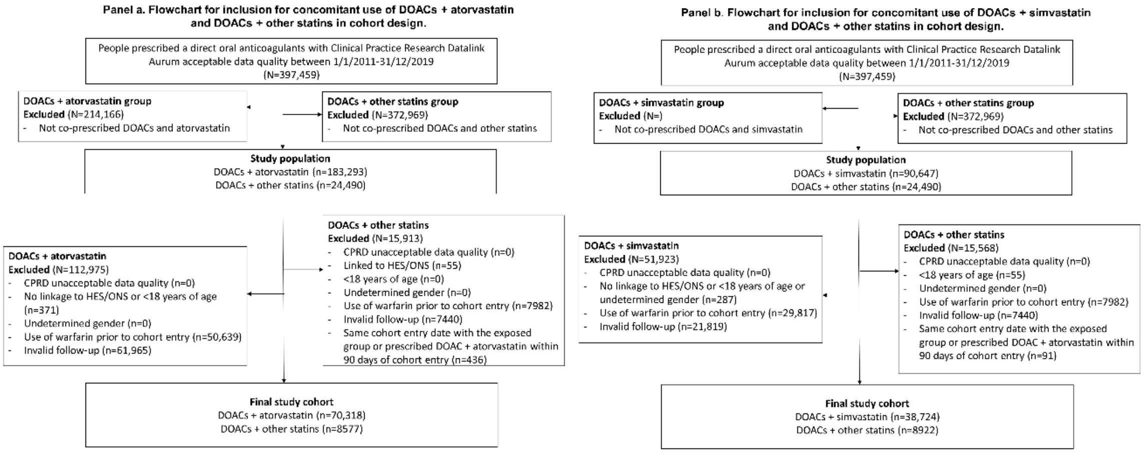

Table S1. Baseline characteristics of the DOAC + atorvastatin group and DOAC + other statins group before restricting people with overlapping propensity score distributions between groups

	DOAC + other statins	DOAC + atorvastatin
Total	8,577	70,318
Age at index date		
Median (IQR)	75.4 (69.4-81.4)	74.8 (67.7-81.5)
Min, Max	29.0, 101.1	18.6, 102.9
Age group		
18-<40	15 (0.2)	172 (0.2)
40-<50	82 (1.0)	1,186 (1.7)
50-<60	418 (4.9)	5,511 (7.8)
60-<70	1,813 (21.1)	15,729 (22.4)
70-<80	3,595 (41.9)	26,420 (37.6)
80+	2,654 (30.9)	21,300 (30.3)
Male sex	4,779 (55.7)	40,946 (58.2)
Calendar year at cohort entry		
2011	13 (0.2)	20 (0.0)
2012	82 (1.0)	301 (0.4)
2013	245 (2.9)	1,162 (1.7)
2014	506 (5.9)	2,909 (4.1)
2015	951 (11.1)	6,693 (9.5)
2016	1,322 (15.4)	10,569 (15.0)
2017	1,615 (18.8)	14,076 (20.0)
2018	1,770 (20.6)	16,455 (23.4)
2019	2,073 (24.2)	18,133 (25.8)
Body mass index		
Underweight	92 (1.1)	976 (1.4)
Normal	1,913 (22.3)	16,276 (23.1)
Overweight	3,277 (38.2)	25,345 (36.0)
Obese	3,235 (37.7)	27,022 (38.4)
Missing	60 (0.7)	699 (1.0)
Smoking status		
Non-smoker	1,617 (18.9)	13,932 (19.8)
Current smoker	1,750 (20.4)	15,826 (22.5)
Ex-smoker	5,204 (60.7)	40,504 (57.6)
Missing	6 (0.1)	56 (0.1)

Ethnicity		
White	8,192 (95.5)	66,178 (94.1)
South Asian	184 (2.1)	1,880 (2.7)
Black	80 (0.9)	1,118 (1.6)
Other	45 (0.5)	456 (0.6)
Mixed	28 (0.3)	238 (0.3)
Not stated	20 (0.2)	251 (0.4)
Missing	28 (0.3)	197 (0.3)
Index of Multiple Deprivation		
1 (least deprived)	2,174 (25.3)	15,737 (22.4)
2	2,091 (24.4)	15,352 (21.8)
3	1,763 (20.6)	13,736 (19.5)
4	1,386 (16.2)	13,142 (18.7)
5 (most deprived)	1,163 (13.6)	12,351 (17.6)
Alcohol status		
Non-drinker	606 (7.1)	5,620 (8.0)
Current low level	3,848 (44.9)	29,943 (42.6)
Current Medium level	1,071 (12.5)	8,144 (11.6)
Current high level	384 (4.5)	3,968 (5.6)
Current without consumption data	674 (7.9)	5,673 (8.1)
Ex-drinker	1,807 (21.1)	15,399 (21.9)
Missing	187 (2.2)	1,571 (2.2)
Systolic blood pressure *(in quartile)*		
Q1 (40-120 mmHg)	2,004 (23.4)	17,614 (25.0)
Q2 (121-131 mmHg)	2,078 (24.2)	16,227 (23.1)
Q3 (131.5-140 mmHg)	2,232 (26.0)	17,903 (25.5)
Q4 (141-235 mmHg)	1,876 (21.9)	15,131 (21.5)
Missing	387 (4.5)	3,443 (4.9)
Diastolic blood pressure *(in quartiles)*		
Q1 (30-69 mmHg)	2,034 (23.7)	16,757 (23.8)
Q2 (70-76 mmHg)	2,319 (27.0)	18,521 (26.3)
Q3 (77-81 mmHg)	1,996 (23.3)	15,627 (22.2)
Q4 (82-175 mmHg)	1,837 (21.4)	15,924 (22.6)
Missing	391 (4.6)	3,489 (5.0)
Region[a]		
North East	295 (3.4)	3,205 (4.6)

North West	1,674 (19.5)	13,090 (18.6)
Yorkshire & The Humber	359 (4.2)	2,715 (3.9)
East Midlands	192 (2.2)	1,505 (2.1)
West Midlands	1,301 (15.2)	11,435 (16.3)
East of England	436 (5.1)	3,120 (4.4)
London	882 (10.3)	8,620 (12.3)
South East	1,990 (23.2)	15,165 (21.6)
South West	1,448 (16.9)	11,460 (16.3)
Missing	0	<5
Polypharmacy (≥5 drugs)	7,798 (90.9)	62,625 (89.1)
Polypharmacy degree		
1-4 drugs	779 (9.1)	7,693 (10.9)
5-9 drugs	4,405 (51.4)	35,122 (49.9)
≥10 drugs	3,393 (39.6)	27,503 (39.1)
Medical history		
COPD	1,416 (16.5)	12,218 (17.4)
Heart failure	1,816 (21.2)	16,977 (24.1)
Ischaemic heart disease	4,208 (49.1)	32,508 (46.2)
Peptic ulcer	807 (9.4)	6,499 (9.2)
Diabetes		
Without insulin	2,466 (28.8)	20,449 (29.1)
With insulin	438 (5.1)	3,619 (5.1)
Peripheral arterial disease	832 (9.7)	6,545 (9.3)
Atrial fibrillation	6,523 (76.1)	54,200 (77.1)
Venous thromboembolism	1,392 (16.2)	10,816 (15.4)
Any bleeding	4,770 (55.6)	37,180 (52.9)
Stroke/TIA	1,643 (19.2)	16,511 (23.5)
Chronic kidney disease stage		
Stage 3a	1,380 (16.1)	10,188 (14.5)
Stage 3b	588 (6.9)	4,646 (6.6)
Stage 4	128 (1.5)	897 (1.3)
Stage 5	110 (1.3)	927 (1.3)
Missing	790 (9.2)	8,732 (12.4)
Medications used in the past 3 months		
PPIs	4,114 (48.0)	32,813 (46.7)
aspirin	3,016 (35.2)	23,820 (33.9)
antiplatelet	1,420 (16.6)	14,062 (20.0)
amiodarone	256 (3.0)	2,209 (3.1)
SSRI/SNRI	902 (10.5)	7,719 (11.0)

Anticonvulsant*	48 (0.6)	491 (0.7)
ACEIs	3,191 (37.2)	29,424 (41.8)
ARBs	2,088 (24.3)	12,403 (17.6)
CCBs	3,294 (38.4)	27,182 (38.7)
betablocker	5,013 (58.4)	43,223 (61.5)
NSAIDs	1,134 (13.2)	8,299 (11.8)
Oral corticosteroids	814 (9.5)	6,114 (8.7)
Macrolides	349 (4.1)	2,442 (3.5)
Oestrogen/oestrogen-like drugs	78 (0.9)	447 (0.6)
No of GP active consultation in the past year		
Median (IQR)	14 (9-21)	13 (8-21)
Min, Max	0, 283	0, 205
≥12 visits	5,424 (63.2)	41,130 (58.5)
<12 visits	3,117 (36.3)	28,909 (41.1)
none	36 (0.4)	279 (0.4)

Abbreviation: DOAC, direct oral anticoagulants; PPIs, proton pump inhibitors; IQR, interquartile range; COPD, chronic obstructive pulmonary disease; TIA, transient ischaemic attack; ACEI, angiotensin-converting enzyme inhibitors; ARBs, angiotensin receptor blockers; CCBs, calcium channel blockers; NSAIDs, non-steroidal anti-inflammatory drugs; SSRI, Selective serotonin reuptake inhibitor; SNRI, Serotonin and norepinephrine reuptake inhibitor; GP, general practice

*Anticonvulsants with side effect of bleeding

[a]Round to nearest 5 due to data redaction

Table S2. Baseline characteristics of the DOAC + simvastatin group and DOAC + other statins group before restricting people with overlapping propensity score distributions between groups

	DOAC + other statins	DOAC + simvastatin
Total	8,922	38,724
Age at index date		
Median (IQR)	75.3 (69.3-81.4)	77.9 (70.9-84.0)
Min, Max	29.0, 101.1	27.5, 103.3
Age group		
18-<40	16 (0.2)	42 (0.1)
40-<50	88 (1.0)	332 (0.9)
50-<60	449 (5.0)	1,713 (4.4)
60-<70	1,908 (21.4)	6,504 (16.8)
70-<80	3,739 (41.9)	14,188 (36.6)
80+	2,722 (30.5)	15,945 (41.2)
Male sex	4,968 (55.7)	21,775 (56.2)
Calendar year at cohort entry		
2011	12 (0.1)	97 (0.3)
2012	81 (0.9)	681 (1.8)
2013	247 (2.8)	1,803 (4.7)
2014	516 (5.8)	3,446 (8.9)
2015	981 (11.0)	5,713 (14.8)
2016	1,357 (15.2)	6,879 (17.8)
2017	1,679 (18.8)	6,976 (18.0)
2018	1,872 (21.0)	6,826 (17.6)
2019	2,177 (24.4)	6,303 (16.3)
Body mass index		
Underweight	96 (1.1)	645 (1.7)
Normal	1,970 (22.1)	9,415 (24.3)
Overweight	3,432 (38.5)	13,924 (36.0)
Obese	3,365 (37.7)	14,286 (36.9)
Missing	59 (0.7)	454 (1.2)
Smoking status		
Non-smoker	1,688 (18.9)	7,537 (19.5)
Current smoker	1,826 (20.5)	8,399 (21.7)
Ex-smoker	5,402 (60.5)	22,763 (58.8)
Missing	6 (0.1)	25 (0.1)

Ethnicity		
White	8,518 (95.5)	37,053 (95.7)
South Asian	193 (2.2)	756 (2.0)
Black	82 (0.9)	422 (1.1)
Other	51 (0.6)	169 (0.4)
Mixed	31 (0.3)	91 (0.2)
Not stated	22 (0.2)	121 (0.3)
Missing	25 (0.3)	112 (0.3)
Index of Multiple Deprivation		
1 (least deprived)	2,283 (25.6)	8,654 (22.3)
2	2,167 (24.3)	8,361 (21.6)
3	1,813 (20.3)	7,742 (20.0)
4	1,455 (16.3)	7,108 (18.4)
5 (most deprived)	1,204 (13.5)	6,859 (17.7)
Alcohol status		
Non-drinker	630 (7.1)	3,240 (8.4)
Current low level	4,007 (44.9)	16,857 (43.5)
Current Medium level	1,128 (12.6)	3,834 (9.9)
Current high level	402 (4.5)	1,655 (4.3)
Current without consumption data	693 (7.8)	3,439 (8.9)
Ex-drinker	1,868 (20.9)	8,820 (22.8)
Missing	194 (2.2)	879 (2.3)
Systolic blood pressure *(in quartile)*		
Q1 (54- 120 mmHg)	2,107 (23.6)	9,787 (25.3)
Q2 (121-131 mmHg)	2,141 (24.0)	9,060 (23.4)
Q3 (132-140 mmHg)	2,343 (26.3)	9,910 (25.6)
Q4 (141-238 mmHg)	1,932 (21.7)	8,125 (21.0)
Missing	399 (4.5)	1,842 (4.8)
Diastolic blood pressure *(in quartiles)*		
Q1 (30-69 mmHg)	2,106 (23.6)	9,574 (24.7)
Q2 (70-75 mmHg)	2,085 (23.4)	9,339 (24.1)
Q3 (76-80 mmHg)	2,280 (25.6)	9,573 (24.7)
Q4 (81-175 mmHg)	2,048 (23.0)	8,370 (21.6)
Missing	403 (4.5)	1,868 (4.8)
Region[a]		
North East	305 (3.4)	1,505 (3.9)
North West	1,724 (19.3)	7,410 (19.1)

Yorkshire & The Humber	374 (4.2)	1,400 (3.6)
East Midlands	196 (2.2)	935 (2.4)
West Midlands	1,358 (15.2)	6,500 (16.8)
East of England	458 (5.1)	1,900 (4.9)
London	912 (10.2)	3,745 (9.7)
South East	2,093 (23.5)	8,470 (21.9)
South West	1,502 (16.8)	6,865 (17.7)
Missing	0	<5
Polypharmacy (≥5 drugs)	8,096 (90.7)	35,370 (91.3)
Polypharmacy degree		
1-4 drugs	826 (9.3)	3,354 (8.7)
5-9 drugs	4,586 (51.4)	19,774 (51.1)
≥10 drugs	3,510 (39.3)	15,596 (40.3)
Medical history		
COPD	1,481 (16.6)	6,827 (17.6)
Heart failure	1,877 (21.0)	8,041 (20.8)
Ischaemic heart disease	4,375 (49.0)	15,182 (39.2)
Peptic ulcer	836 (9.4)	3,750 (9.7)
Diabetes		
Without insulin	2,537 (28.4)	11,161 (28.8)
With insulin	454 (5.1)	1,751 (4.5)
Peripheral arterial disease	863 (9.7)	3,108 (8.0)
Atrial fibrillation	6,805 (76.3)	29,191 (75.4)
Venous thromboembolism	1,431 (16.0)	6,287 (16.2)
Any bleeding	4,978 (55.8)	20,572 (53.1)
Stroke/TIA	1,736 (19.5)	7,694 (19.9)
Chronic kidney disease		
Stage 3a	1,420 (15.9)	6,407 (16.5)
Stage 3b	604 (6.8)	3,090 (8.0)
Stage 4	131 (1.5)	572 (1.5)
Stage 5	112 (1.3)	394 (1.0)
Missing	829 (9.3)	4,890 (12.6)
Medications used in the past 3 months		
PPIs	4,289 (48.1)	17,033 (44.0)
aspirin	3,137 (35.2)	15,058 (38.9)
antiplatelet	1,508 (16.9)	5,276 (13.6)
amiodarone	272 (3.0)	685 (1.8)
SSRI/SNRI	928 (10.4)	4,127 (10.7)
Anticonvulsant*	48 (0.5)	328 (0.8)

ACEIs	3,319 (37.2)	16,142 (41.7)
ARBs	2,163 (24.2)	6,729 (17.4)
CCBs	3,423 (38.4)	12,782 (33.0)
betablocker	5,226 (58.6)	22,154 (57.2)
NSAIDs	1,151 (12.9)	5,174 (13.4)
Oral corticosteroids	850 (9.5)	3,456 (8.9)
Macrolides	364 (4.1)	1,451 (3.7)
Oestrogen/oestrogen like drugs	80 (0.9)	196 (0.5)
No of GP active consultation in the past year		
Median (IQR)	14 (9-21)	13 (8-21)
Min, Max	0, 283	0, 311
≥12 visits	5,646 (63.3)	22,696 (58.6)
<12 visits	3,240 (36.3)	15,905 (41.1)
none	36 (0.4)	123 (0.3)

Abbreviation: DOAC, direct oral anticoagulants; PPIs, proton pump inhibitors; IQR, interquartile range; COPD, chronic obstructive pulmonary disease; TIA, transient ischaemic attack; ACEI, angiotensin-converting enzyme inhibitors; ARBs, angiotensin receptor blockers; CCBs, calcium channel blockers; NSAIDs, non-steroidal anti-inflammatory drugs; SSRI, Selective serotonin reuptake inhibitor; SNRI, Serotonin and norepinephrine reuptake inhibitor; GP, general practice

*Anticonvulsants with side effect of bleeding

[a]Round to nearest 5 due to data redaction

Table S3. Standardised differences for the associations between concomitant use of DOACs and atorvastatin and bleeding outcomes using active comparator in cohort study

Outcomes	Intracranial bleeding	Gastrointestinal bleeding	Other bleeding
Age group			
18-<40	1 (ref)	1 (ref)	1 (ref)
40-<50	0.006223	0.006224	0.006221
50-<60	0.021751	0.021753	0.021753
60-<70	0.046532	0.046534	0.046539
70-<80	0.006012	0.006016	0.006013
80+	0.033481	0.033475	0.03348
Male sex	0.007524	0.007532	0.007529
Body mass index			
Underweight	1 (ref)	1 (ref)	1 (ref)
Normal	0.002084	0.002051	0.002084
Overweight	0.004962	0.004975	0.00497
Obese	0.006134	0.006132	0.006131
Smoking status			
Non-smoker	1 (ref)	1 (ref)	1 (ref)
Current smoker	0.008343	0.008362	0.008357
Ex-smoker	0.006416	0.00642	0.006413
Index of Multiple Deprivation			
1 (least deprived)	1 (ref)	1 (ref)	1 (ref)
2	0.003469	0.003479	0.003469
3	0.004019	0.004012	0.004018
4	0.003276	0.003274	0.003279
5 (most deprived)	0.004001	0.004004	0.003996
Alcohol consumption			
Non-drinker	1 (ref)	1 (ref)	1 (ref)
Current low level	0.002988	0.002974	0.003022
Current Medium level	0.004515	0.004513	0.004647
Current high level	0.006114	0.006153	0.006161
Ex-drinker	0.003052	0.003044	0.003041
Current drinker with missing data on consumption level	0.007993	0.007998	0.007989
Systolic blood pressure *(in quartile)*			
Q1 (40-120 mmHg)	1 (ref)	1 (ref)	1 (ref)
Q2 (121-131 mmHg)	0.008853	0.008863	0.008952
Q3 (131.5-140 mmHg)	0.0048	0.004832	0.004878

Q4 (141-235 mmHg)	0.00497	0.005038	0.005068
Diastolic blood pressure *(in quartiles)*			
Q1 (30-69 mmHg)	1 (ref)	1 (ref)	1 (ref)
Q2 (70-76 mmHg)	0.007676	0.007727	0.007746
Q3 (77-81 mmHg)	0.005538	0.005581	0.005573
Q4 (82-175 mmHg)	0.008384	0.008368	0.008393
Region			
North East	1 (ref)	1 (ref)	1 (ref)
North West	0.002256	0.002265	0.002257
Yorkshire & The Humber	0.00208	0.002086	0.002081
East Midlands	0.00032	0.000319	0.000323
West Midlands	0.001226	0.001229	0.001227
East of England	0.003573	0.003576	0.003578
London	0.004948	0.004938	0.004946
South East	0.006043	0.006044	0.006047
South West	0.00053	0.00053	0.000521
Polypharmacy (≥5 drugs)	0.005187	0.005191	0.005188
Medical history			
COPD	0.002309	0.002296	0.002293
Heart failure	0.005192	0.00521	0.005202
Ischaemic heart disease	0.002415	0.002408	0.002412
Peptic ulcer	0.003486	0.003485	0.003489
Diabetes			
without insulin	0.004454	0.004446	0.004449
with insulin	0.006048	0.006046	0.006046
Peripheral arterial disease	0.001191	0.001201	0.001193
Venous thromboembolism	0.006884	0.006883	0.006883
Any bleeding	0.001669	0.00166	0.001666
Stroke/TIA	0.005769	0.005764	0.005724
Chronic kidney disease			
Stage 3a	0.008833	0.008833	0.008834
Stage 3b	0.00979	0.00979	0.009788
Stage 4	0.014599	0.014605	0.014598
Stage 5	0.010851	0.010849	0.010849
Medication use in the past 3 months			
PPIs	0.002653	0.002647	0.002642
aspirin	0.001791	0.001802	0.001803
antiplatelet	0.004445	0.004448	0.004448
SSRI/SNRI	0.001199	0.0012	0.001204

anticonvulsant	0.003563	0.003565	0.003566
ACEI	0.002787	0.002795	0.002773
NSAIDs	0.00085	0.000852	0.000853
Oral corticosteroids	0.00247	0.002472	0.002602
Macrolides	0.004887	0.004882	0.004879
No of GP active consultation in the past year			
≥12 visits	1 (ref)	1 (ref)	1 (ref)
<12 visits	0.002507	0.002495	0.002498
none	0.007912	0.007906	0.007917

Abbreviation: NA, not applicable; DOAC, direct oral anticoagulants; PPIs, proton pump inhibitors; SSRI/SNRI, SSRI, selective serotonin reuptake inhibitors/serotonin and norepinephrine reuptake inhibitors; IQR, interquartile range; COPD, chronic obstructive pulmonary disease; TIA, transient ischaemic attack; ACEI, angiotensin-converting enzyme inhibitors; CCBs, calcium channel blockers; NSAIDs, non-steroidal anti-inflammatory drugs; GP, general practice

Table S4. Standardised differences for the associations between concomitant use of DOACs and atorvastatin and cardiovascular outcomes using active comparator in cohort study

	Ischaemic stroke	Myocardial infarction	Venous thromboembolism	Cardiovascular mortality
Age group				
18-<40	1 (ref)	1 (ref)	1 (ref)	1 (ref)
40-<50	0.005471	0.005468	0.005167	0.005458
50-<60	0.023374	0.023372	0.023817	0.023371
60-<70	0.049263	0.049259	0.049515	0.049254
70-<80	0.005314	0.005318	0.005069	0.005305
80+	0.031734	0.031721	0.031852	0.031708
Male sex	0.009006	0.009002	0.009064	0.00903
Body mass index				
Underweight	1 (ref)	1 (ref)	1 (ref)	1 (ref)
Normal	0.003332	0.003304	0.004089	0.003314
Overweight	0.006029	0.006008	0.005592	0.006172
Obese	0.006581	0.006545	0.006239	0.00648
Smoking status				
Non-smoker	1 (ref)	1 (ref)	1 (ref)	1 (ref)
Current smoker	0.009566	0.009552	0.00935	0.009551
Ex-smoker	0.009264	0.009263	0.009574	0.00922
Index of Multiple Deprivation				
1 (least deprived)	1 (ref)	1 (ref)	1 (ref)	1 (ref)
2	0.002036	0.002043	0.002134	0.002052
3	0.004898	0.004903	0.00476	0.004896
4	0.002247	0.002237	0.002145	0.002252
5 (most deprived)	0.004042	0.004049	0.004062	0.004035
Alcohol consumption				
Non-drinker	1 (ref)	1 (ref)	1 (ref)	1 (ref)
Current low level	0.001944	0.001945	0.002808	0.001981
Current Medium level	0.004541	0.004554	0.00467	0.004468
Current high level	0.010242	0.010246	0.009414	0.010235
Ex-drinker	0.006711	0.006711	0.010344	0.006697
Current drinker with missing data on consumption level	0.007819	0.007849	0.008503	0.007869
Systolic blood pressure *(in quartile)*				
Q1 (40-120 mmHg)	1 (ref)	1 (ref)	1 (ref)	1 (ref)
Q2 (121-131 mmHg)	0.007957	0.007941	0.007257	0.007596

Q3 (131.5-140 mmHg)	0.005912	0.005933	0.00433	0.006018
Q4 (141-235 mmHg)	0.005674	0.005672	0.007074	0.005704
Diastolic blood pressure *(in quartiles)*				
Q1 (30-69 mmHg)	1 (ref)	1 (ref)	1 (ref)	1 (ref)
Q2 (70-76 mmHg)	0.005609	0.0055	0.002143	0.005575
Q3 (77-81 mmHg)	0.005239	0.005252	0.004758	0.005204
Q4 (82-175 mmHg)	0.008269	0.008233	0.00718	0.008252
Region				
North East	1 (ref)	1 (ref)	1 (ref)	1 (ref)
North West	0.001943	0.001941	0.001749	0.001936
Yorkshire & The Humber	0.003677	0.003668	0.003698	0.003673
East Midlands	0.002462	0.002461	0.002355	0.00247
West Midlands	0.002104	0.002098	0.002181	0.0021
East of England	0.00436	0.004361	0.004324	0.004367
London	0.004578	0.004579	0.004647	0.004537
South East	0.006429	0.006437	0.006629	0.006437
South West	0.000986	0.000985	0.000913	0.001003
Polypharmacy (≥5 drugs)	0.010503	0.010502	0.010491	0.010517
Medical history				
COPD	0.002458	0.00246	0.002431	0.002461
Heart failure	0.004669	0.004663	0.00451	0.004644
Ischaemic heart disease	0.000287	0.000278	0.000236	0.000262
Diabetes				
without insulin	0.006923	0.006928	0.007098	0.006912
with insulin	0.006928	0.00693	0.00694	0.006921
Peripheral arterial disease	0.002515	0.002514	0.002466	0.002496
Atrial fibrillation	0.006243	0.006233	0.006263	0.006245
Venous thromboembolism	0.005714	0.005711	0.005626	0.005728
Stroke/TIA	0.003272	0.003285	0.003311	0.003263
Chronic kidney disease				
Stage 3a	0.007701	0.007697	0.007921	0.007704
Stage 3b	0.009372	0.009368	0.009255	0.009368
Stage 4	0.017271	0.017263	0.017438	0.017263
Stage 5	0.006166	0.006161	0.006265	0.006159
Medication use in the past 3 months				
diazepam	0.012089	0.012082	0.002649	0.012066
aspirin	0.001259	0.001258	0.00149	0.001257

antiplatelet	0.004651	0.004664	0.004667	0.004639
amiodarone	0.004279	0.004277	0.004264	0.004319
ACEIs	0.001984	0.001984	0.001984	0.001995
ARBs	0.007513	0.00751	0.007698	0.007517
CCBs	0.01049	0.010486	0.010414	0.010512
Beta blockers	0.007034	0.007031	0.007271	0.007054
NSAIDs	0.00124	0.001238	0.001192	0.001231
Macrolides	0.006851	0.006847	0.006937	0.006844
Oestrogen/oestrogen like drugs	0.004382	0.004383	0.004602	0.004388
No of GP active consultation in the past year				
≥12 visits	1 (ref)	1 (ref)	1 (ref)	1 (ref)
<12 visits	0.005343	0.005335	0.005485	0.005324
none	0.007957	0.007957	0.008068	0.007957

Abbreviation: DOAC, direct oral anticoagulants; IQR, interquartile range; COPD, chronic obstructive pulmonary disease; TIA, transient ischaemic attack; ACEI, angiotensin-converting enzyme inhibitors; ARB, angiotensin receptor blockers; CCBs, calcium channel blockers; NSAIDs, non-steroidal anti-inflammatory drugs; GP, general practice

Table S5. Standardised differences for the associations between concomitant use of DOACs and atorvastatin/simvastatin and all-cause mortality using active comparator in cohort study

	atorvastatin		simvastatin
Age group		Age group	
18-<40	1 (ref)	18-<40	1 (ref)
40-<50	0.008724	40-<50	0.024525
50-<60	0.020123	50-<60	0.006796
60-<70	0.044956	60-<70	0.017167
70-<80	0.008406	70-<80	0.004912
80+	0.030109	80+	**0.103571**
Male sex	0.003026	Male sex	0.003083
Body mass index		Body mass index	
Underweight	1 (ref)	Underweight	1 (ref)
Normal	0.002409	Normal	0.005507
Overweight	0.005021	Overweight	0.00498
Obese	0.005485	Obese	0.011748
Smoking status		Smoking status	
Non-smoker	1 (ref)	Non-smoker	1 (ref)
Current smoker	0.006719	Current smoker	0.005187
Ex-smoker	0.006493	Ex-smoker	0.002694
Index of Multiple Deprivation		Index of Multiple Deprivation	
1 (least deprived)	1 (ref)	1 (least deprived)	1 (ref)
2	0.001474	2	0.000795
3	0.002512	3	0.000582
4	0.002537	4	0.000438
5 (most deprived)	0.00231	5 (most deprived)	0.002933
Alcohol consumption		Alcohol consumption	
Non-drinker	1 (ref)	Non-drinker	1 (ref)
Current low level	0.002939	Current low level	0.006033
Current Medium level	0.006622	Current Medium level	0.003579
Current high level	0.00491	Current high level	0.005673
Ex-drinker	0.003716	Ex-drinker	0.005429
Current drinker with missing data on consumption level	0.006691	Current drinker with missing data on consumption level	0.002498
Systolic blood pressure *(in quartile)*		Systolic blood pressure *(in quartile)*	
Q1 (40-120 mmHg)	1 (ref)	Q1 (54-120 mmHg)	1 (ref)

Q2 (121-131 mmHg)	0.006005	Q2 (121-131 mmHg)	0.003894
Q3 (131.5-140 mmHg)	0.005854	Q3 (132-140 mmHg)	0.009014
Q4 (141-235 mmHg)	0.007813	Q4 (141-238 mmHg)	0.005711
Diastolic blood pressure *(in quartiles)*		Diastolic blood pressure *(in quartiles)*	
Q1 (30-69 mmHg)	1 (ref)	Q1 (30-69 mmHg)	1 (ref)
Q2 (70-76 mmHg)	0.005792	Q2 (70-75 mmHg)	0.007047
Q3 (77-81 mmHg)	0.004878	Q3 (76-80 mmHg)	0.006125
Q4 (82-175 mmHg)	0.009052	Q4 (81-175 mmHg)	0.004957
Region		Region	
North East	1 (ref)	North East	1 (ref)
North West	0.002711	North West	0.005687
Yorkshire & The Humber	0.000865	Yorkshire & The Humber	0.000528
East Midlands	0.001643	East Midlands	0.002903
West Midlands	0.000535	West Midlands	0.0005
East of England	0.001026	East of England	0.000423
London	0.00405	London	0.002307
South East	0.005451	South East	0.001657
South West	0.001795	South West	0.005683
Polypharmacy (≥5 drugs)	0.004641	Polypharmacy (≥5 drugs)	0.002477
Medical history		Medical history	
COPD	0.003081	COPD	0.001859
Heart failure	0.001294	Heart failure	0.008399
Ischaemic heart disease	0.001797	Ischaemic heart disease	0.004416
Peptic ulcer	0.004548	Peptic ulcer	0.001113
Diabetes		Diabetes	
without insulin	0.001224	without insulin	0.003045
with insulin	0.006637	with insulin	0.001286
Peripheral arterial disease	0.000278	Peripheral arterial disease	0.007659
Atrial fibrillation	0.004183	Atrial fibrillation	0.001726
Venous thromboembolism	0.004937	Venous thromboembolism	0.006279
Any bleeding	0.00254	Any bleeding	0.000799
Stroke/TIA	0.001919	Stroke/TIA	0.005313
Chronic kidney disease		Chronic kidney disease	
Stage 3a	0.008651	Stage 3a	0.005831
Stage 3b	0.007943	Stage 3b	0.023673
Stage 4	0.014265	Stage 4	0.006376
Stage 5	0.01083	Stage 5	0.020549
No of GP active consultation in the past year		No of GP active consultation in the past year	
≥12 visits	1 (ref)	≥12 visits	1 (ref)

<12 visits	0.002669	<12 visits	0.003023
none	0.008137	none	0.002936

Abbreviation: DOAC, direct oral anticoagulants; IQR, interquartile range; COPD, chronic obstructive pulmonary disease; TIA, transient ischaemic attack; GP, general practice

Table S6. Standardised differences for the associations between concomitant use of DOACs and simvastatin and bleeding outcomes using active comparator in cohort study

Outcomes	Intracranial bleeding	Gastrointestinal bleeding	Other bleeding
Age group			
18-<40	1 (ref)	1 (ref)	1 (ref)
40-<50	0.021238	0.021233	0.021244
50-<60	0.004123	0.004134	0.004127
60-<70	0.016075	0.016062	0.016056
70-<80	0.003944	0.003948	0.00395
80+	**0.09992**	**0.099918**	**0.099924**
Male sex	0.004742	0.004724	0.004745
Body mass index			
Underweight	1 (ref)	1 (ref)	1 (ref)
Normal	0.00205	0.002115	0.002163
Overweight	0.003186	0.003132	0.003257
Obese	0.010638	0.010711	0.010706
Smoking status			
Non-smoker	1 (ref)	1 (ref)	1 (ref)
Current smoker	0.003557	0.00356	0.003563
Ex-smoker	0.00121	0.001215	0.00122
Index of Multiple Deprivation			
1 (least deprived)	1 (ref)	1 (ref)	1 (ref)
2	0.001305	0.001298	0.001304
3	0.002042	0.002044	0.00203
4	0.00145	0.001451	0.001451
5 (most deprived)	0.000993	0.000983	0.00099
Alcohol consumption			
Non-drinker	1 (ref)	1 (ref)	1 (ref)
Current low level	0.004931	0.004847	0.004862
Current Medium level	0.002621	0.002274	0.002625
Current high level	0.004752	0.004623	0.004703
Ex-drinker	0.009712	0.009713	0.009715
Current drinker with missing data on consumption level	0.004952	0.004955	0.004959
Systolic blood pressure *(in quartile)*			
Q1 (54-120 mmHg)	1 (ref)	1 (ref)	1 (ref)
Q2 (121-131 mmHg)	0.003562	0.003258	0.003555
Q3 (132-140 mmHg)	0.006681	0.006668	0.006843

Q4 (141-238 mmHg)	0.007905	0.007913	0.007869
Diastolic blood pressure *(in quartiles)*			
Q1 (30-69 mmHg)	1 (ref)	1 (ref)	1 (ref)
Q2 (70-75 mmHg)	0.009965	0.00999	0.00999
Q3 (76-80 mmHg)	0.003062	0.003062	0.00306
Q4 (81-175 mmHg)	0.007946	0.007963	0.007986
Region			
North East	1 (ref)	1 (ref)	1 (ref)
North West	0.005727	0.005733	0.005729
Yorkshire & The Humber	0.002282	0.002285	0.002299
East Midlands	0.002219	0.002223	0.002225
West Midlands	0.000377	0.000388	0.000379
East of England	0.003397	0.003386	0.00338
London	0.001316	0.001306	0.001336
South East	0.000426	0.000422	0.000425
South West	0.004705	0.004715	0.004709
Polypharmacy (≥5 drugs)	0.0038	0.003799	0.0038
Medical history			
COPD	0.001752	0.001763	0.001758
Heart failure	0.009701	0.009702	0.009711
Ischaemic heart disease	0.005724	0.005715	0.005713
Peptic ulcer	0.001324	0.001329	0.001328
Diabetes			
without insulin	0.001087	0.001091	0.001104
with insulin	0.001536	0.001537	0.001527
Peripheral arterial disease	0.005968	0.00596	0.005962
Venous thromboembolism	0.00683	0.006995	0.006832
Any bleeding	0.003003	0.002899	0.002965
Stroke/TIA	0.013179	0.013181	0.013176
Chronic kidney disease			
Stage 3a	0.004793	0.004784	0.004797
Stage 3b	0.021501	0.021478	0.021479
Stage 4	0.009051	0.009035	0.009042
Stage 5	0.013339	0.013336	0.013343
Medication use in the past 3 months			
PPIs	0.006358	0.00637	0.006362
aspirin	0.002306	0.002285	0.002316
antiplatelet	0.005128	0.00513	0.005134
SSRI/SNRI	0.005024	0.005019	0.005017

anticonvulsant	0.004632	0.004641	0.004628
ACEI	0.004298	0.004336	0.004269
NSAIDs	0.010254	0.010265	0.010251
Oral corticosteroids	0.004139	0.004088	0.004117
Macrolides	0.001462	0.001471	0.001471
No of GP active consultation in the past year			
≥12 visits	1 (ref)	1 (ref)	1 (ref)
<12 visits	0.002646	0.002649	0.002644
none	0.002556	0.00256	0.002548

Abbreviation: DOAC, direct oral anticoagulants; PPIs, proton pump inhibitors; SSRI/SNRI, SSRI, selective serotonin reuptake inhibitors/serotonin and norepinephrine reuptake inhibitors; IQR, interquartile range; COPD, chronic obstructive pulmonary disease; TIA, transient ischaemic attack; ACEI, angiotensin-converting enzyme inhibitors; CCBs, calcium channel blockers; NSAIDs, non-steroidal anti-inflammatory drugs; GP, general practice

Table S7. Standardised differences for the associations between concomitant use of DOACs and simvastatin and cardiovascular outcomes using active comparator in cohort study

	Ischaemic stroke	Myocardial infarction	Venous thromboembolism	Cardiovascular mortality
Age group				
18-<40	1 (ref)	1 (ref)	1 (ref)	1 (ref)
40-<50	0.024093	0.024096	0.024069	0.024084
50-<60	0.009054	0.009045	0.009123	0.009053
60-<70	0.016585	0.016579	0.016623	0.016575
70-<80	0.004541	0.004539	0.0043	0.004568
80+	**0.099565**	**0.099591**	**0.099552**	**0.099595**
Male sex	0.005965	0.005958	0.006021	0.005964
Body mass index				
Underweight	1 (ref)	1 (ref)	1 (ref)	1 (ref)
Normal	0.001854	0.00173	0.001701	0.001735
Overweight	0.002437	0.002376	0.00216	0.002378
Obese	0.009258	0.009331	0.009223	0.009339
Smoking status				
Non-smoker	1 (ref)	1 (ref)	1 (ref)	1 (ref)
Current smoker	0.006993	0.006988	0.007163	0.006951
Ex-smoker	0.004196	0.004186	0.004499	0.004172
Index of Multiple Deprivation				
1 (least deprived)	1 (ref)	1 (ref)	1 (ref)	1 (ref)
2	0.001333	0.001325	0.001389	0.001317
3	0.000627	0.000616	0.00059	0.000598
4	0.001175	0.001173	0.00139	0.001221
5 (most deprived)	0.001032	0.001039	0.00118	0.001024
Alcohol consumption				
Non-drinker	1 (ref)	1 (ref)	1 (ref)	1 (ref)
Current low level	0.005593	0.005522	0.005367	0.005474
Current Medium level	0.003122	0.003207	0.002503	0.003228
Current high level	0.004511	0.004496	0.004504	0.004643
Ex-drinker	0.009201	0.009184	0.007856	0.008876
Current drinker with missing data on consumption level	0.003755	0.003756	0.004571	0.003619
Systolic blood pressure *(in quartile)*				
Q1 (54-120 mmHg)	1 (ref)	1 (ref)	1 (ref)	1 (ref)
Q2 (121-131 mmHg)	0.00377	0.003981	0.003533	0.004084

Q3 (132-140 mmHg)	0.007034	0.007037	0.006059	0.007386
Q4 (141-238 mmHg)	0.006305	0.006472	0.00641	0.006528
Diastolic blood pressure *(in quartiles)*				
Q1 (30-69 mmHg)	1 (ref)	1 (ref)	1 (ref)	1 (ref)
Q2 (70-75 mmHg)	0.007354	0.007352	0.003273	0.007389
Q3 (76-80 mmHg)	0.003605	0.003369	0.004617	0.003586
Q4 (81-175 mmHg)	0.006886	0.00668	0.004632	0.006724
Region				
North East	1 (ref)	1 (ref)	1 (ref)	1 (ref)
North West	0.005185	0.005171	0.005224	0.005173
Yorkshire & The Humber	0.00048	0.000484	0.000462	0.000488
East Midlands	0.003607	0.003606	0.003526	0.003625
West Midlands	0.001776	0.001779	0.001889	0.001775
East of England	0.002849	0.002858	0.002626	0.00285
London	0.00157	0.001556	0.001682	0.00157
South East	0.0012	0.001215	0.001203	0.00119
South West	0.006783	0.006786	0.006597	0.006796
Polypharmacy (≥5 drugs)	0.00136	0.001367	0.001057	0.001333
Medical history				
COPD	0.001274	0.001279	0.001094	0.001246
Heart failure	0.011947	0.011891	0.011931	0.011892
Ischaemic heart disease	0.006578	0.006566	0.006778	0.006571
Diabetes				
without insulin	0.00329	0.003295	0.003107	0.003262
with insulin	0.001342	0.001351	0.00147	0.001351
Peripheral arterial disease	0.007695	0.007679	0.007777	0.007688
Atrial fibrillation	0.005161	0.005154	0.005223	0.005156
Venous thromboembolism	0.000966	0.000946	0.001124	0.000952
Stroke/TIA	0.008218	0.008219	0.008123	0.008226
Chronic kidney disease				
Stage 3a	0.003148	0.003158	0.003065	0.003138
Stage 3b	0.023149	0.023262	0.023396	0.023141
Stage 4	0.008949	0.008966	0.008895	0.008964
Stage 5	0.015945	0.015941	0.015942	0.015951
Medication use in the past 3 months				
diazepam	0.000251	0.000249	0.001065	0.000238
aspirin	0.00479	0.004783	0.004821	0.004772

antiplatelet	0.006791	0.006792	0.00678	0.006785
amiodarone	0.001093	0.00109	0.000978	0.001091
ACEIs	0.004106	0.004101	0.004112	0.00409
ARBs	0.003988	0.003984	0.003958	0.003993
CCBs	0.007453	0.007447	0.007473	0.00744
Beta blockers	0.000958	0.000956	0.000954	0.000955
NSAIDs	0.011517	0.011509	0.011522	0.011525
Macrolides	0.000393	0.000396	0.000277	0.000397
Oestrogen/oestrogen like drugs	0.001694	0.001711	0.001739	0.001707
No of GP active consultation in the past year				
≥12 visits	1 (ref)	1 (ref)	1 (ref)	1 (ref)
<12 visits	0.001657	0.00165	0.001573	0.001634
none	0.003218	0.003221	0.002865	0.003222

Abbreviation: DOAC, direct oral anticoagulants; IQR, interquartile range; COPD, chronic obstructive pulmonary disease; TIA, transient ischaemic attack; ACEI, angiotensin-converting enzyme inhibitors; ARB, angiotensin receptor blockers; CCBs, calcium channel blockers; NSAIDs, non-steroidal anti-inflammatory drugs; GP, general practice

Figure S7. Flow diagram for inclusion in case-crossover study design

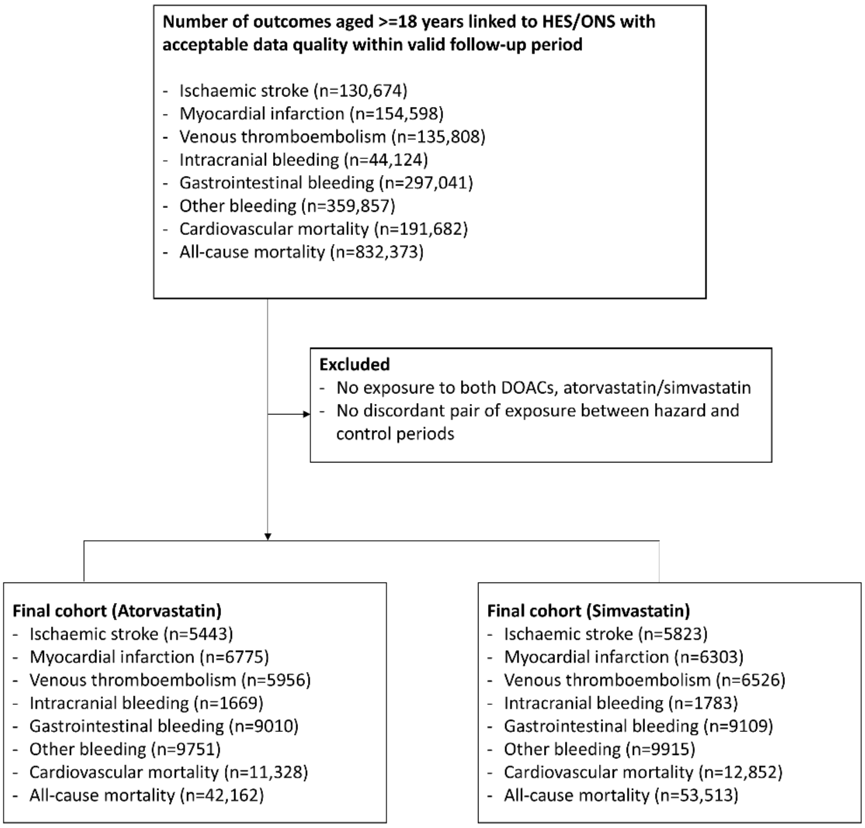

Abbreviation: DOAC, direct oral anticoagulants; HES, Hospital Episode Statistics; ONS, Office for National Statistics.

Figure S8. More detailed version of Figure 1 from the main text

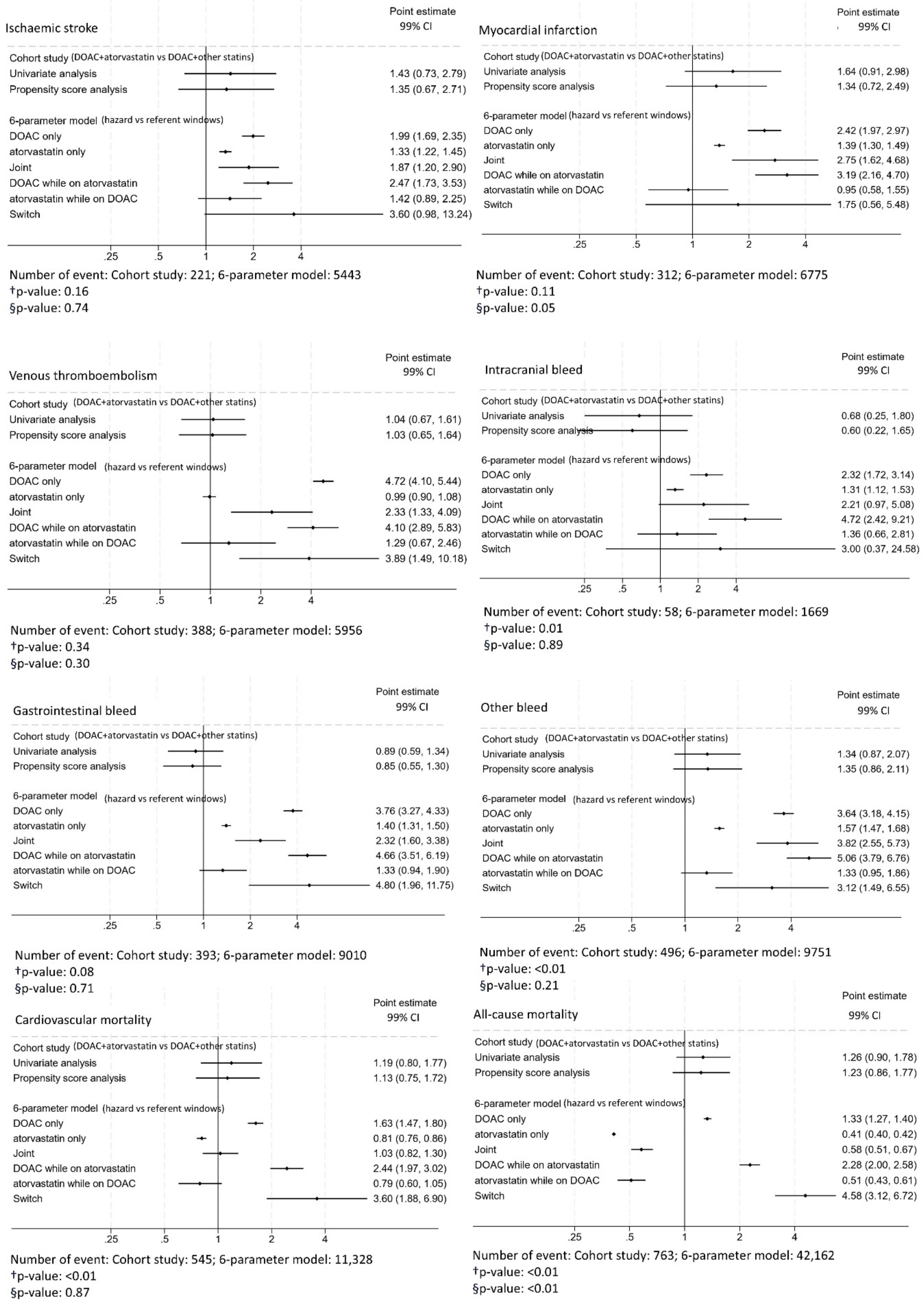

Abbreviation: DOAC, direct oral anticoagulants
Point estimate = HR in cohort study; OR in case-crossover study design.
In 6 parameter model, **joint** indicates initiate both drugs simultaneously in hazard or control window, **DOAC while on atorvastatin** indicates initiation of DOACs in the presence of atorvastatin, **atorvastatin while on DOAC** indicates initiation of atorvastatin in the presence of DOACs, **switch** indicates use of one drug in the hazard window and the other drug in the control window.
†testing the equality of coefficients of parameters of **DOAC while on atorvastatin** and **DOAC only**
§testing the equality of coefficients of parameters of **atorvastatin while on DOAC** and **atorvastatin only**

Figure S9 Kaplan-Meier curves for the association between DOAC + atorvastatin and different outcomes, versus DOAC + other statins

Ischaemic stroke

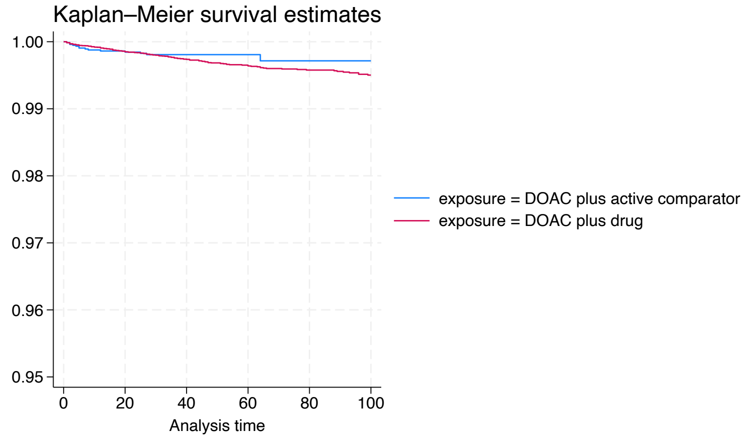

Myocardial infarction

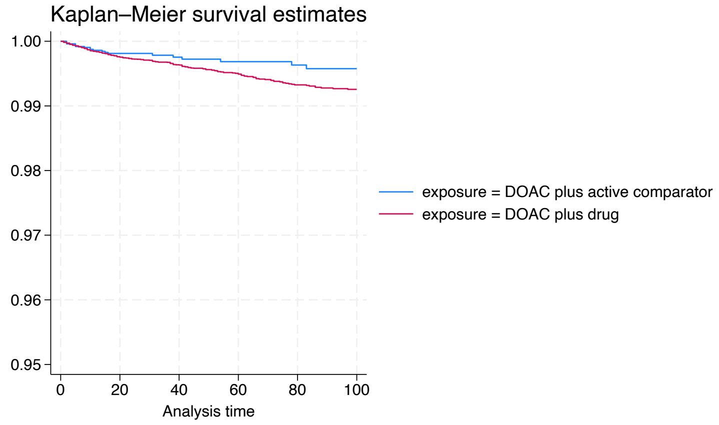

Venous thromboembolism

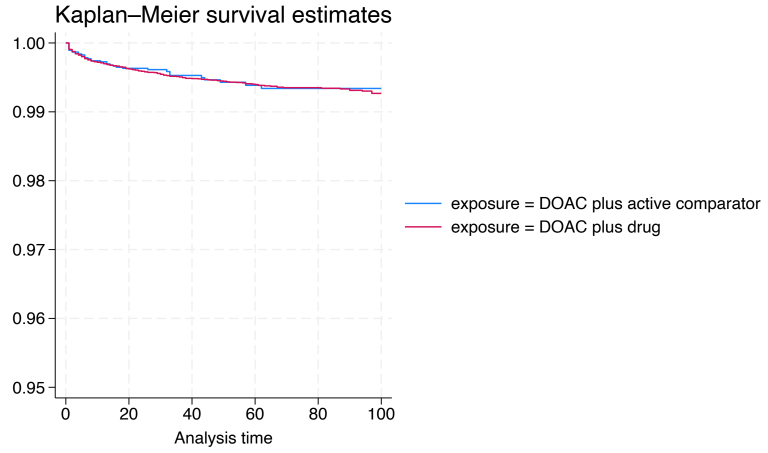

Intracranial bleeding

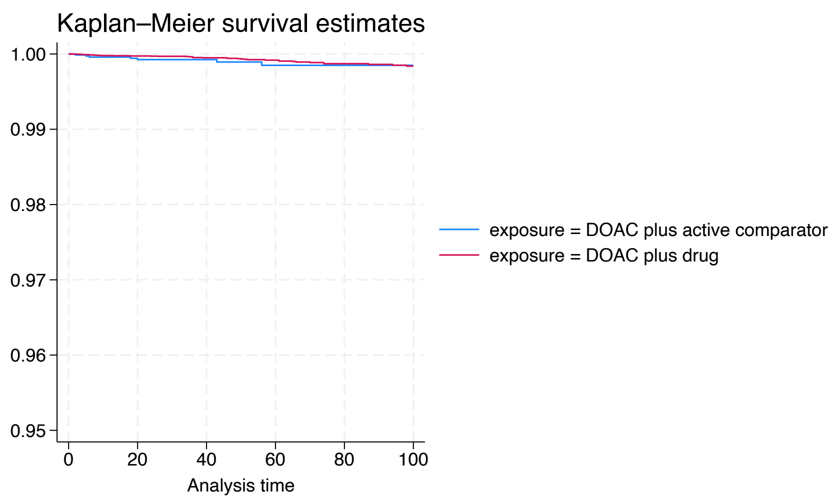

Gastrointestinal bleeding

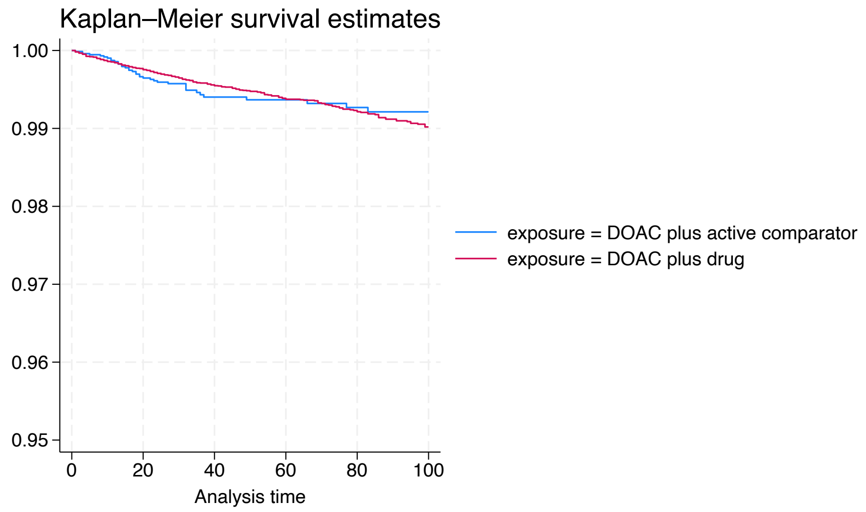

Other bleeding

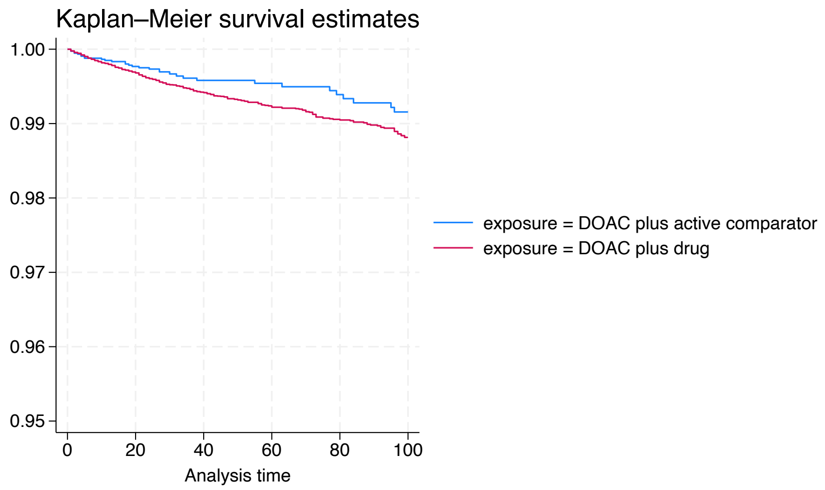

Cardiovascular mortality

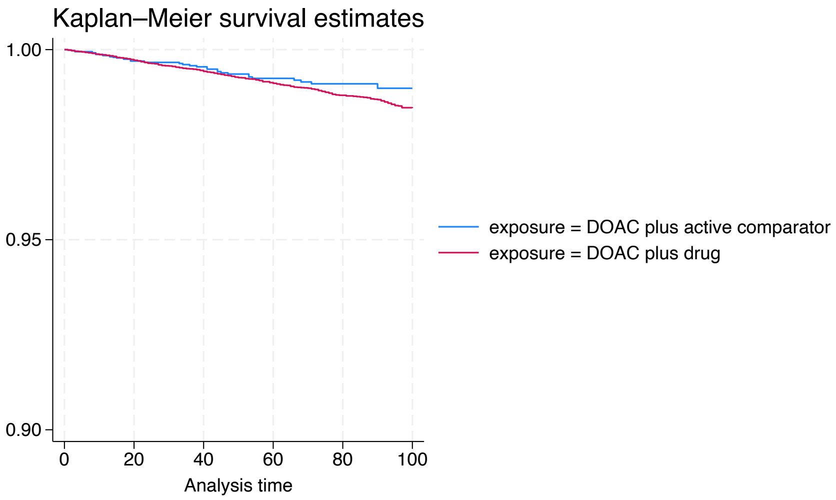

All-cause mortality

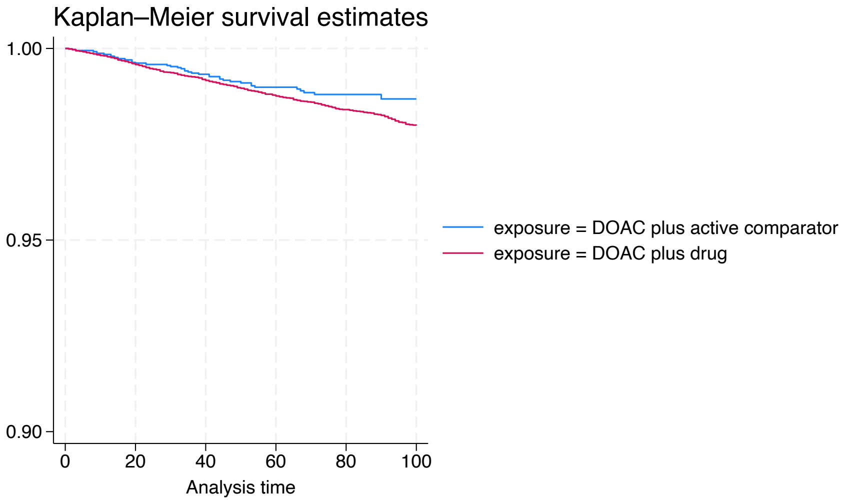

Table S8. Details of results from univariable and propensity score models for the association between concomitant use of DOACs and atorvastatin and outcomes using active comparator and DOAC alone groups as comparison groups respectively in cohort study

	Main analysis using DOAC + other statins group							Sensitivity ana		
	Number of events	Total person-year	Rate per 1,000	Univariable model		Propensity score model		Number of events	Total person-year	Rate per 1,000
				HR	99% CI	HR	99% CI			
**Ischaemic stroke**										
Comparison group	16	1061	15.08	1.00 (ref)		1 (ref)		439	30474	14.41
DOAC + atorvastatin	205	9741	21.04	1.43	0.73 - 2.79	1.35	0.67 – 2.71	205	9750	21.03
**Intracranial bleeding**										
Comparison group	8	1062	7.53	1.00 (ref)		1 (ref)		127	30512	4.16
DOAC + atorvastatin	50	9766	5.12	0.68	0.25 - 1.80	0.60	0.22 – 1.65	50	9775	5.12
**Gastrointestinal bleeding**										
Comparison group	44	1060	41.52	1.00 (ref)		1 (ref)		1103	30374	36.31
DOAC + atorvastatin	349	9734	35.85	0.89	0.59 - 1.34	0.85	0.55 - 1.30	351	9742	36.03
**Other bleeding**										
Comparison group	38	1058	35.91	1.00 (ref)		1 (ref)		1282	30328	42.27
DOAC + atorvastatin	458	9713	47.16	1.34	0.87 - 2.07	1.35	0.86 – 2.11	459	9721	47.22
**Myocardial infarction**										
Comparison group	20	1061	18.84	1.00 (ref)		1 (ref)		394	30493	12.92
DOAC + atorvastatin	292	9749	29.95	1.64	0.91 - 2.98	1.34	0.72 - 2.49	292	9758	29.93
**Venous thromboembolism**										
Comparison group	39	1059	36.82	1.00 (ref)		1 (ref)		2208	30233	73.03
DOAC + atorvastatin	349	9737	35.84	1.04	0.67 - 1.61	1.03	0.65 - 1.64	349	9746	35.81
**All-cause mortality**										
Comparison group	61	1062	57.41	1.00 (ref)		1 (ref)		2942	30518	96.4
DOAC + atorvastatin	702	9772	71.84	1.26	0.90 - 1.78	1.23	0.86 - 1.77	702	9780	71.78
**Cardiovascular mortality**										
Comparison group	46	1062	43.29	1.00 (ref)		1 (ref)		1393	30518	45.65
DOAC + atorvastatin	499	9772	51.07	1.19	0.80 - 1.77	1.13	0.75 – 1.72	499	9780	51.02

Figure S10. More detailed version of Figure 2 from the main text

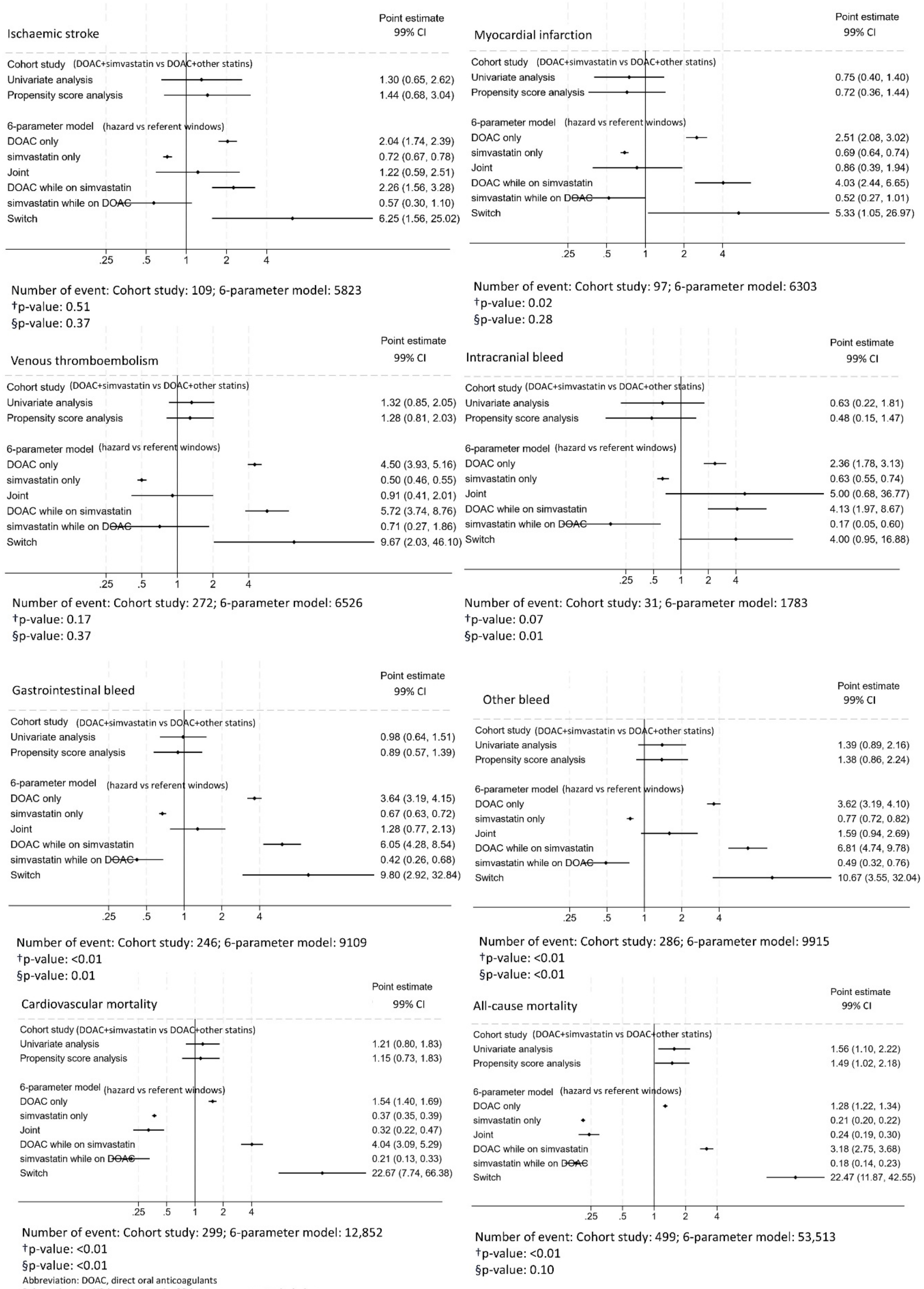

Abbreviation: DOAC, direct oral anticoagulants

Point estimate = HR in cohort study; OR in case-crossover study design

In 6 parameter model, **joint** indicates initiate both drugs simultaneously in hazard or control window, **DOAC while on simvastatin** indicates initiation of DOACs in the presence of simvastatin, **simvastatin while on DOAC** indicates initiation of simvastatin in the presence of DOACs, **switch** indicates use of one drug in the hazard window and the other drug in the control window.

†testing the equality of coefficients of parameters of **DOAC while on simvastatin** and **DOAC only**

§testing the equality of coefficients of parameters of **simvastatin while on DOAC** and **simvastatin only**

Figure S11. Kaplan-Meier curves for the association between DOAC + simvastatin and different outcomes, versus DOAC + other statins

Ischaemic stroke

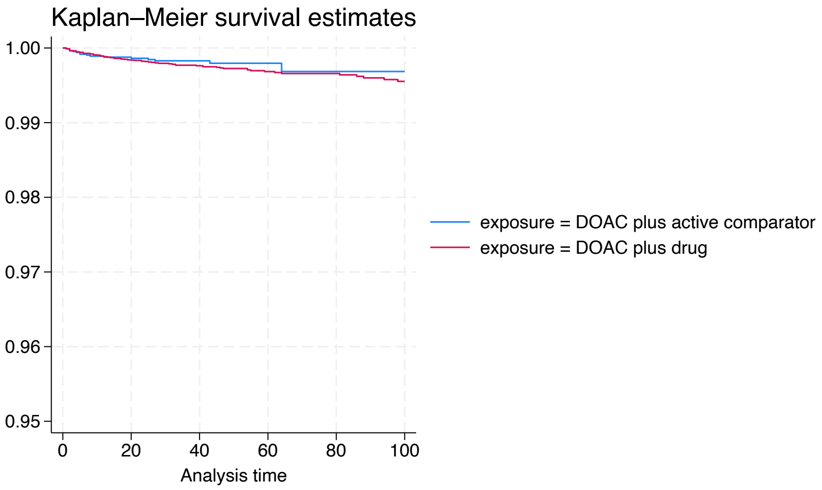

Myocardial infarction

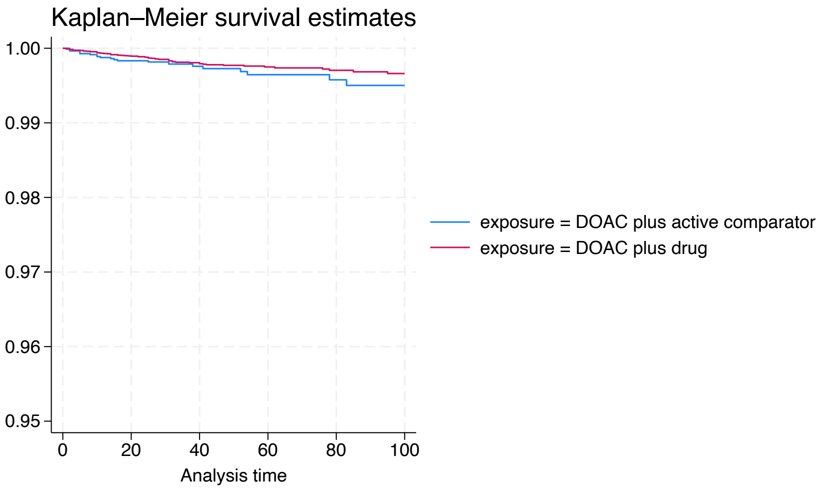

Venous thromboembolism

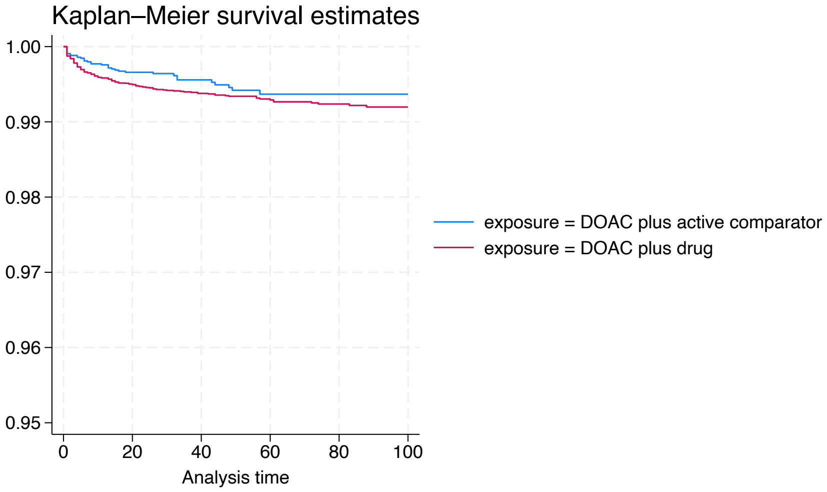

Intracranial bleeding

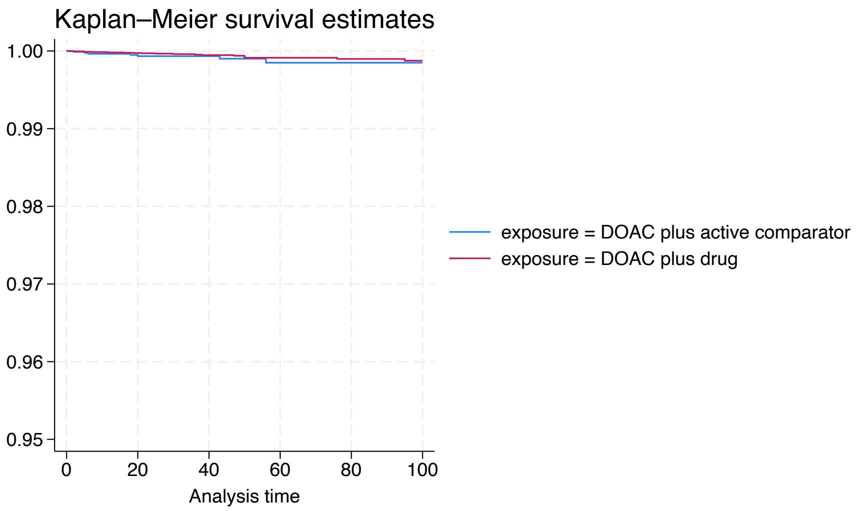

Gastrointestinal bleeding

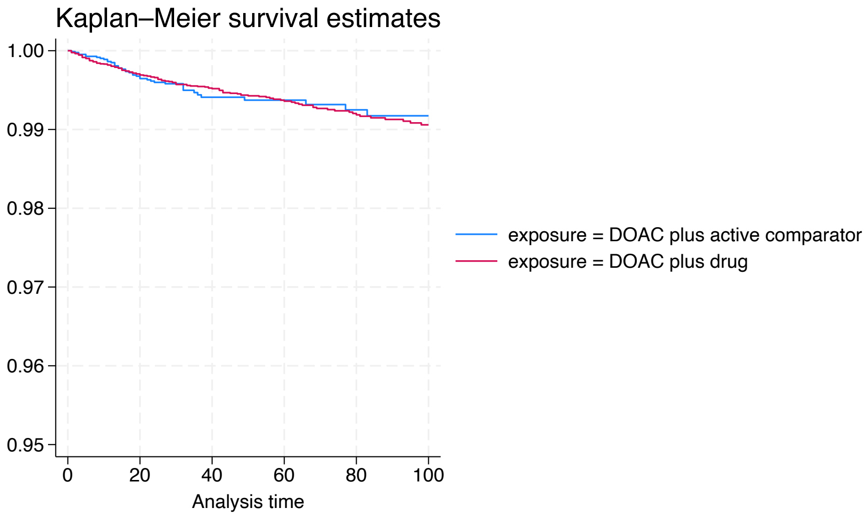

Other bleeding

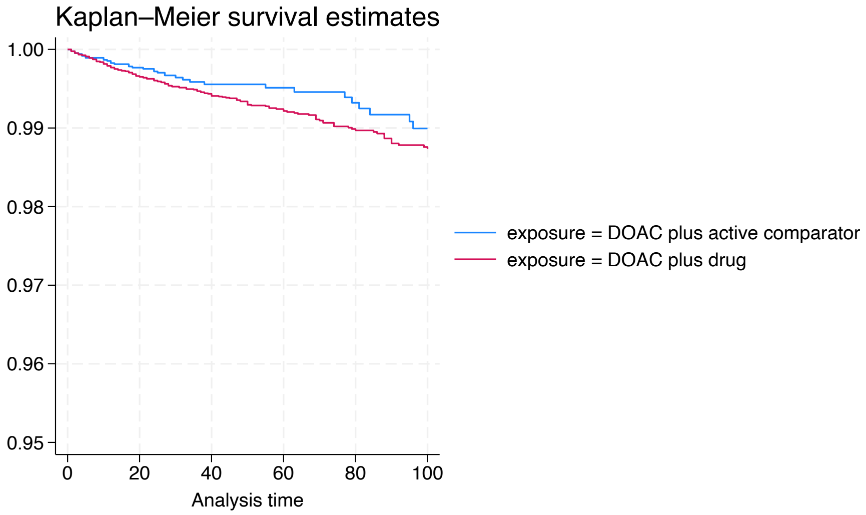

Cardiovascular mortality

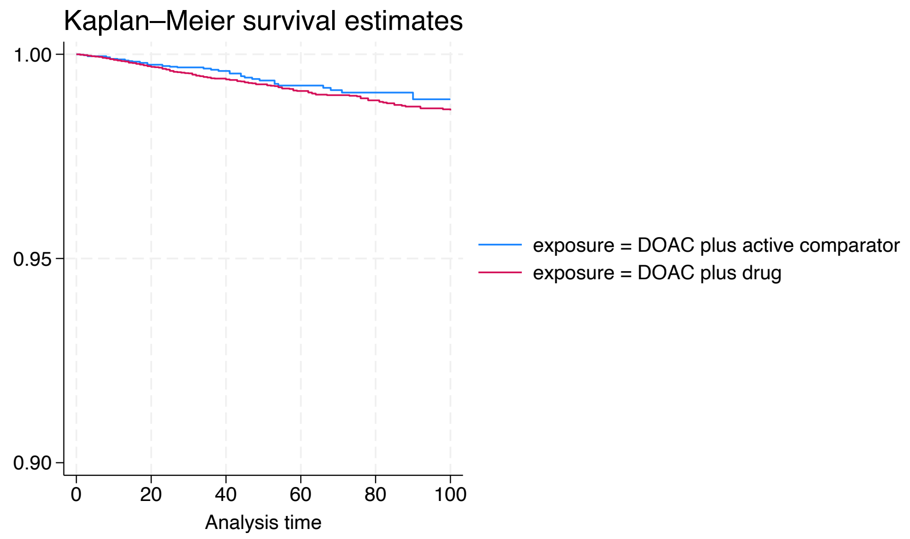

All-cause mortality

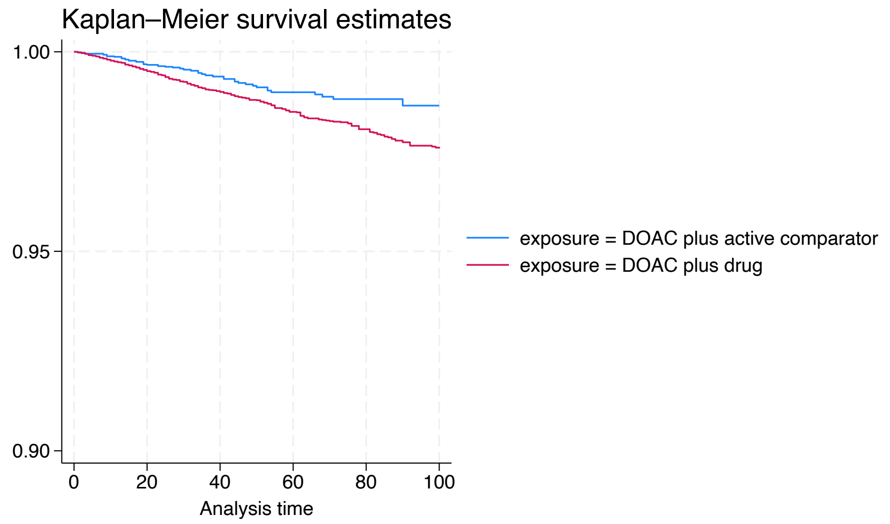

Table S9. Details of results from univariable and propensity score models for the association between concomitant use of DOACs and simvastatin and outcomes using active comparator and DOAC alone groups as comparison groups respectively in cohort study

	Main analysis using DOAC + other statins group							Sensitivity analysis using DOAC alone group						
	Number of events	Total person-year	Rate per 1,000	Univariable model		Propensity score model		Number of events	Total person-year	Rate per 1,000	Univariable model		Propensity score model	
				HR	99% CI	HR	99% CI				HR	99% CI	HR	99% CI
**Ischaemic stroke**														
Comparison group	16	1095	14.61	1.00 (ref)		1 (ref)		520	34498	15.07	1.00 (ref)		1 (ref)	
DOAC + simvastatin	93	5075	18.33	1.30	0.65 - 2.62	1.44	0.68 – 3.04	93	5076	18.32	1.12	0.84 - 1.49	0.98	0.70 – 1.36
**Intracranial bleeding**														
Comparison group	8	1096	7.3	1.00 (ref)		1 (ref)		150	34566	4.34	1.00 (ref)		1 (ref)	
DOAC + simvastatin	23	5083	4.53	0.63	0.22 - 1.81	0.48	0.15 - 1.47	23	5084	4.52	1.04	0.59 - 1.86	0.97	0.51 – 1.82
**Gastrointestinal bleeding**														
Comparison group	45	1094	41.13	1.00 (ref)		1 (ref)		1252	34408	36.39	1.00 (ref)		1 (ref)	
DOAC + simvastatin	201	5060	39.72	0.98	0.64 - 1.51	0.89	0.57 - 1.39	201	5062	39.71	1.02	0.84 - 1.24	0.90	0.72 - 1.13
**Other bleeding**														
Comparison group	39	1093	35.7	1.00 (ref)		1 (ref)		1442	34351	41.98	1.00 (ref)		1 (ref)	
DOAC + simvastatin	247	5057	48.84	1.39	0.89 - 2.16	1.38	0.86 – 2.24	247	5058	48.83	1.09	0.91 - 1.30	0.99	0.80 – 1.23
**Myocardial infarction**														
Comparison group	22	1096	20.08	1.00 (ref)		1 (ref)		575	34521	16.66	1.00 (ref)		1 (ref)	
DOAC + simvastatin	75	5078	14.77	0.75	0.40 - 1.40	0.72	0.36 - 1.44	75	5080	14.76	0.83	0.60 - 1.14	0.60	0.43 – 0.85
**Venous thromboembolism**														
Comparison group	40	1093	36.59	1.00 (ref)		1 (ref)		2296	34277	66.98	1.00 (ref)		1 (ref)	
DOAC + simvastatin	232	5060	45.85	1.32	0.85 - 2.05	1.28	0.81 – 2.03	232	5062	45.83	0.56	0.47 - 0.67	0.70	0.56 – 0.86
**All-cause mortality**														

Comparison group	61	1097	55.62	1.00 (ref)		1 (ref)		3122	34575	90.3	1.00 (ref)		1 (ref)	
DOAC + simvastatin	438	5084	86.16	1.56	1.10 - 2.22	1.49	1.02 – 2.18	439	5085	86.33	0.93	0.81 - 1.06	0.77	0.67 – 0.89
**Cardiovascular mortality**														
Comparison group	46	1097	41.94	1.00 (ref)		1 (ref)		1599	34575	46.25	1.00 (ref)		1 (ref)	
DOAC + simvastatin	253	5084	49.77	1.21	0.80 - 1.83	1.15	0.73 – 1.83	254	5085	49.95	1.05	0.88 - 1.25	0.81	0.67 – 0.97

Table S10. Subgroup analysis for the association between concomitant use of DOAC and atorvastatin/simvastatin and all-cause mortality, compared with DOAC and other statins in cohort study

	Atorvastatin				Simvastatin			
**Subgroup**	**HR**	**99% lower CI**	**99% Upper CI**	**Interaction p-value**	**HR**	**99% lower CI**	**99% Upper CI**	**Interaction p-value**
**Age at cohort entry**								
≥ 18 -< 65	0.50	0.14	1.80		0.66	0.16	2.72	
≥ 65 -< 75	1.21	0.58	2.53		1.54	0.70	3.38	
≥ 75	1.35	0.88	2.07	0.16	1.53	0.97	2.42	0.33
**Bodyweight**								
≤ 60 kg	1.06	0.51	2.20		1.25	0.55	2.85	
> 60 -≤ 120 kg	1.41	0.91	2.18		1.70	1.07	2.70	
> 120 kg	0.38	0.10	1.38	0.09	0.24	0.05	1.05	0.01
**Potential indication**								
Atrial fibrillation	1.16	0.78	1.72	0.36	1.36	0.90	2.06	0.31
Venous thromboembolism	1.76	0.70	4.46	0.27	1.82	0.68	4.89	0.53
**Polypharmacy**								
No of drugs <5	0.32	0.06	1.80		0.44	0.07	2.80	
No of drugs ≥5	1.30	0.90	1.87	0.04	1.55	1.05	2.29	0.09
**Sex**								
female	1.23	0.72	2.08		1.73	1.01	2.95	
male	1.24	0.76	2.02	0.97	1.29	0.76	2.21	0.33
**Individual DOAC**								
dabigatran	8.78	0.64	120.19		4.82	0.72	32.09	
rivaroxaban	1.08	0.63	1.83		1.29	0.72	2.31	
apixaban	1.21	0.72	2.03		1.64	0.96	2.83	
edoxaban	1.22	0.25	5.92	0.25	0.93	0.18	4.97	0.30
**Order of initiation**								
Initiation of DOAC	1.77	0.62	5.07		3.16	1.07	9.34	
Initiation of amiodarone/diltiazem	1.03	0.65	1.63		1.17	0.72	1.90	
Both drugs initiate together	1.26	0.63	2.52	0.44	1.60	0.77	3.34	0.09
**Level of dose***								
low dose	1.24	0.68	2.27		1.37	0.75	2.51	
high dose	1.14	0.67	1.94	0.79	1.36	0.76	2.43	0.97

*Only restricted the cohort to people with atrial fibrillation in this analysis
The power for conducting subgroup analyses for renal function was inadequate for either atorvastatin/simvastatin.

Table S11. Subgroup analysis for the association between concomitant use of DOAC and atorvastatin/simvastatin and cardiovascular mortality, compared with DOAC and other statins in cohort study

	Atorvastatin				Simvastatin			
**Subgroup**	**HR**	**99% lower CI**	**99% Upper CI**	**Interaction p-value**	**HR**	**99% lower CI**	**99% Upper CI**	**Interaction p-value**
**Age at cohort entry**								
≥ 18 -< 65	0.67	0.09	4.89		0.94	0.10	8.34	
≥ 65 -< 75	0.94	0.42	2.12		1.25	0.52	3.00	
≥ 75	1.25	0.76	2.05	0.59	1.13	0.66	1.94	0.94
**Bodyweight**								
≤ 60 kg	0.92	0.38	2.21		1.00	0.37	2.68	
> 60 -≤ 120 kg	1.23	0.75	2.02		1.22	0.71	2.11	
> 120 kg	1.06	0.16	7.30	0.74	0.48	0.05	4.39	0.74
**Potential indication**								
Atrial fibrillation	1.00	0.64	1.57	0.06	0.97	0.59	1.59	0.02
Venous thromboembolism	4.21	0.92	19.25	0.02	2.75	0.57	13.21	0.12
**Polypharmacy**								
No of drugs <5	0.52	0.03	8.08		0.35	0.01	8.31	
No of drugs ≥5	1.16	0.76	1.78	0.46	1.17	0.74	1.87	0.33
**Sex**								
female	1.18	0.62	2.21		1.22	0.61	2.45	
male	1.11	0.64	1.93	0.85	1.10	0.60	2.01	0.77
**Individual DOAC**								
dabigatran	4.89	0.35	68.33		2.92	0.40	21.61	
rivaroxaban	1.14	0.60	2.18		1.11	0.52	2.39	
apixaban	0.99	0.55	1.78		1.16	0.62	2.17	
edoxaban	1.28	0.19	8.75	0.49	0.51	0.06	4.47	0.50
**Order of initiation**								
Initiation of DOAC	1.32	0.42	4.09		2.05	0.59	7.11	
Initiation of amiodarone/diltiazem	1.03	0.58	1.82		1.01	0.54	1.88	
Both drugs initiate together	1.00	0.48	2.10	0.86	1.05	0.48	2.33	0.40
**Level of dose***								
low dose	1.04	0.52	2.07		0.90	0.45	1.82	
high dose	1.00	0.54	1.84	0.91	0.99	0.48	2.01	0.82

*Only restricted the cohort to people with atrial fibrillation in this analysis

The power for conducting subgroup analyses for renal function was inadequate for either atorvastatin/simvastatin.

Table S12. Subgroup analysis for the association between concomitant use of DOAC and atorvastatin/simvastatin and ischaemic stroke, compared with DOAC and other statins in cohort study

	Atorvastatin				Simvastatin			
**Subgroup**	**HR**	**99% lower CI**	**99% Upper CI**	**Interaction p-value**	**HR**	**99% lower CI**	**99% Upper CI**	**Interacti on p-value**
**Age at cohort entry**								
≥ 18 -< 65	1.55	0.11	21.50		3.38	0.21	53.50	
≥ 65 -< 75	0.93	0.30	2.94		0.91	0.26	3.18	
≥ 75	1.57	0.62	3.98	0.66	1.59	0.60	4.22	0.46
**Potential indication**								
Atrial fibrillation	1.35	0.62	2.93	0.98	1.33	0.58	3.07	0.57
Venous thromboembolism	1.65	0.25	11.07	0.77	2.60	0.36	18.69	0.41
**Sex**								
female	1.31	0.51	3.38		1.24	0.46	3.34	
male	1.39	0.50	3.90	0.91	1.72	0.55	5.38	0.57
**Order of initiation**								
Initiation of DOAC	1.20	0.24	5.92		1.24	0.20	7.73	
Initiation of amiodarone/diltiazem	1.41	0.51	3.90		1.40	0.49	3.98	
Both drugs initiate together	1.08	0.32	3.69	0.91	1.39	0.38	5.14	0.99
**Level of dose***								
low dose	0.71	0.23	2.23		1.31	0.39	4.38	
high dose	1.99	0.69	5.76	0.09	1.14	0.35	3.68	0.83

*Only restricted the cohort to people with atrial fibrillation in this analysis
The power for conducting subgroup analyses for bodyweight, polypharmacy, renal function and type of DOACs was inadequate for either atorvastatin/simvastatin.

Table S13. Subgroup analysis for the association between concomitant use of DOAC and atorvastatin/simvastatin and myocardial infarction, compared with DOAC and other statins in cohort study

	Atorvastatin				Simvastatin			
**Subgroup**	**HR**	**99% lower CI**	**99% Upper CI**	**Interaction p-value**	**HR**	**99% lower CI**	**99% Upper CI**	**Interaction p-value**
**Age at cohort entry**								
≥ 18 -< 65	1.80	0.13	24.82		0.78	0.04	14.10	
≥ 65 -< 75	1.34	0.43	4.13		0.83	0.25	2.72	
≥ 75	1.29	0.60	2.78	0.95	0.68	0.29	1.61	0.93
**Potential indication**								
Atrial fibrillation	1.34	0.67	2.66	0.99	0.64	0.29	1.40	0.38
Venous thromboembolism	2.92	0.45	18.99	0.26	1.56	0.19	12.42	0.33
**Polypharmacy**								
No of drugs <5	0.72	0.04	12.12		NA	NA	NA	
No of drugs ≥5	1.37	0.73	2.57	0.56	NA	NA	NA	
**Sex**								
female	1.70	0.55	5.25		1.11	0.34	3.61	
male	1.17	0.56	2.45	0.47	0.57	0.24	1.35	0.24
**Individual DOAC**								
dabigatran	0.77	0.11	5.28		NA	NA	NA	
rivaroxaban	2.02	0.70	5.78		NA	NA	NA	
apixaban	1.13	0.48	2.68		NA	NA	NA	
edoxaban	0.85	0.06	12.35	0.57	NA	NA	NA	
**Order of initiation**								
Initiation of DOAC	2.90	0.44	18.98		1.29	0.20	8.20	
Initiation of amiodarone/diltiazem	1.47	0.55	3.95		0.87	0.31	2.48	
Both drugs initiate together	0.73	0.31	1.76	0.15	0.34	0.11	1.02	0.15
**Level of dose***								
low dose	1.90	0.56	6.46		0.77	0.20	3.05	
high dose	1.07	0.44	2.57	0.33	0.55	0.21	1.46	0.60

*Only restricted the cohort to people with atrial fibrillation in this analysis

NA, not applicable due to inadequate power to conduct subgroup analysis for either atorvastatin/simvastatin.

The power for conducting subgroup analyses for bodyweight, and renal function was inadequate for either atorvastatin/simvastatin.

Table S14. Subgroup analysis for the association between concomitant use of DOAC and atorvastatin/simvastatin and venous thromboembolism, compared with DOAC and other statins in cohort study

	Atorvastatin				Simvastatin			
**Subgroup**	**HR**	**99% lower CI**	**99% Upper CI**	**Interaction p-value**	**HR**	**99% lower CI**	**99% Upper CI**	**Interaction p-value**
**Age at cohort entry**								
≥ 18 -< 65	0.74	0.28	2.00		0.81	0.28	2.34	
≥ 65 -< 75	1.17	0.50	2.77		1.50	0.62	3.62	
≥ 75	1.12	0.59	2.14	0.61	1.35	0.72	2.54	0.48
**Potential indication**								
Atrial fibrillation	1.56	0.56	4.34	0.26	1.81	0.64	5.14	0.37
Venous thromboembolism	1.06	0.60	1.88	0.90	1.29	0.73	2.28	0.94
**Polypharmacy**								
No of drugs <5	2.37	0.17	32.95		4.64	0.32	66.36	
No of drugs ≥5	0.99	0.62	1.59	0.40	1.21	0.76	1.94	0.20
**Sex**								
female	0.90	0.47	1.70		1.29	0.68	2.45	
male	1.19	0.61	2.33	0.43	1.27	0.65	2.47	0.96
**Individual DOAC**								
dabigatran	0.44	0.02	10.33		0.19	0.00	7.35	
rivaroxaban	0.92	0.50	1.68		1.11	0.61	2.02	
apixaban	1.16	0.53	2.51		1.60	0.75	3.43	
edoxaban	2.49	0.18	35.27	0.66	2.98	0.19	46.62	0.33
**Order of initiation**								
Initiation of DOAC	5.83	0.43	79.19		4.48	0.63	32.05	
Initiation of amiodarone/diltiazem	1.08	0.64	1.83		1.13	0.67	1.90	
Both drugs initiate together	0.56	0.19	1.63	0.07	0.86	0.29	2.56	0.16
**Level of dose***								
low dose	4.42	0.32	61.30		5.40	0.38	76.09	
high dose	1.14	0.37	3.50	0.22	1.27	0.40	4.03	0.20

*Only restricted the cohort to people with atrial fibrillation in this analysis
The power for conducting subgroup analyses for bodyweight, and renal function was inadequate for either atorvastatin/simvastatin.

Table S15. Subgroup analysis for the association between concomitant use of DOAC and atorvastatin/simvastatin and gastrointestinal bleeding, compared with DOAC and other statins in cohort study

	Atorvastatin				Simvastatin			
Subgroup	HR	99% lower CI	99% Upper CI	Interaction p-value	HR	99% lower CI	99% Upper CI	Interaction p-value
**Age at cohort entry**								
≥ 18 -< 65	0.89	0.18	4.33		0.65	0.14	2.95	
≥ 65 -< 75	1.15	0.45	2.92		0.97	0.37	2.54	
≥ 75	0.77	0.46	1.27	0.62	0.89	0.53	1.52	0.84
**Potential indication**								
Atrial fibrillation	0.74	0.47	1.18	0.08	0.75	0.46	1.24	0.05
Venous thromboembolism	1.07	0.42	2.72	0.48	1.54	0.56	4.21	0.12
**Renal function**								
no CKD	0.90	0.49	1.63		0.93	0.50	1.73	
stage 3a	0.85	0.31	2.30		0.80	0.28	2.33	
stage 3b	0.90	0.28	2.89		0.95	0.29	3.10	
stage 4	0.93	0.07	12.52		0.94	0.07	13.17	
stage 5	0.51	0.03	8.70	0.97	1.85	0.11	31.66	0.97
**Sex**								
female	0.84	0.46	1.52		0.97	0.52	1.83	
male	0.86	0.47	1.57	0.94	0.82	0.44	1.55	0.63
**Individual DOAC**								
dabigatran	1.59	0.23	11.07		1.19	0.15	9.34	
rivaroxaban	0.72	0.40	1.30		0.91	0.48	1.70	
apixaban	1.19	0.55	2.58		1.01	0.47	2.15	
edoxaban	0.32	0.08	1.24	0.12	0.42	0.10	1.76	0.54
**Order of initiation**								
Initiation of DOAC	1.18	0.38	3.72		1.35	0.46	3.96	
Initiation of amiodarone/diltiazem	1.01	0.56	1.83		1.02	0.55	1.87	
Both drugs initiate together	0.46	0.22	0.97	0.07	0.47	0.20	1.11	0.09
**Level of dose***								
low dose	0.65	0.30	1.40		0.61	0.26	1.42	
high dose	0.77	0.43	1.40	0.65	0.82	0.45	1.51	0.45

*Only restricted the cohort to people with atrial fibrillation in this analysis

The power for conducting subgroup analyses for bodyweight, and polypharmacy was inadequate for either atorvastatin/simvastatin.

Table S16. Subgroup analysis for the association between concomitant use of DOAC and atorvastatin/simvastatin and other bleeding, compared with DOAC and other statins in cohort study

	Atorvastatin				Simvastatin			
Subgroup	HR	99% lower CI	99% Upper CI	Interaction p-value	HR	99% lower CI	99% Upper CI	Interaction p-value
**Age at cohort entry**								
≥ 18 -< 65	0.99	0.29	3.38		0.89	0.21	3.71	
≥ 65 -< 75	1.32	0.58	3.02		1.21	0.51	2.87	
≥ 75	1.47	0.82	2.64	0.76	1.52	0.81	2.85	0.64
**Potential indication**								
Atrial fibrillation	1.67	0.97	2.89	0.06	1.67	0.91	3.06	0.12
Venous thromboembolism	1.27	0.47	3.44	0.86	1.76	0.64	4.82	0.50
**Polypharmacy**								
No of drugs <5	2.35	0.17	33.58		2.89	0.20	42.67	
No of drugs ≥5	1.34	0.85	2.10	0.59	1.35	0.83	2.20	0.48
**Renal function**								
no CKD	1.45	0.80	2.61		1.34	0.70	2.59	
stage 3a	1.69	0.49	5.85		1.57	0.44	5.65	
stage 3b	1.50	0.38	5.92		1.51	0.36	6.32	
stage 4	0.25	0.04	1.49		1.84	0.33	10.37	
stage 5	0.63	0.04	9.59	0.23	1.61	0.09	27.59	0.99
**Sex**								
female	1.42	0.68	2.98		1.25	0.58	2.67	
male	1.30	0.74	2.29	0.81	1.47	0.79	2.73	0.67
**Individual DOAC**								
dabigatran	2.12	0.14	31.38		2.47	0.17	36.00	
rivaroxaban	0.84	0.49	1.45		1.03	0.56	1.89	
apixaban	2.28	0.93	5.57		1.91	0.81	4.51	
edoxaban	4.98	0.37	66.05	0.04	4.08	0.30	56.00	0.27
**Order of initiation**								
Initiation of DOAC	2.06	0.68	6.23		2.41	0.79	7.33	
Initiation of amiodarone/diltiazem	1.36	0.75	2.47		1.26	0.66	2.38	
Both drugs initiate together	0.94	0.40	2.21	0.34	1.14	0.45	2.87	0.34
**Level of dose***								
low dose	1.64	0.63	4.31		1.82	0.60	5.51	
high dose	1.70	0.87	3.34	0.94	1.57	0.76	3.23	0.77

*Only restricted the cohort to people with atrial fibrillation in this analysis

The power for conducting subgroup analyses for bodyweight was inadequate for either atorvastatin/simvastatin.

Figure S12. Subgroup analysis by level of dose of DOAC for the concomitant use of DOAC and atorvastatin among people with atrial fibrillation in case-crossover study

Ischaemic stroke

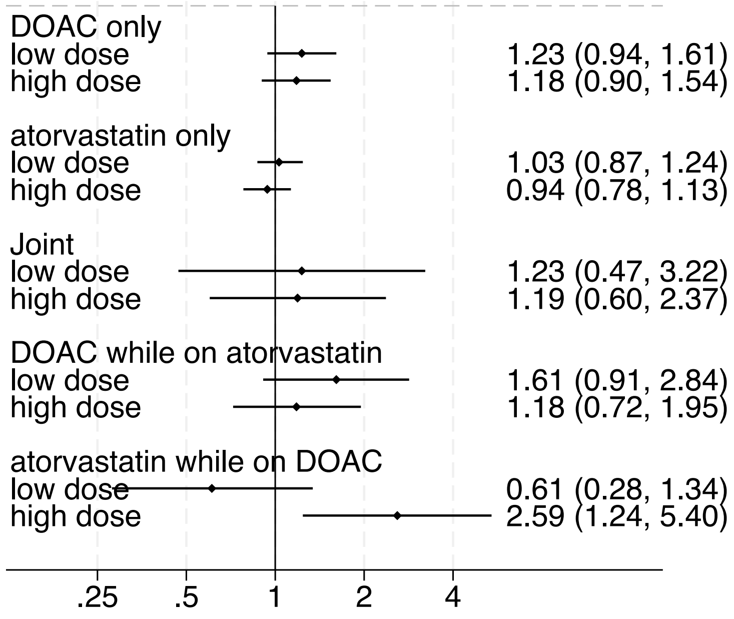

	p-value (combination A)	p-value (combination C)
Low dose	0.09	0.28
High dose	<0.01	0.99

Myocardial infarction

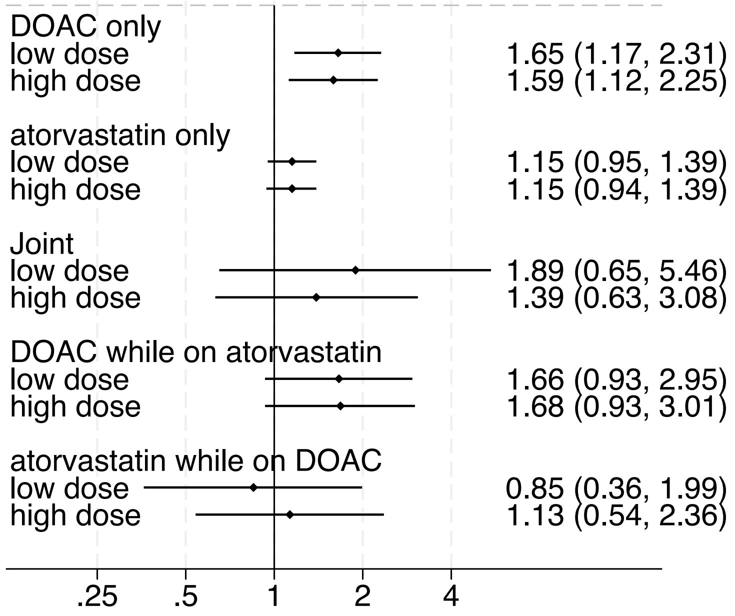

	p-value (combination A)	p-value (combination C)
Low dose	0.38	0.98
High dose	0.96	0.84

Venous thromboembolism

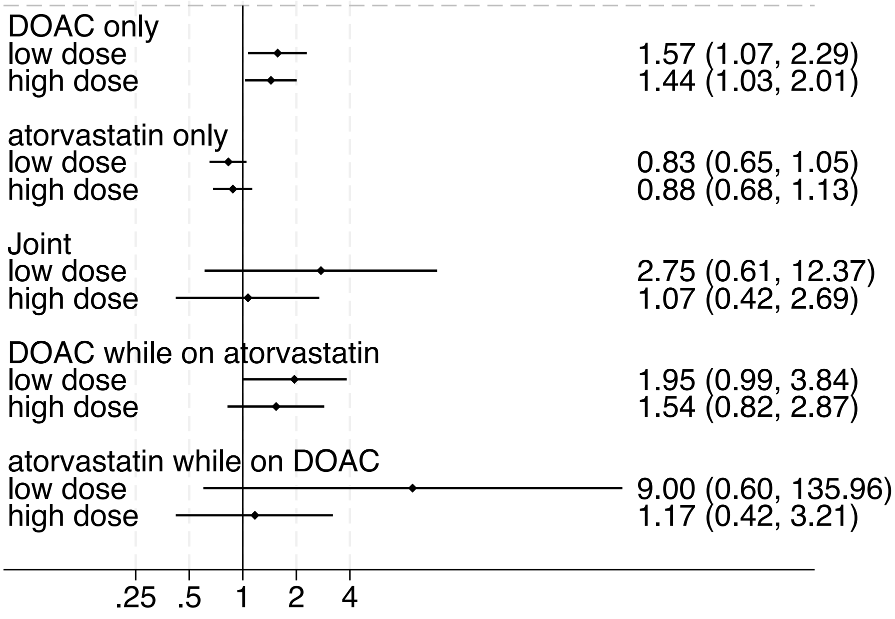

	p-value (combination A)	p-value (combination C)
Low dose	0.02	0.46
High dose	0.49	0.82

Intracranial bleeding

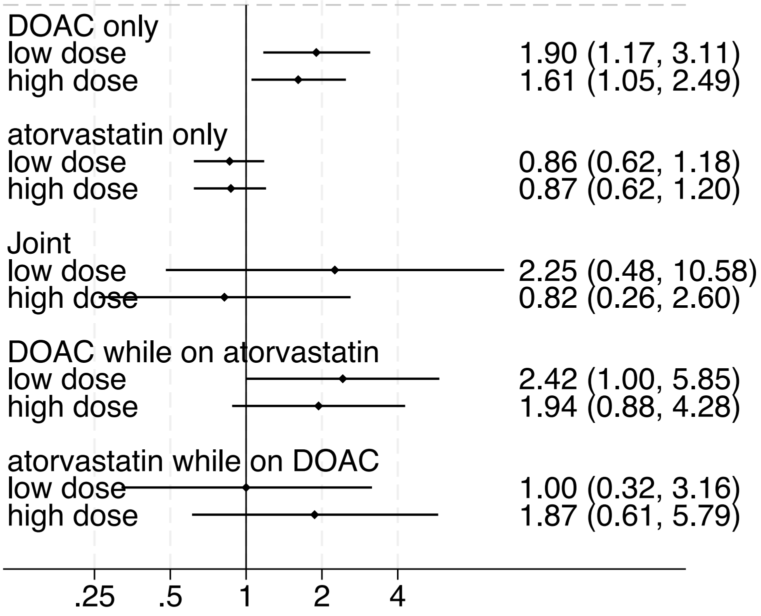

	p-value (combination A)	p-value (combination C)
Low dose	0.74	0.54
High dose	0.09	0.60

Gastrointestinal bleeding

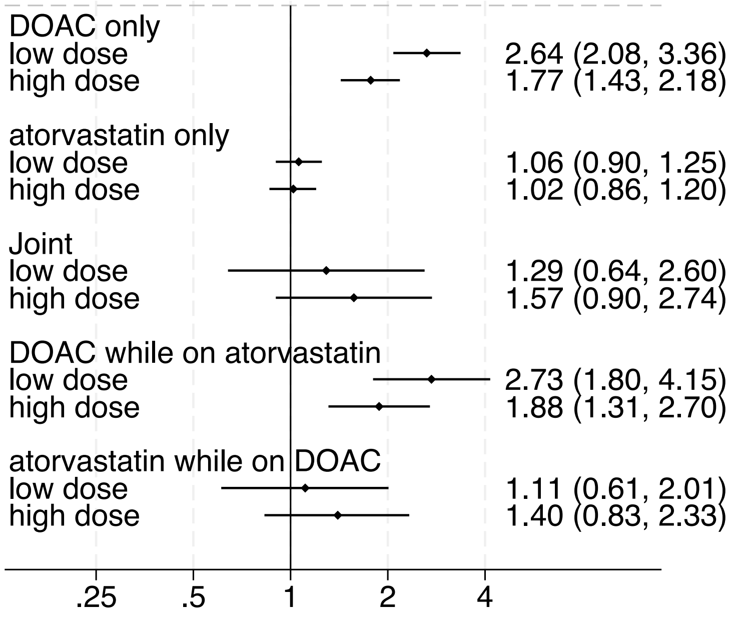

	p-value (combination A)	p-value (combination C)
Low dose	0.85	0.86
High dose	0.13	0.69

Other bleeding

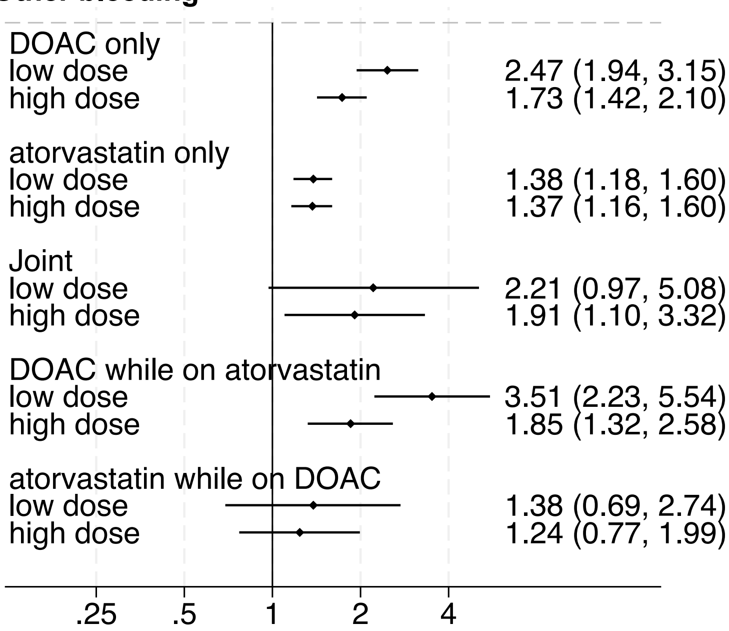

	p-value (combination A)	p-value (combination C)
Low dose	0.99	0.08
High dose	0.62	0.66

Cardiovascular mortality

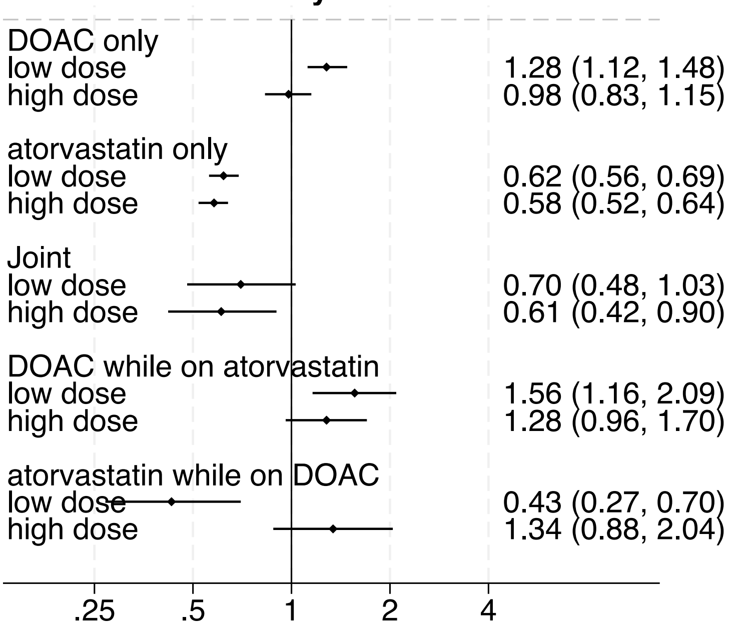

	p-value (combination A)	p-value (combination C)
Low dose	0.06	0.13
High dose	<0.01	0.04

All-cause mortality

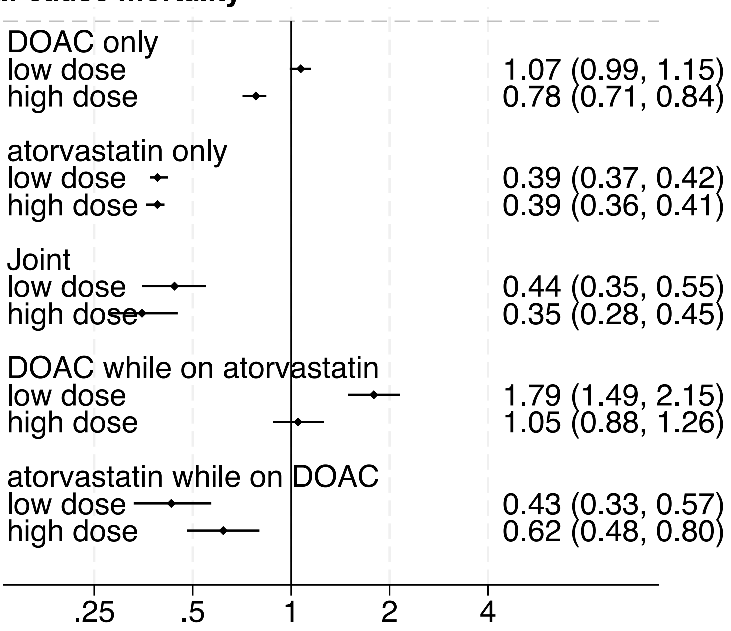

	p-value (combination A)	p-value (combination C)
Low dose	0.33	<0.01
High dose	<0.01	<0.01

All figures are shown as Odds ratio (99% Confidence interval). Combination A and C refers to testing the equality of two coefficients shown in Supplementary Information S4.

Figure S13. Subgroup analysis by level of dose of DOAC for the concomitant use of DOAC and simvastatin among people with atrial fibrillation in case-crossover study

Ischaemic stroke

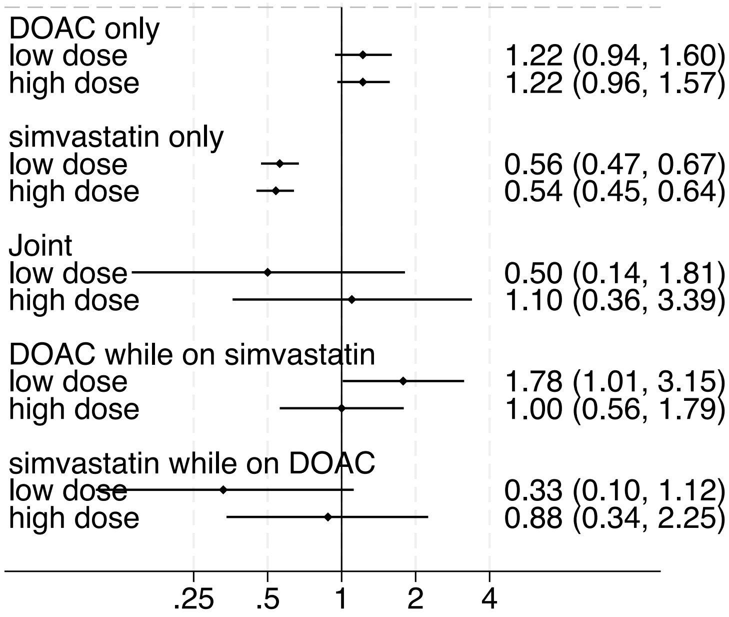

	p-value (combination A)	p-value (combination C)
Low dose	0.27	0.12
High dose	0.19	0.41

Myocardial infarction

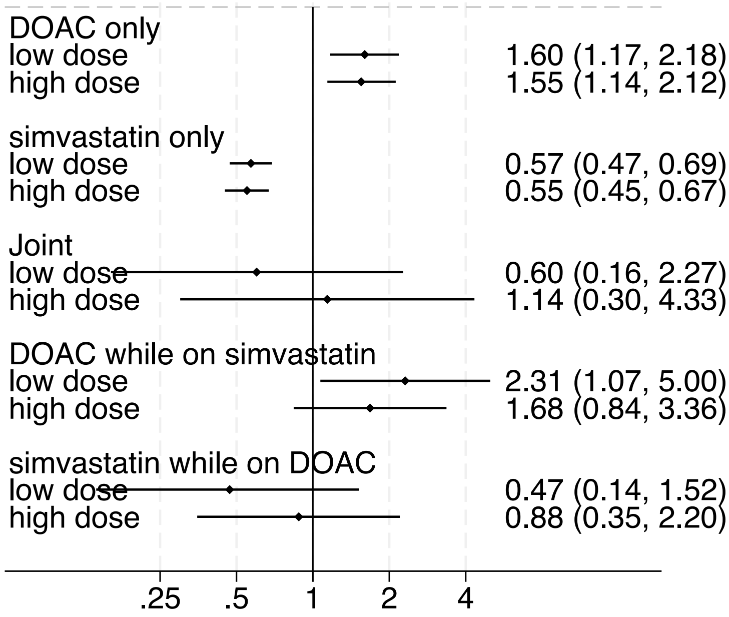

	p-value (combination A)	p-value (combination C)
Low dose	0.66	0.25
High dose	0.19	0.79

Venous thromboembolism

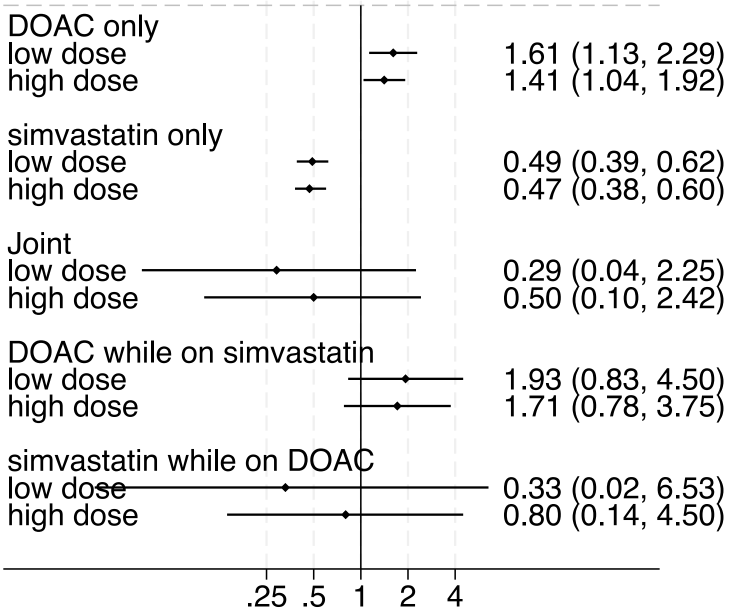

	p-value (combination A)	p-value (combination C)
Low dose	0.74	0.61
High dose	0.44	0.57

Intracranial bleeding

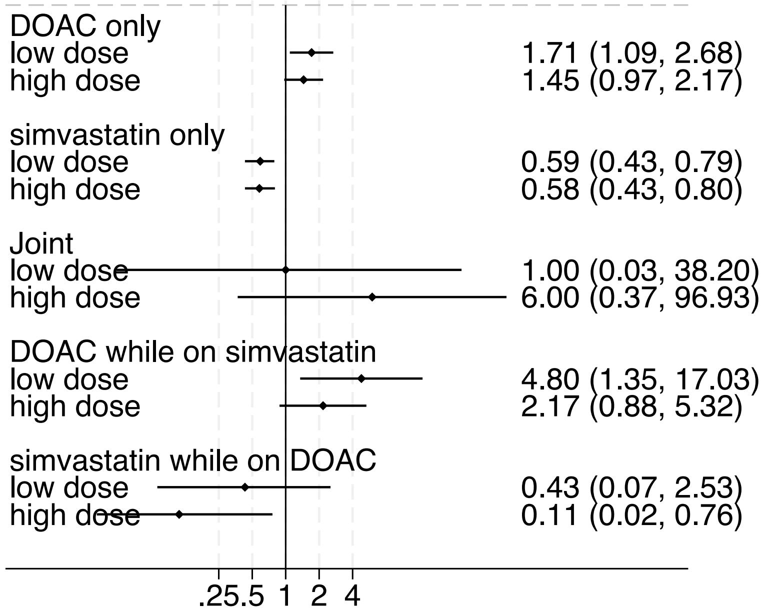

	p-value (combination A)	p-value (combination C)
Low dose	0.66	0.05
High dose	0.03	0.29

Gastrointestinal bleeding

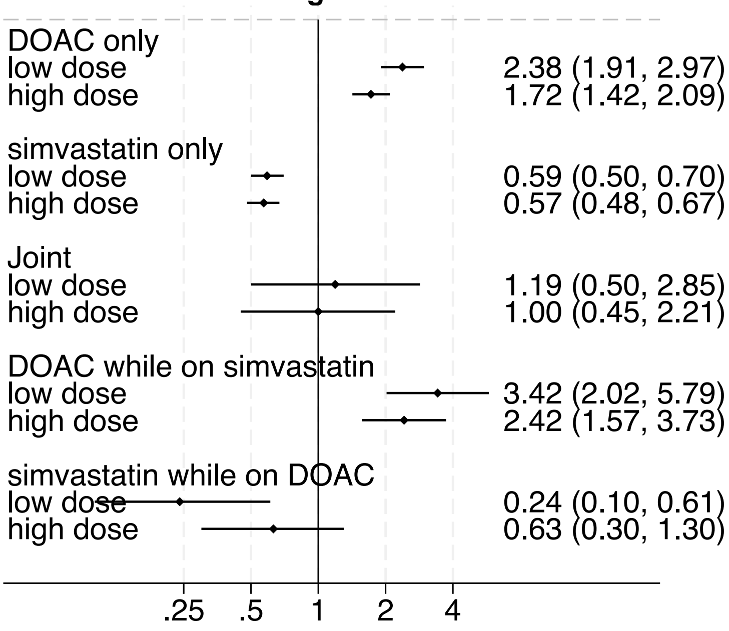

	p-value (combination A)	p-value (combination C)
Low dose	0.01	0.10
High dose	0.73	0.07

Other bleeding

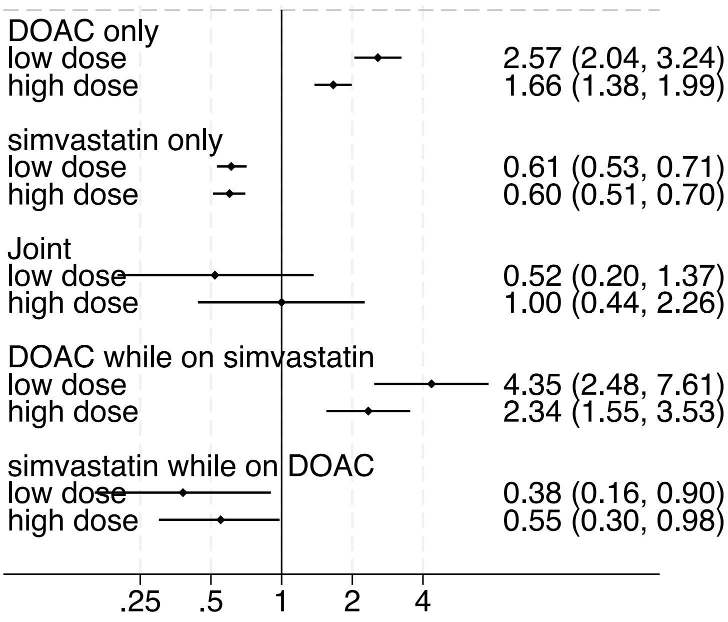

	p-value (combination A)	p-value (combination C)
Low dose	0.15	0.03
High dose	0.69	0.05

Cardiovascular mortality

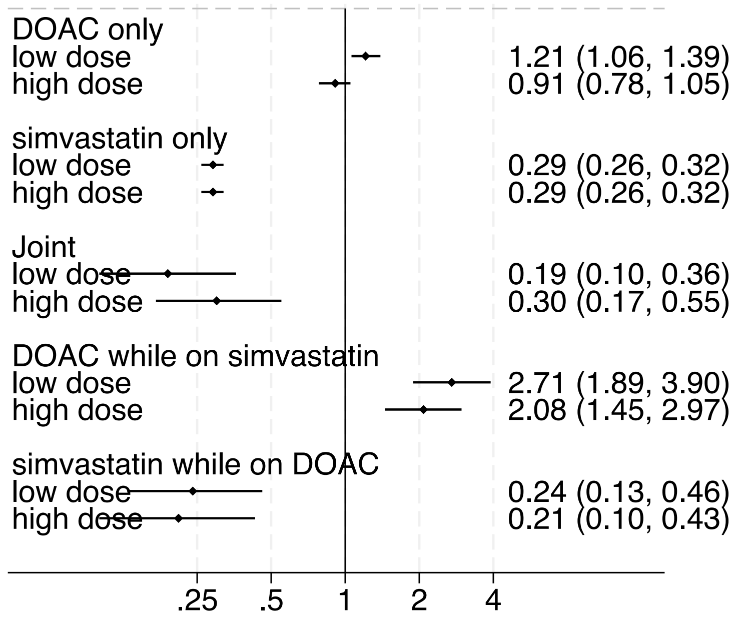

	p-value (combination A)	p-value (combination C)
Low dose	0.45	<0.01
High dose	0.27	<0.01

All-cause mortality

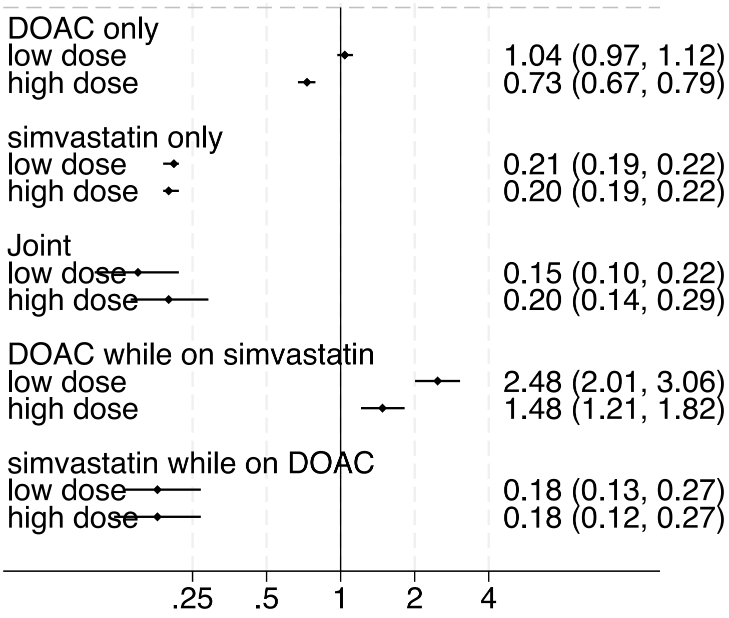

	p-value (combination A)	p-value (combination C)
Low dose	0.41	<0.01
High dose	0.45	<0.01

All figures are shown as Odds ratio (99% Confidence interval)
Combination A and C refers to testing the equality of two coefficients shown in Supplementary Information S4.

Figure S14. Subgroup analysis by different types of DOAC for the concomitant use of DOAC and atorvastatin among people with atrial fibrillation in case-crossover study

Ischaemic stroke

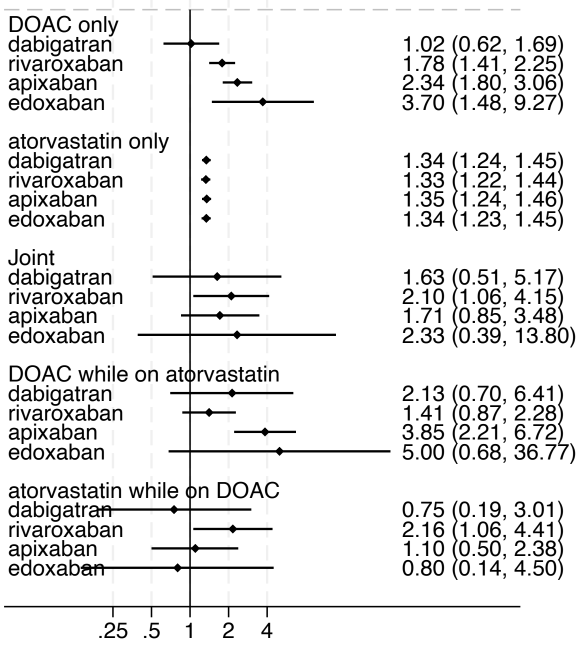

	p-value (combination A)	p-value (combination C)
dabigatran	0.28	0.12
rivaroxaban	0.08	0.25
apixaban	0.50	0.04
edoxaban	0.44	0.72

Myocardial infarction

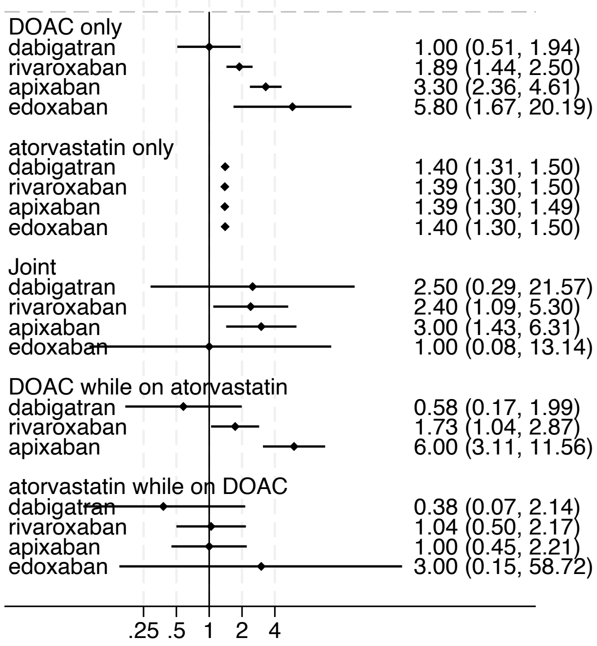

	p-value (combination A)	p-value (combination C)
dabigatran	0.05	0.32

rivaroxaban	0.31	0.69
apixaban	0.29	0.04
edoxaban	0.51	NA

Venous thromboembolism

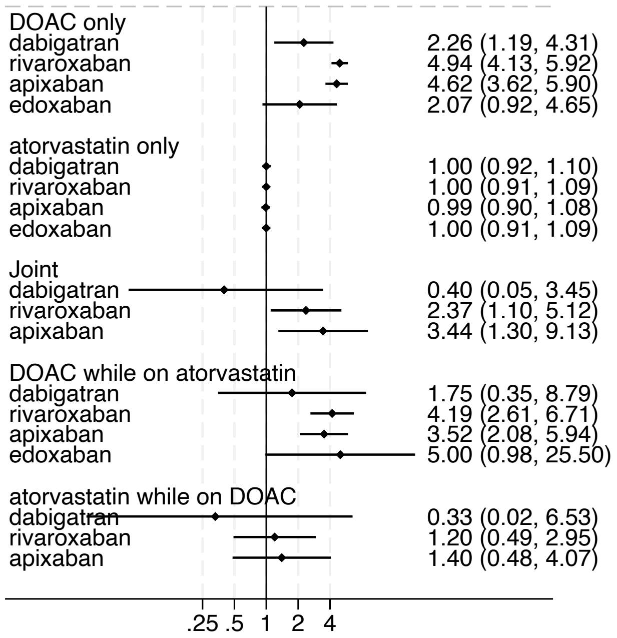

	p-value (combination A)	p-value (combination C)
dabigatran	0.34	0.70
rivaroxaban	0.60	0.40
apixaban	0.40	0.22
edoxaban	NA	0.21

Intracranial bleeding

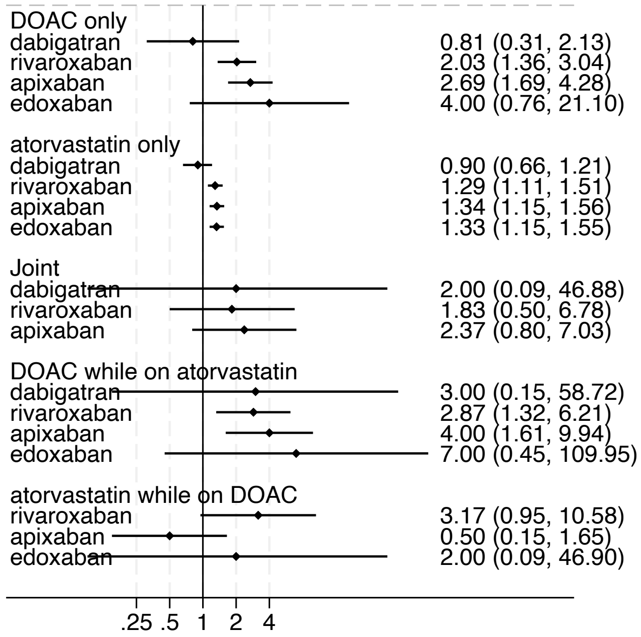

	p-value (combination A)	p-value (combination C)

dabigatran	NA	0.28
rivaroxaban	0.06	0.31
apixaban	0.03	0.32
edoxaban	0.74	0.65

Gastrointestinal bleeding

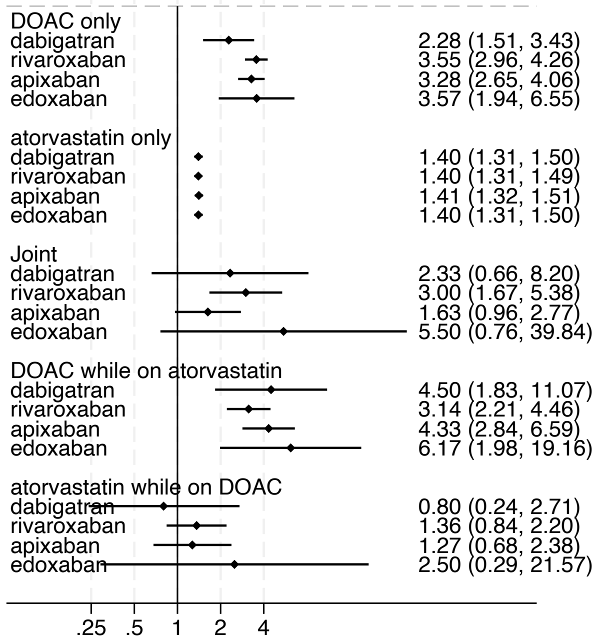

	p-value (combination A)	p-value (combination C)
dabigatran	0.24	0.08
rivaroxaban	0.89	0.42
apixaban	0.66	0.13
edoxaban	0.49	0.27

Other bleeding

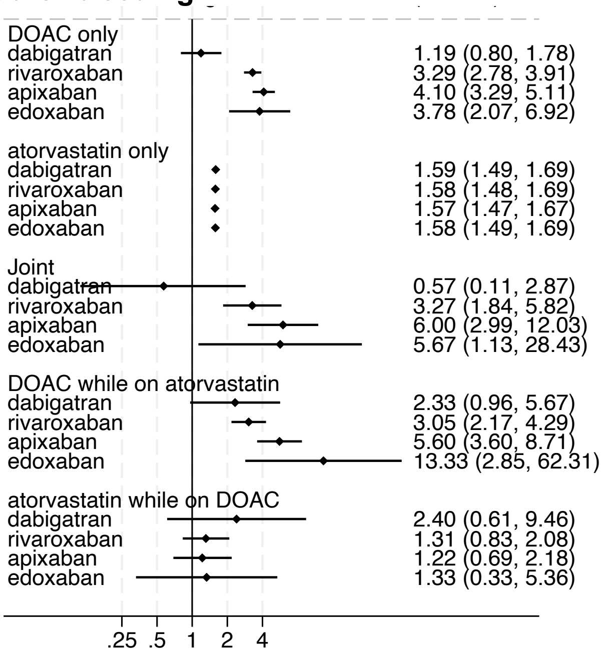

	p-value (combination A)	p-value (combination C)
dabigatran	0.44	0.08
rivaroxaban	0.29	0.61
apixaban	0.27	0.10
edoxaban	0.75	0.05

Cardiovascular mortality

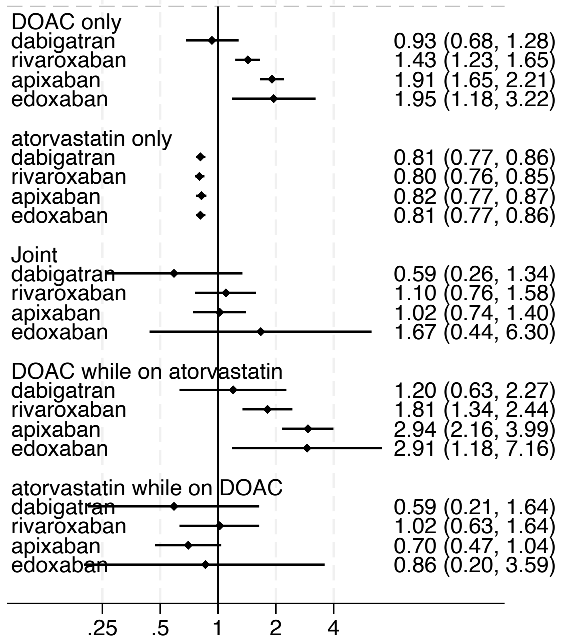

	p-value (combination A)	p-value (combination C)
dabigatran	0.41	0.36
rivaroxaban	0.21	0.06
apixaban	0.30	<0.01
edoxaban	0.92	0.32

All-cause mortality

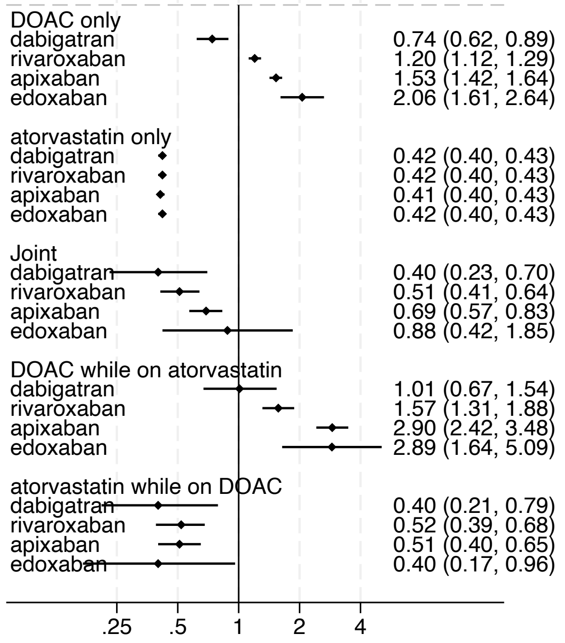

	p-value (combination A)	p-value (combination C)
dabigatran	0.89	0.08
rivaroxaban	0.04	<0.01
apixaban	0.03	<0.01
edoxaban	0.90	0.16

All figures are shown as Odds ratio (99% Confidence interval)
Combination A and C refers to testing the equality of two coefficients shown in Supplementary Information S4.

Figure S15. Subgroup analysis by different types of DOAC for the concomitant use of DOAC and simvastatin among people with atrial fibrillation in case-crossover study

Ischaemic stroke

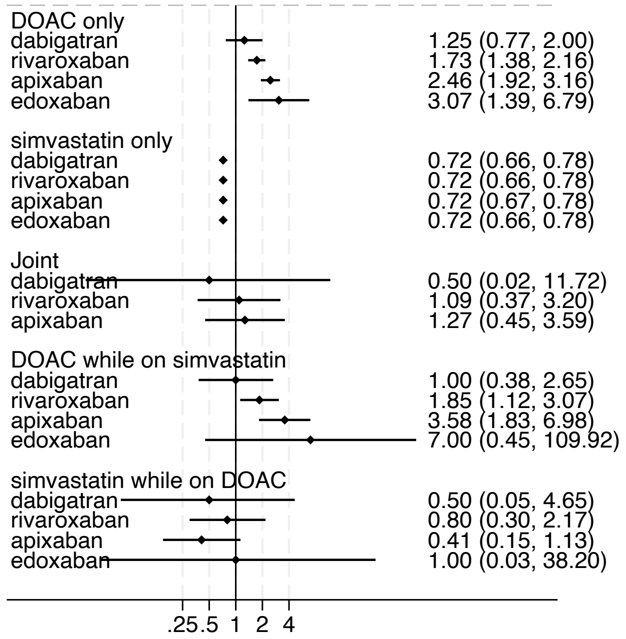

	p-value (combination A)	p-value (combination C)
dabigatran	0.68	0.60
rivaroxaban	0.78	0.74
apixaban	0.15	0.18
edoxaban	0.81	0.46

Myocardial infarction

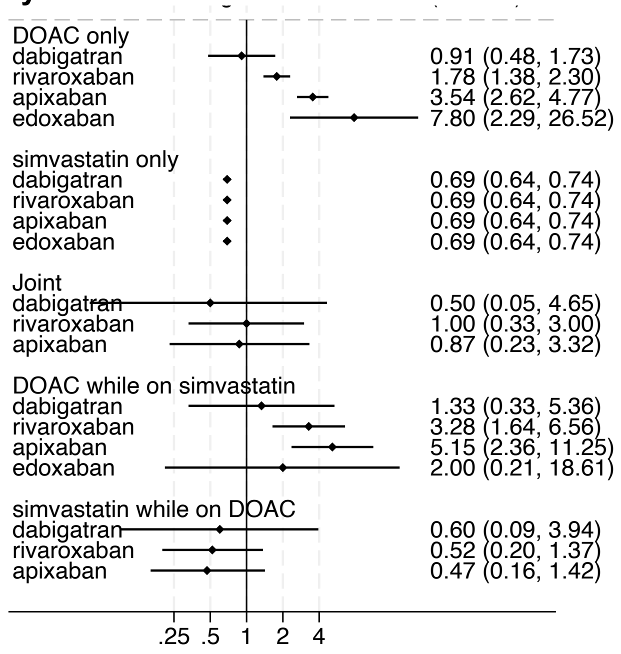

	p-value (combination A)	p-value (combination C)
dabigatran	0.85	0.52
rivaroxaban	0.47	0.03

apixaban	0.37	0.25
edoxaban	NA	0.17

Venous thromboembolism

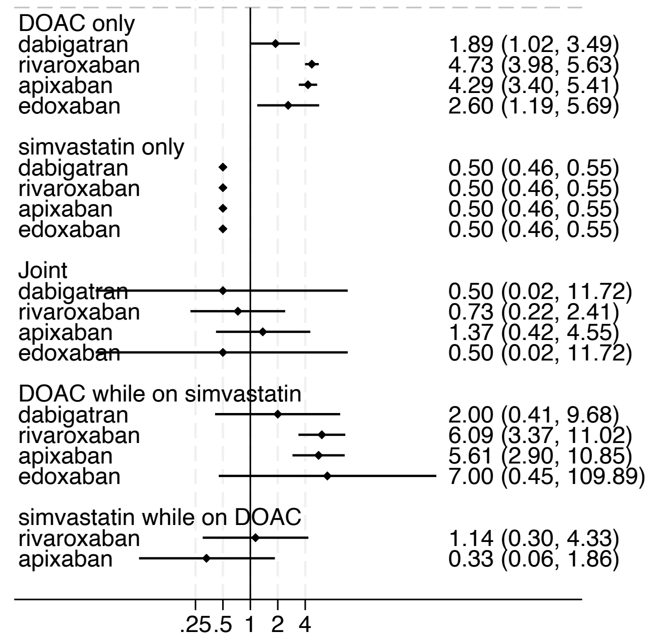

	p-value (combination A)	p-value (combination C)
dabigatran	NA	0.93
rivaroxaban	0.11	0.29
apixaban	0.54	0.32
edoxaban	NA	0.37

Intracranial bleeding

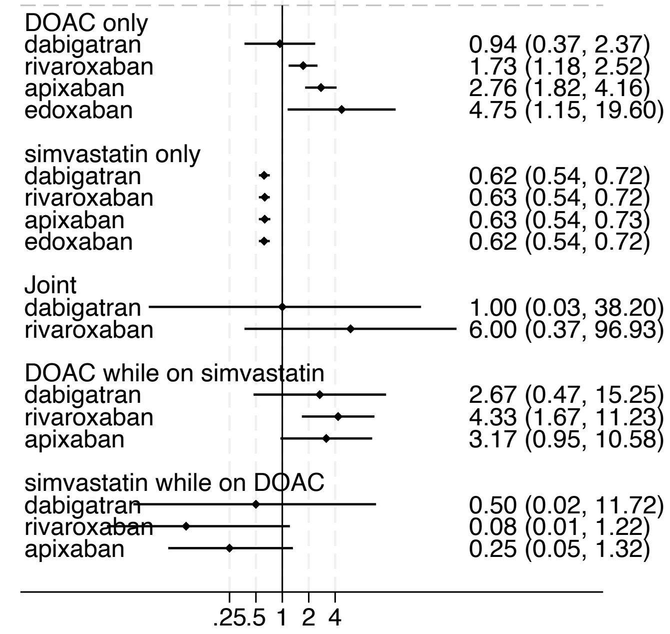

	p-value (combination A)	p-value (combination C)
dabigatran	0.86	0.17
rivaroxaban	0.05	0.02
apixaban	0.16	0.78
edoxaban	NA	NA

Gastrointestinal bleeding

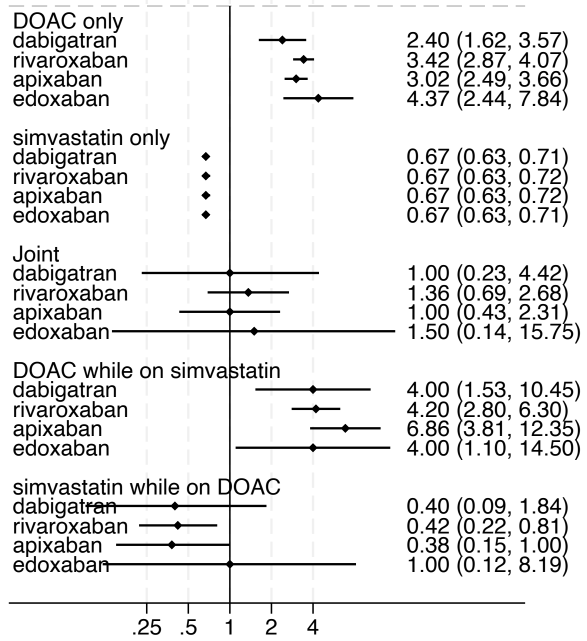

	p-value (combination A)	p-value (combination C)
dabigatran	0.39	0.21
rivaroxaban	0.07	0.23
apixaban	0.14	<0.01
edoxaban	0.62	0.87

Other bleeding

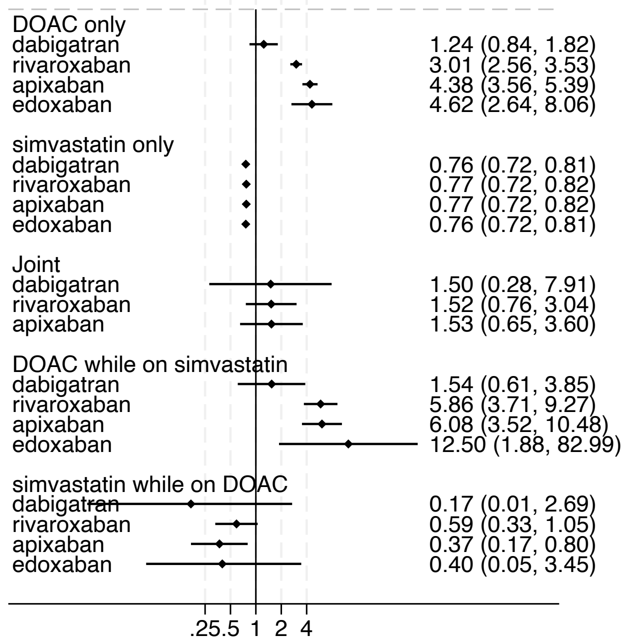

	p-value (combination A)	p-value (combination C)
dabigatran	0.16	0.57
rivaroxaban	0.25	<0.01
apixaban	0.01	0.15
edoxaban	0.44	0.19

Cardiovascular mortality

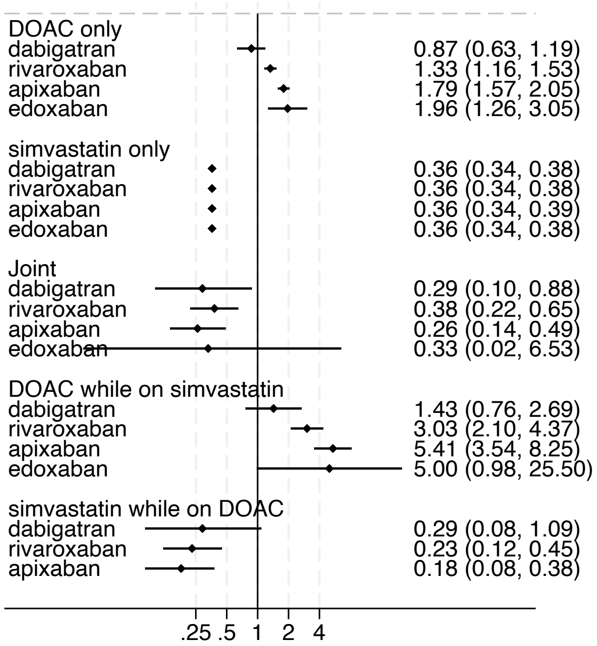

	p-value (combination A)	p-value (combination C)
dabigatran	0.70	0.07
rivaroxaban	0.08	<0.01
apixaban	0.01	<0.01
edoxaban	NA	0.15

All-cause mortality

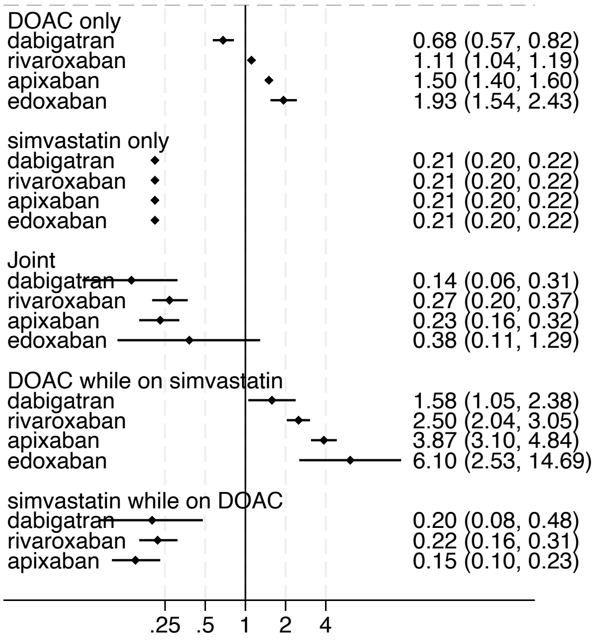

	p-value (combination A)	p-value (combination C)
dabigatran	0.86	<0.01
rivaroxaban	0.67	<0.01
apixaban	0.04	<0.01
edoxaban	NA	<0.01

All figures are shown as Odds ratio (99% Confidence interval)
Combination A and C refers to testing the equality of two coefficients shown in Supplementary Information S4.

Figure S16. Results of case-crossover study investigating the concomitant use of DOACs and other statins

Ischaemic stroke

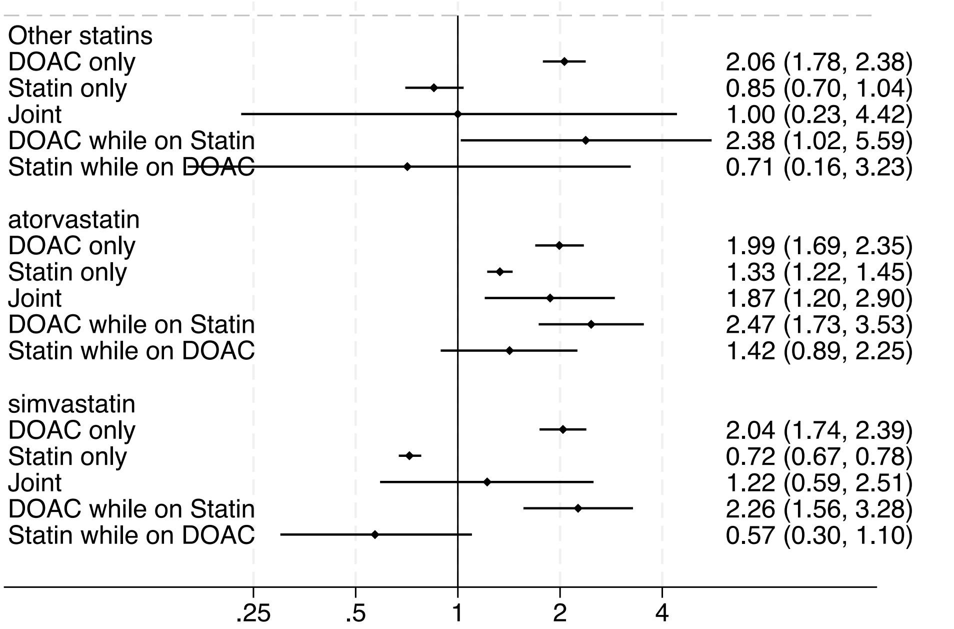

p-value (combination A) for other statins	p-value (combination C) for other statins
0.76	0.66

Myocardial infarction

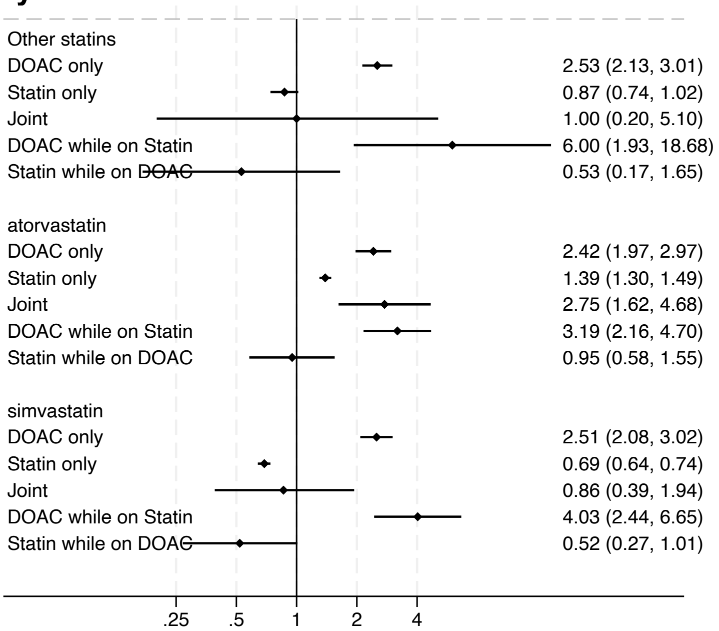

p-value (combination A) for other statins	p-value (combination C) for other statins
0.27	0.05

Venous thromboembolism

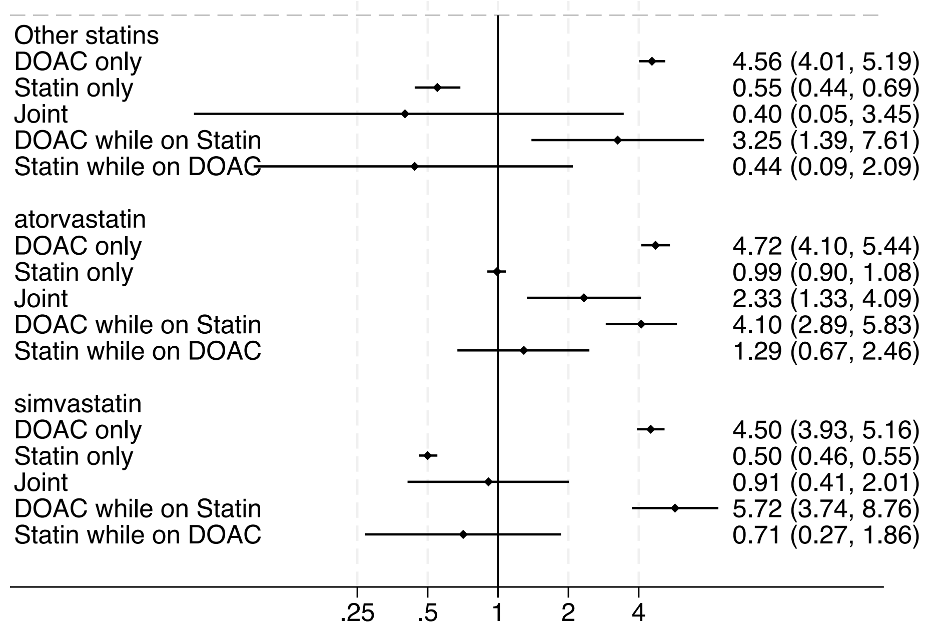

p-value (combination A) for other statins	p-value (combination C) for other statins
0.72	0.31

Intracranial bleeding

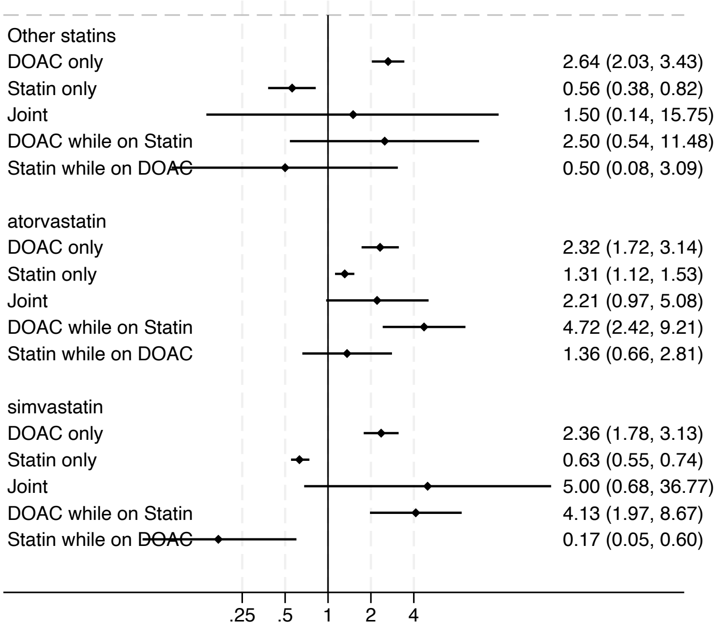

p-value (combination A) for other statins	p-value (combination C) for other statins
0.88	0.93

Gastrointestinal bleeding

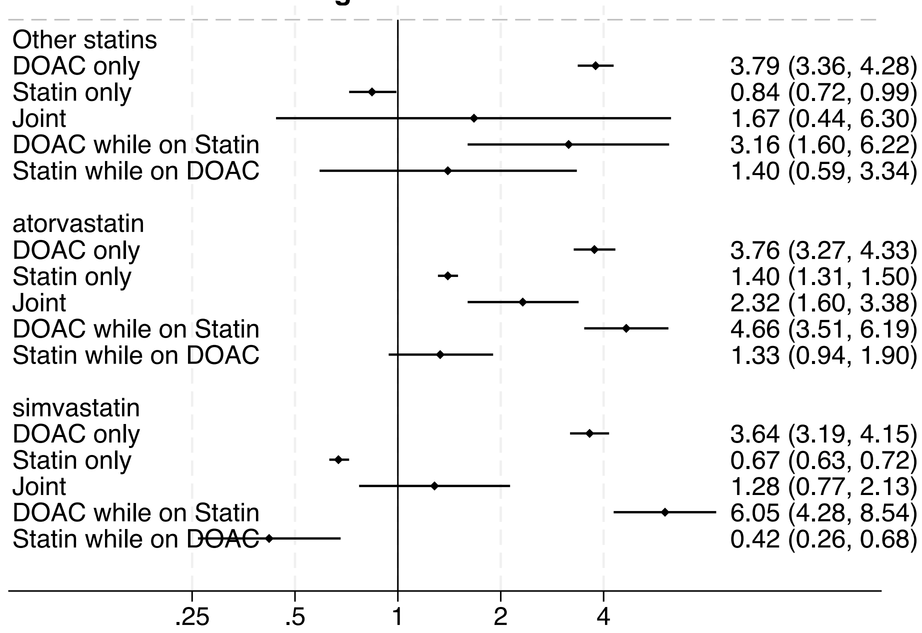

p-value (combination A) for other statins	p-value (combination C) for other statins
0.14	0.49

Other bleeding

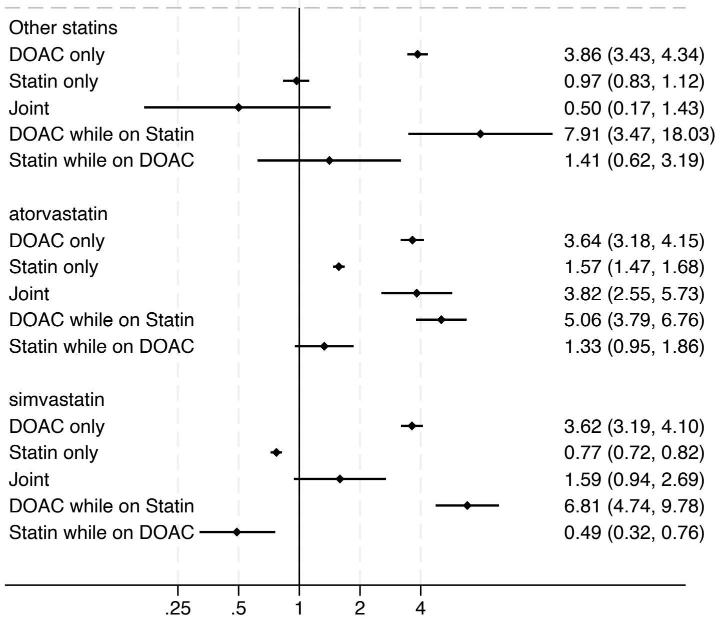

p-value (combination A) for other statins	p-value (combination C) for other statins
0.24	0.03

Cardiovascular mortality

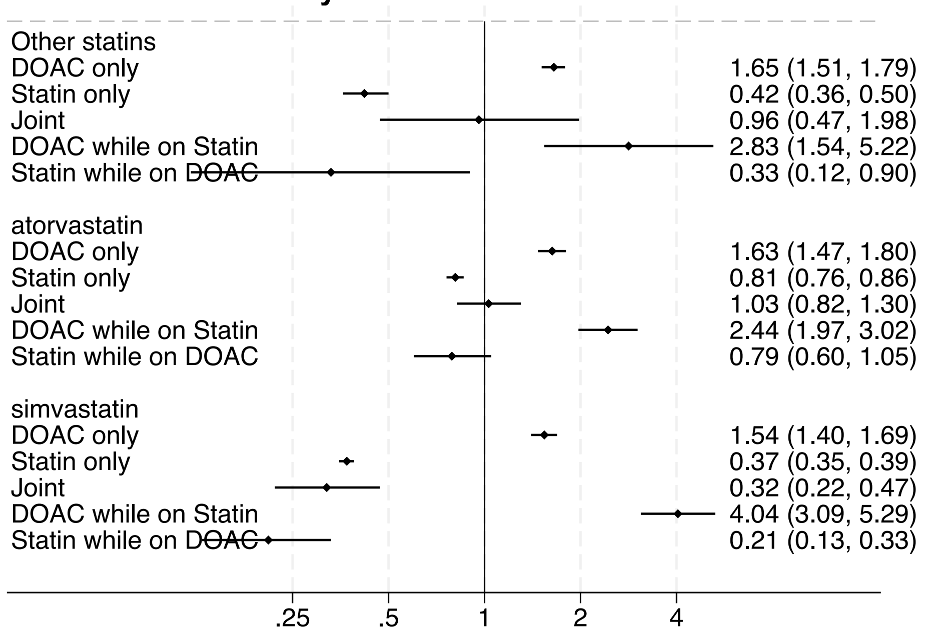

p-value (combination A) for other statins	p-value (combination C) for other statins
0.54	0.02

All-cause mortality

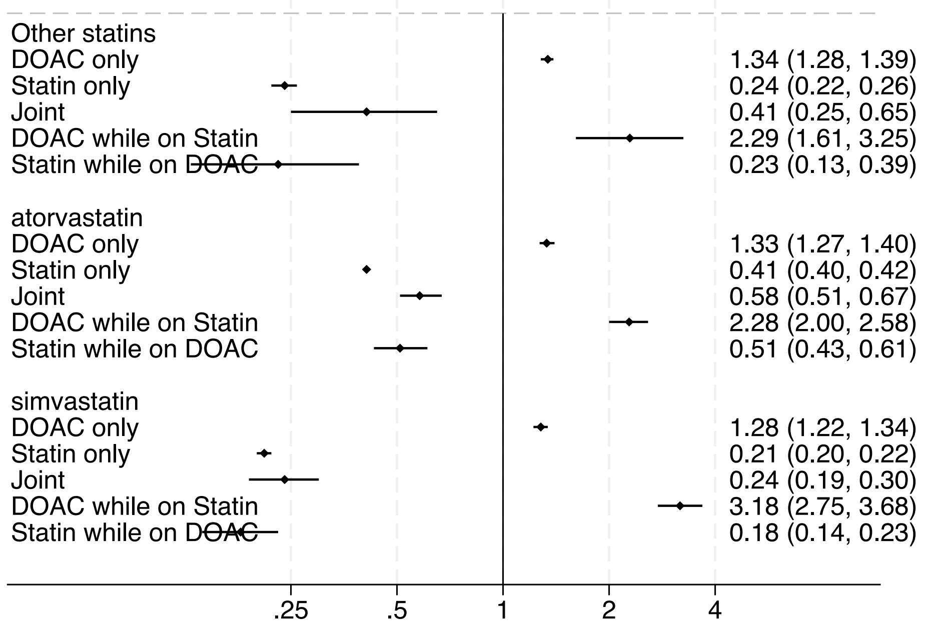

p-value (combination A) for other statins	p-value (combination C) for other statins
0.77	<0.01

All figures are shown as Odds ratio (99% Confidence interval)
Combination A and C refers to testing the equality of two coefficients shown in Supplementary Information S4.

Figure S17. Duration of effects for concomitant use of DOAC and atorvastatin in case-crossover study

Ischaemic stroke

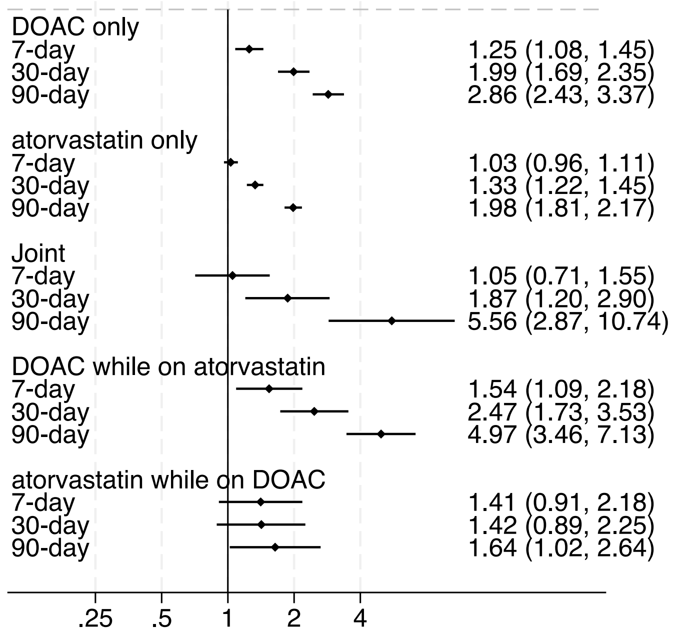

	p-value (combination A)	p-value (combination C)
7-day	0.07	0.15
30-day	0.74	0.16
90-day	0.31	<0.01

Myocardial infarction

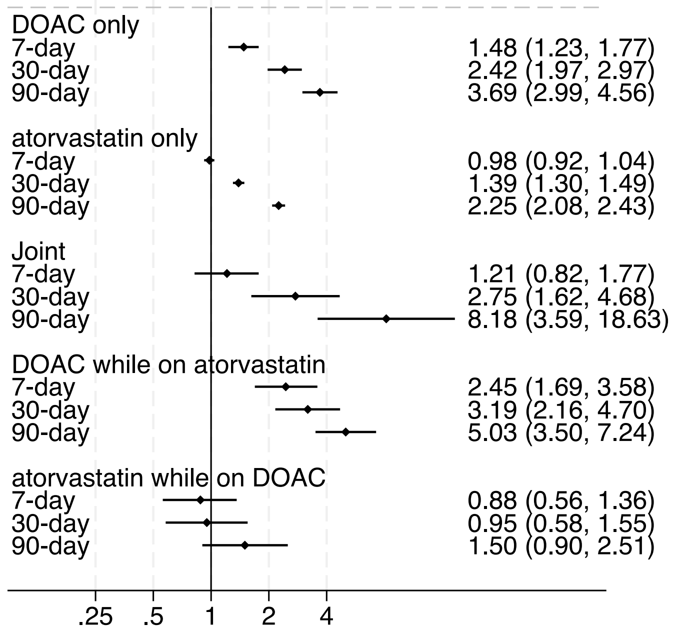

	p-value (combination A)	p-value (combination C)
7-day	0.53	<0.01
30-day	0.05	0.11
90-day	0.05	0.06

Venous thromboembolism

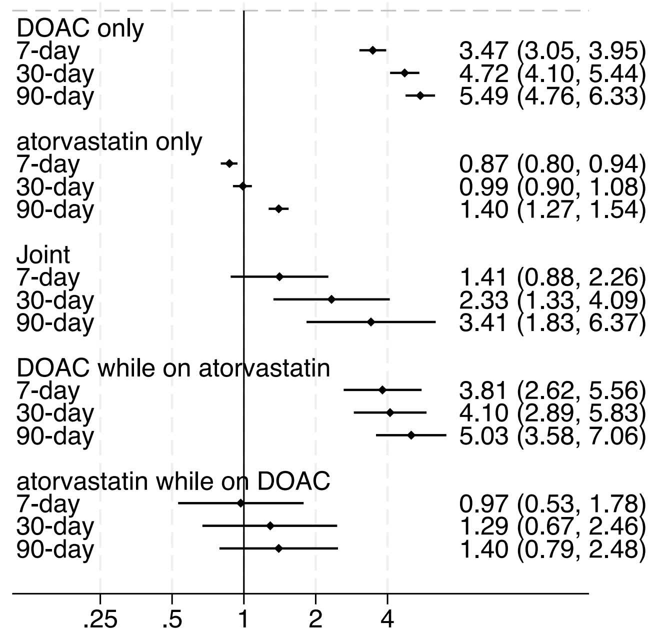

	p-value (combination A)	p-value (combination C)
7-day	0.63	0.55
30-day	0.30	0.34
90-day	0.99	0.54

Intracranial bleeding

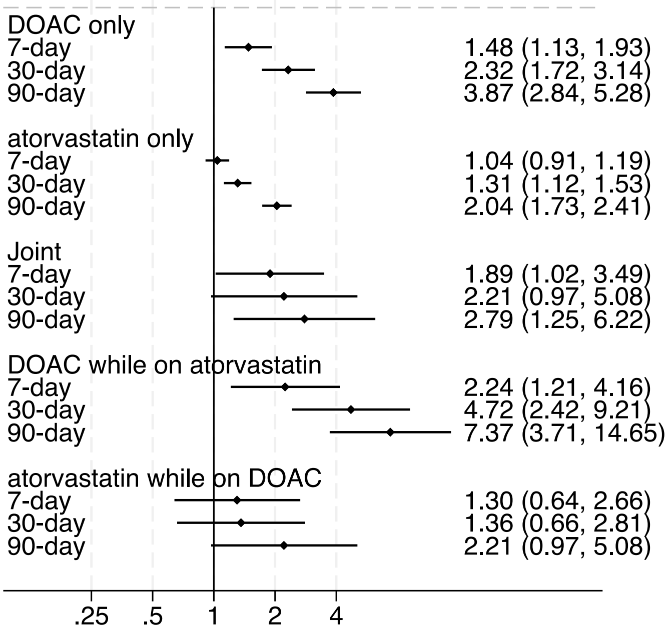

	p-value (combination A)	p-value (combination C)
7-day	0.42	0.11
30-day	0.89	0.01
90-day	0.80	0.03

Gastrointestinal bleeding

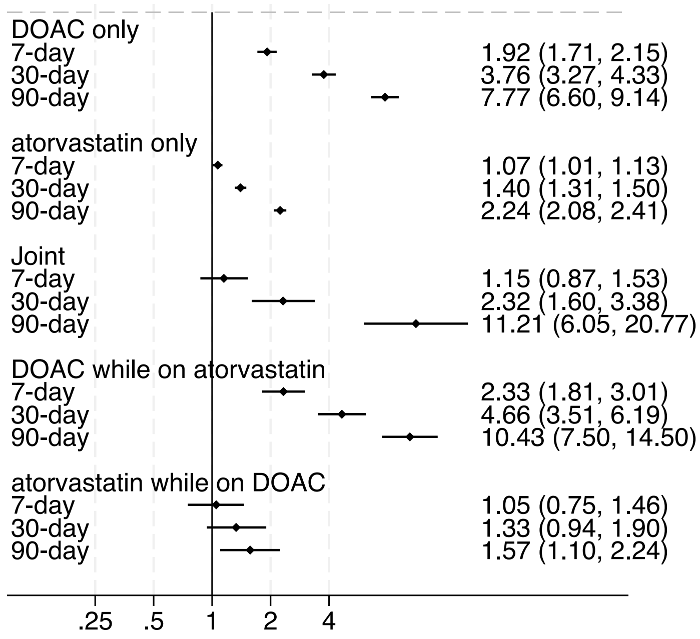

	p-value (combination A)	p-value (combination C)
7-day	0.90	0.07
30-day	0.71	0.08
90-day	0.01	0.04

Other bleeding

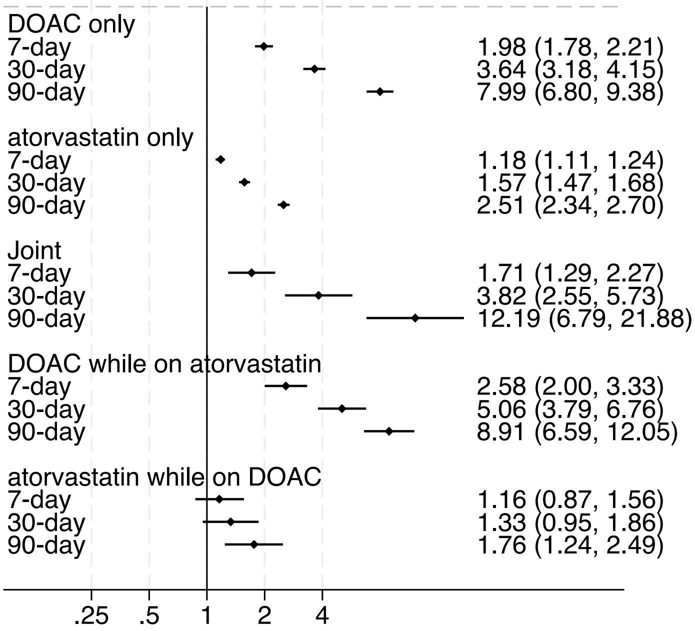

	p-value (combination A)	p-value (combination C)
7-day	0.91	0.01
30-day	0.21	<0.01
90-day	<0.01	0.41

Cardiovascular mortality

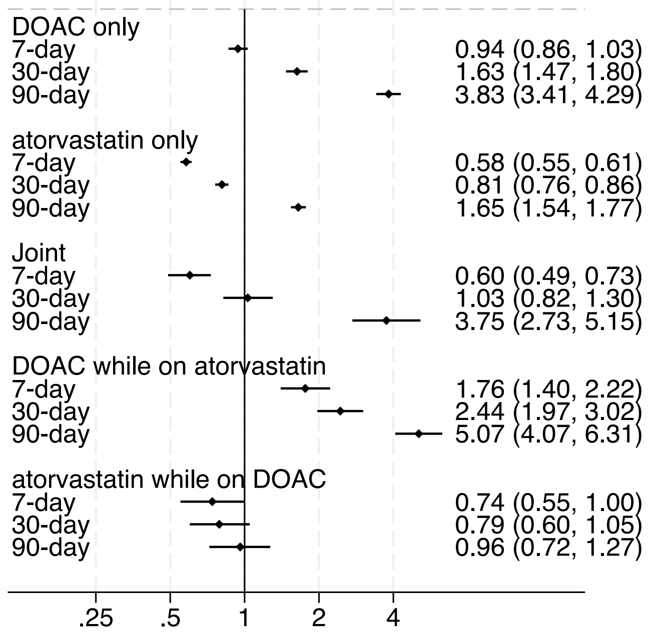

	p-value (combination A)	p-value (combination C)
7-day	0.04	<0.01
30-day	0.87	<0.01
90-day	<0.01	<0.01

All-cause mortality

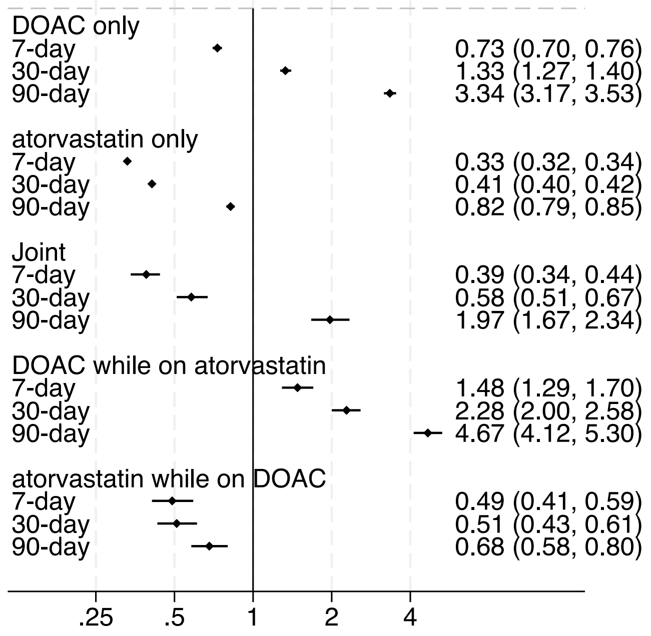

	p-value (combination A)	p-value (combination C)
7-day	<0.01	<0.01
30-day	<0.01	<0.01
90-day	<0.01	<0.01

All figures are shown as Odds ratio (99% Confidence interval)

Combination A and C refers to testing the equality of two coefficients shown in Supplementary Information S4.

Figure S18. Duration of effects for concomitant use of DOAC and simvastatin in case-crossover study

Ischaemic stroke

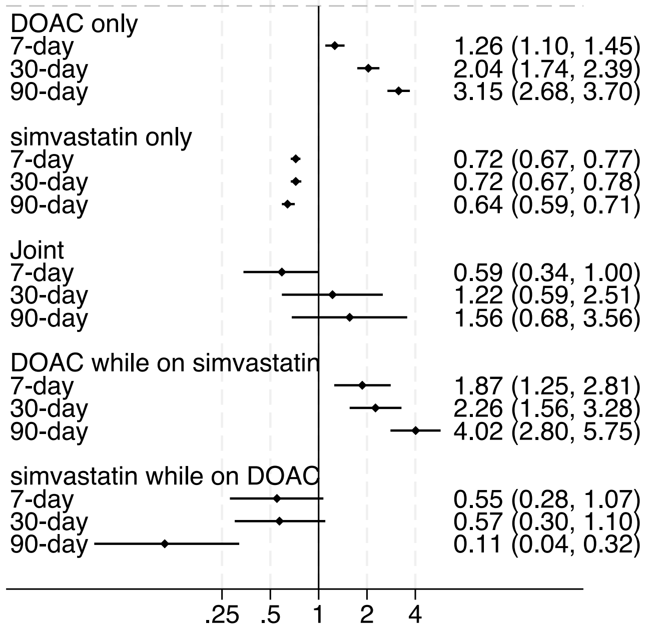

	p-value (combination A)	p-value (combination C)
7-day	0.30	0.02
30-day	0.37	0.51
90-day	<0.01	0.11

Myocardial infarction

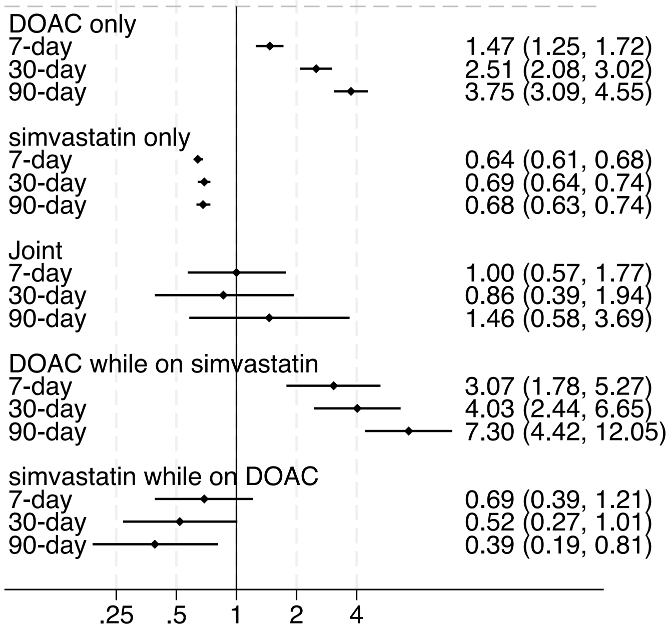

	p-value (combination A)	p-value (combination C)
7-day	0.77	<0.01
30-day	0.28	0.02
90-day	0.05	<0.01

Venous thromboembolism

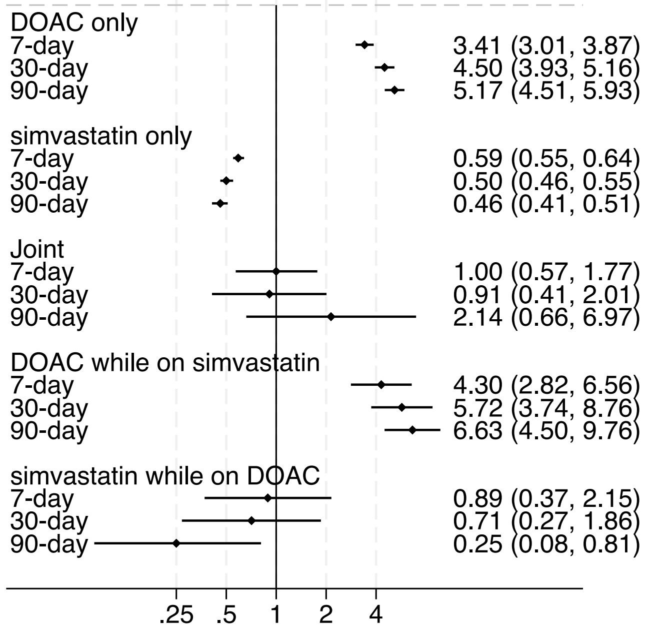

	p-value (combination A)	p-value (combination C)
7-day	0.24	0.17
30-day	0.37	0.17
90-day	0.18	0.12

Intracranial bleeding

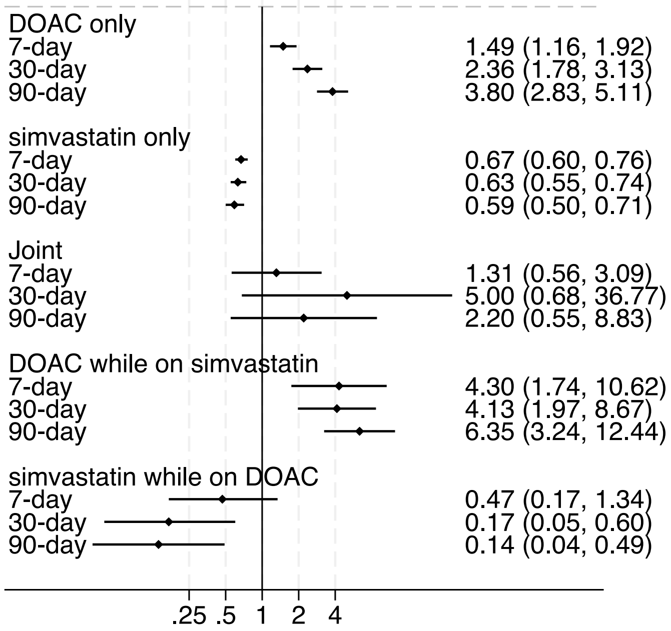

	p-value (combination A)	p-value (combination C)
7-day	0.39	<0.01
30-day	0.01	0.07
90-day	<0.01	0.07

Gastrointestinal bleeding

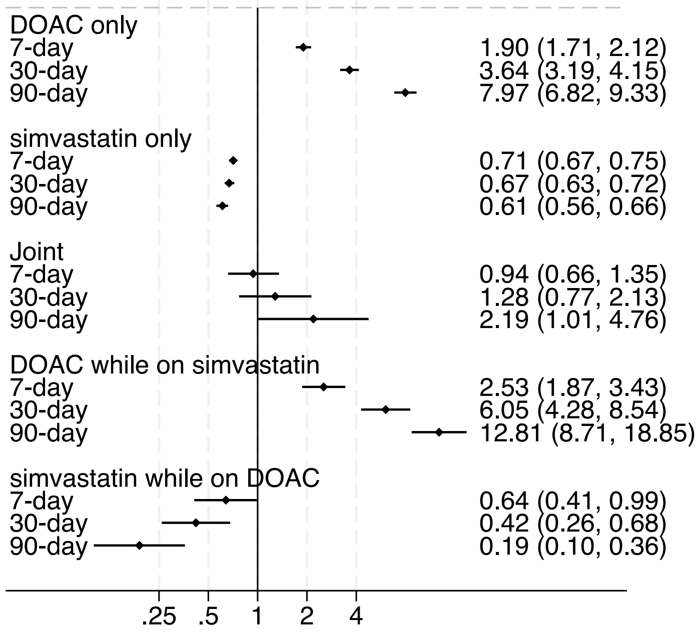

	p-value (combination A)	p-value (combination C)
7-day	0.52	0.02
30-day	0.01	<0.01
90-day	<0.01	<0.01

Other bleeding

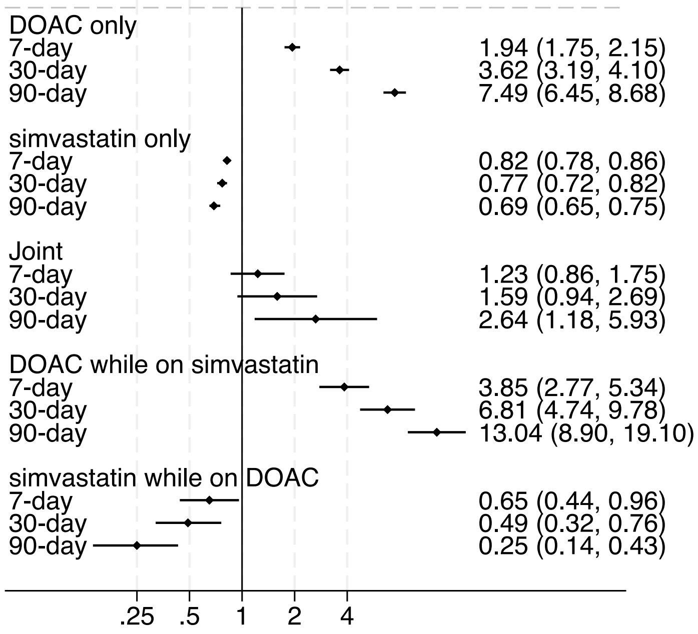

	p-value (combination A)	p-value (combination C)
7-day	0.13	<0.01
30-day	<0.01	<0.01
90-day	<0.01	<0.01

Cardiovascular mortality

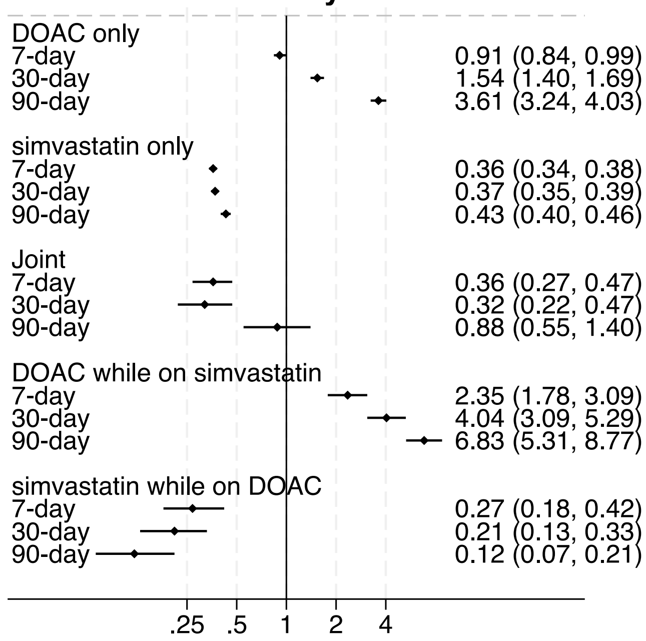

	p-value (combination A)	p-value (combination C)
7-day	0.10	<0.01
30-day	<0.01	<0.01
90-day	<0.01	<0.01

All-cause mortality

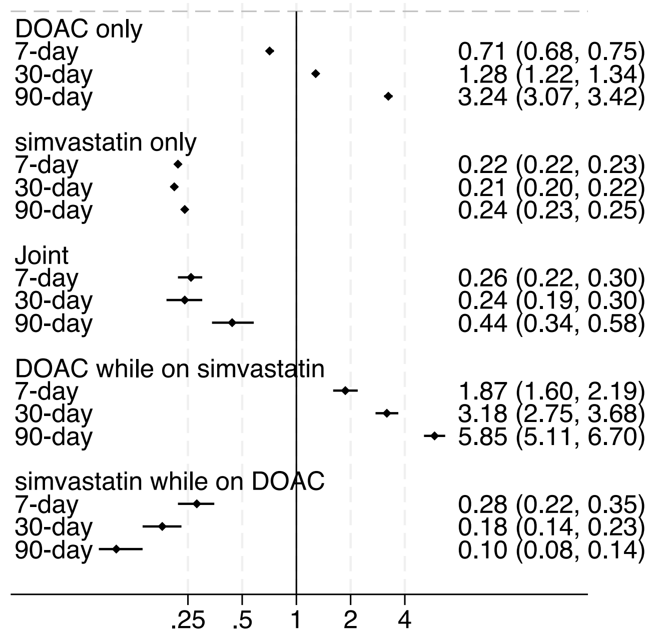

	p-value (combination A)	p-value (combination C)
7-day	0.01	<0.01
30-day	0.10	<0.01
90-day	<0.01	<0.01

All figures are shown as Odds ratio (99% Confidence interval)

Combination A and C refers to testing the equality of two coefficients shown in Supplementary Information S4.
